# Evolutionary analyses of myosin genes in trypanosomatids show a history of expansion, secondary losses and neofunctionalization

**DOI:** 10.1038/s41598-017-18865-y

**Published:** 2018-01-22

**Authors:** Denise Andréa Silva de Souza, Daniela Parada Pavoni, Marco Aurélio Krieger, Adriana Ludwig

**Affiliations:** 1 Laboratório de Genômica Funcional, Instituto Carlos Chagas- ICC/Fiocruz-, PR Curitiba, 81350-010 Brazil; 2 Programa de Pós-graduação em Biociências e Biotecnologia - ICC/Fiocruz-, PR Curitiba, 81350-010 Brazil; 3 Instituto de Biologia Molecular do Paraná, Curitiba, 81350-010 Brazil

## Abstract

Myosins are motor proteins that comprise a large and diversified family important for a broad range of functions. Two myosin classes, I and XIII, were previously assigned in Trypanosomatids, based mainly on the studies of *Trypanosoma cruzi*, *T*. *brucei* and *Leishmania major*, and important human pathogenic species; seven orphan myosins were identified in *T*. *cruzi*. Our results show that the great variety of *T*. *cruzi* myosins is also present in some closely related species and in *Bodo saltans*, a member of an early divergent branch of Kinetoplastida. Therefore, these myosins should no longer be considered “orphans”. We proposed the classification of a kinetoplastid-specific myosin group into a new class, XXXVI. Moreover, our phylogenetic data suggest that a great repertoire of myosin genes was present in the last common ancestor of trypanosomatids and *B*. *saltans*, mainly resulting from several gene duplications. These genes have since been predominantly maintained in synteny in some species, and secondary losses explain the current distribution. We also found two interesting genes that were clearly derived from myosin genes, demonstrating that possible redundant or useless genes, instead of simply being lost, can serve as raw material for the evolution of new genes and functions.

## Introduction

Myosins are important eukaryotic molecular motor proteins that bind actin filaments and are dependent of ATP hydrolysis^1^. They are related to several molecular processes, such as muscle contraction in metazoans, cytokinesis, cell migration, intracellular transport of molecular cargoes and organelles, and host cell invasion of apicomplexan parasites^2,3^.

Most myosin proteins consist of an N-terminal motor domain (myosin head domain - PF00063) responsible for actin binding and ATP hydrolysis, a neck region containing one or more IQ motifs that bind calmodulin or other members of the EF-hand family of proteins, and a C-terminal tail responsible for cargo binding and/or dimerization that may contain distinct domains^4,5^.

In the last 20 years, a number of works have addressed the diversity, classification and evolution of myosin genes using different approaches and datasets, revealing that myosins are a large and diversified gene family^3,5–10^. Due to this diversity, their classification is not trivial work and there is still not a consensus from the last works. Two most recent myosin superfamily works, Odronitz and Kollmar^5^ and Sebé-Pedrós *et al*.^9^, used the phylogenetic relationship of myosin motor domains for classification. However, mainly due to taxon sampling differences, the authors defined a distinct number of classes (35^5^ or 31 classes^9^, respectively). Most studies included several taxa, such as early branching eukaryotes of Kinetoplastida class from Excavata.

Kinetoplastids are a widespread and diverse group of flagellated protozoans that are free-living or parasites and comprise five orders: Trypanosomatida, Eubodonida, Parabodonida, Neobodonida and Prokinetoplastida^11^. They contain a characteristic structure called kinetoplast, which is a complex mitochondrial DNA inside of a single mitochondrion^12^. Within kinetoplastids, the most studied family is the Trypanosomatidae, which is composed mainly of monoxenous parasite species that infect invertebrates and of dixenous species that can be pathogenic to plants, animals and/or humans^13^. Among these, *Trypanosoma cruzi*, *T*. *brucei* and *Leishmania spp*. are the causative agents of important neglected illnesses: Chagas disease, African sleeping sickness and leishmaniasis, respectively^14^.

Odronitz and Kollmar^5^ used several trypanosomatid genomes (*T*. *cruzi*, *T*. *brucei*, *T*. *vivax*, *T*. *congolense*, *L*. *major*, *L*. *infantum* and *L*. *braziliensis*) in their myosin phylogenetic tree. All of these species presented a Myo1 gene, representative of class I that is found in almost all organisms, and a Myo13 gene, classified as class XIII, a Kinetoplastida-specific class. Notably, *T*. *cruzi* has seven additional myosin genes (named MyoA, MyoB, MyoC, MyoD, MyoE, MyoF and MyoG) that were not classified, being considered “orphan” myosins since no orthologues were found in other species^5^. In Sebé-Pedrós *et al*.^9^, authors only included *L*. *major* sequences, and the trypanosomatid myosin classification remained the same.

With the sequencing of some *T*. *cruzi*-related species, we observed that *T*. *cruzi* “orphan” myosins are more widely distributed than previously thought, leading to questions about the evolution and function of these genes. Here, we present a deep evolutionary analysis of the myosin gene family from Trypanosomatidae, with exciting new data about expansion, diversification, loss and neofunctionalization of these genes in Kinetoplastida. Although the role of these genes remains to be addressed, this work is a starting point for functional studies.

## Results and Discussion

### Evolutionary analyses of myosin genes

For all myosins, we used the *T*. *cruzi* Dm28c amino acid sequences as query on BlastP searches and the main features of these genes are available in Table 1. We focus our main text on the evolutionary aspects, while a detailed result of protein domain searches of all sequences found in this work can be seen in Supplementary Table S1 together with a Supplementary Discussion. Supplementary Table S2 contains the information about the genome assemblies used in work and we provided all BlastP and synteny analysis results in the Supplementary Tables S3 to S22.

**Table 1:** Information of *T*. *cruzi* Dm28c myosin genes used as query in the BlastP searches.

Gene	Gene ID	Gene size (bp)	Protein size (aa)	Domains
Myo1	TCDM_07314	3,498	1,165	Myosin motor domain (cd01378/PF00063); IQ motif; Unconventional myosin tail domain (Myosin TH1 superfamily domain - cl26987); WW domain (cd00201); FYVE_like_SF (cd00065); coiled-coil region
Myo13	TCDM_05821	3,177	1,058	Myosin motor domain (MYSc_Myo13, cd14875); coiled-coil region; two tandem UBA superfamily domains (cl21463)
MyoA	TCDM_09957	3,012	1,003	Myosin motor domain (cd00124); IQ motif; two coiled-coil regions
MyoB	TCDM_07433	3,357	1,118	Myosin motor domain (cd00124); coiled-coil regions; IQ motif
MyoC	TCDM_02877	3,304	1,167	Myosin motor domain (cd00124); coiled-coil region
MyoD	TCDM_07686	3,678	1,225	Myosin motor domain (cd00124); IQ motif
MyoE	TCDM_07686	3,288	1,095	Myosin motor domain (cd00124); two IQ motifs
MyoF	TCDM_08875	4,446	1,481	Myosin motor domain (cd00124); IQ motif; two coiled-coil regions
MyoG	TCDM_02016	3,669	1,222	Myosin motor domain (cd00124); TPH (pfam13868)
MyoH-derived	TCDM_02145	3,594	1,197	Short region of class XIII myosin motor domain (cd14875); TPH (ERM superfamily - cl25742); Neuromodulin_N superfamily (cl26511); SMC_N superfamily (cl25732); two coiled-coil regions

**Figure 1:**
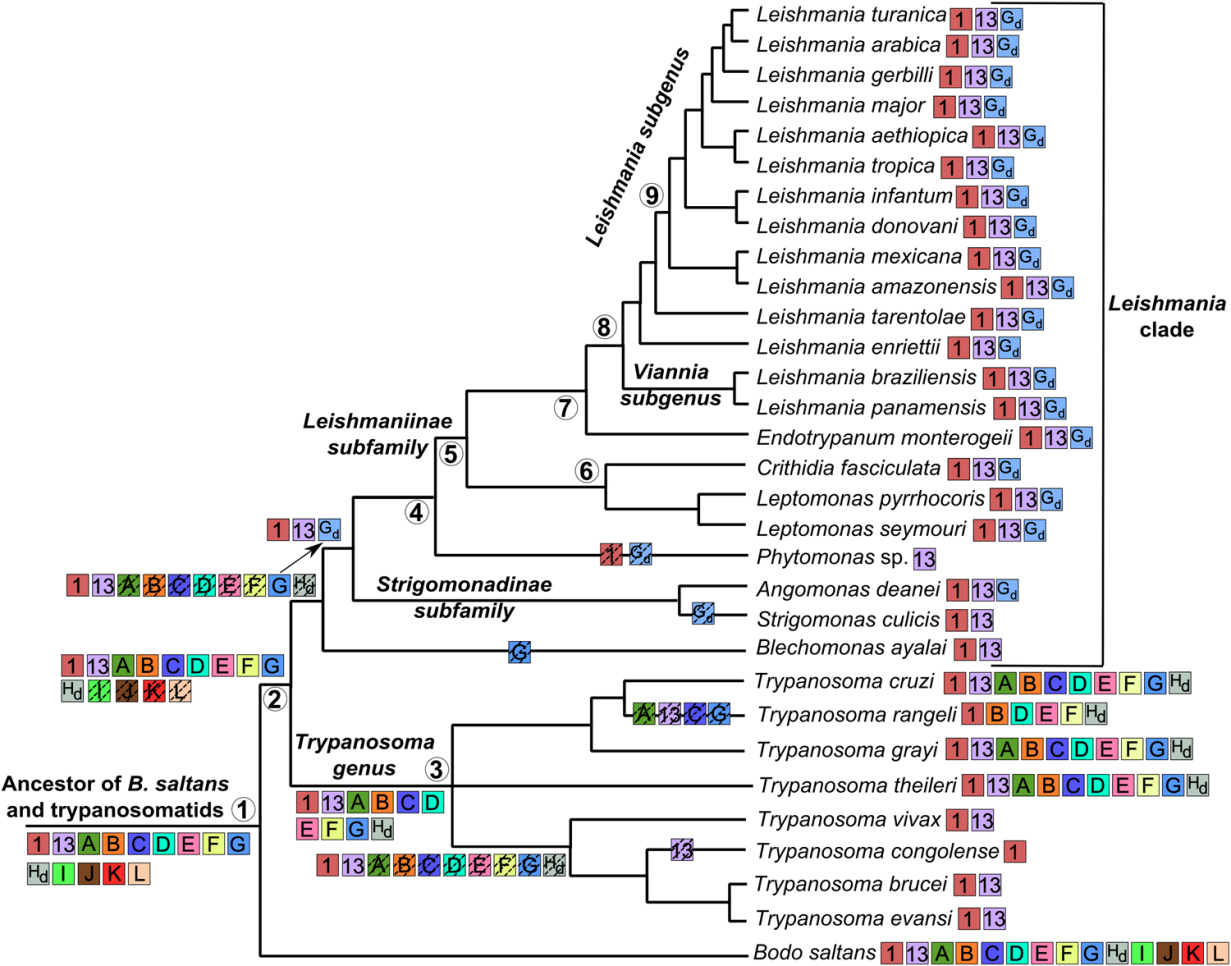
Schematic of phylogenetic relationships among different trypanosomatids employed in this study based on several works^15,42–48^. *Bodo saltans* was represented as outgroup. Coloured boxes represent the presence of different myosin genes. Hashed boxes represent possible gene loss events. Gd – MyoG-derived gene. Hd – MyoH-derived gene. Numbers in circles near some nodes are references to the divergence time estimates in mya (millions years ago), as inferred by Lukes *et al*.^15^: 1 – 463-695 mya; 2 – 231-283 mya; 3 – 96-105 mya; 4 – 118-170 mya; 5 – 52-96 mya; 6 – 30-63 mya; 7 – 31-65 mya; 8 – 25-54 mya; 9 – 9-23 mya. The branching is not drawn to scale.

To understand the evolution of myosin genes, we compared their phylogenies to the known trypanosomatid phylogenetic relationships (Fig. 1). Trypanosomatid phylogeny can be separated into two major clades, one comprising the *Trypanosoma* genus, and the other, which we named the *Leishmania* clade, that encompasses *Blechomonas ayalai*, *Phytomonas* sp. and the subfamilies Strigomonadinae and Leishmaniinae. The separation of these two groups is estimated at 231–283 million years ago (mya)^15^. *Bodo saltans*, a non-trypanosomatid free-living kinetoplastid also studied here, is clearly an early branch. All evolutionary scenarios presented here were hypothesized according to the available data and based on the most parsimonious picture. However, alternative explanations cannot be discarded, and the addition of new kinetoplastid species into the phylogeny could give a better view of the evolution of the myosin family.

### Myo1 protein is widely found in trypanosomatids

Using TritrypDB BlastP searches, we were able to find Myo1 orthologous genes in all analysed species. In addition, NCBI BlastP was performed to search for trypanosomatid sequences that are not available at TritrypDB, and we found Myo1 sequences in *T*. *theileri*, *Angomonas deanei*, *Strigomonas culicis* and in *B*. *saltans*. Some species (*T*. *grayi*, *T*. *rangeli*, *T*. *theileri* and *B*. *saltans*) presented several significant hits, indicating that these species have a wide repertoire of myosin genes, similar to *T*. *cruzi*. The wide distribution of Myo1 in Kinetoplastida is expected since this class has the widest taxonomic distribution (being absent only in Viridiplantae and Alveolata), probably by being the first myosin to have evolved^5^. Additionally, Myo1 genes were found in previous works that sampled trypanosomatid species^3,5,8,9^. We found a conserved synteny (the same gene order in a chromosomal segment between species) for Myo1 genes in almost all species.

In general, Myo1 sequences presented conserved structure and motifs/domains composition, having a myosin motor domain in the N-terminal region, an IQ motif and an unconventional myosin tail domain (Myosin TH1 superfamily domain) with a WW and a FYVE_like_SF domain. This protein also contains a putative C-terminal coiled-coil forming region. *T*. *rangeli* presents a truncated protein; however, we cannot discard that it is an artefact of genome sequencing/assembly.

Interestingly, *Phytomonas sp*. (isolates Hart1 and EM1) did not present any hit that corresponded to a Myo1 orthologue. It is not clear if this absence is due to genome assembly issues. Nonetheless, the absence in two independent genome assemblies supports the idea of gene loss. This last hypothesis is corroborated by the whole-genome analysis that indicates a minimized gene repertoire in *Phytomonas* EM1 and HART1 genomes^16^.

**Figure 2:**
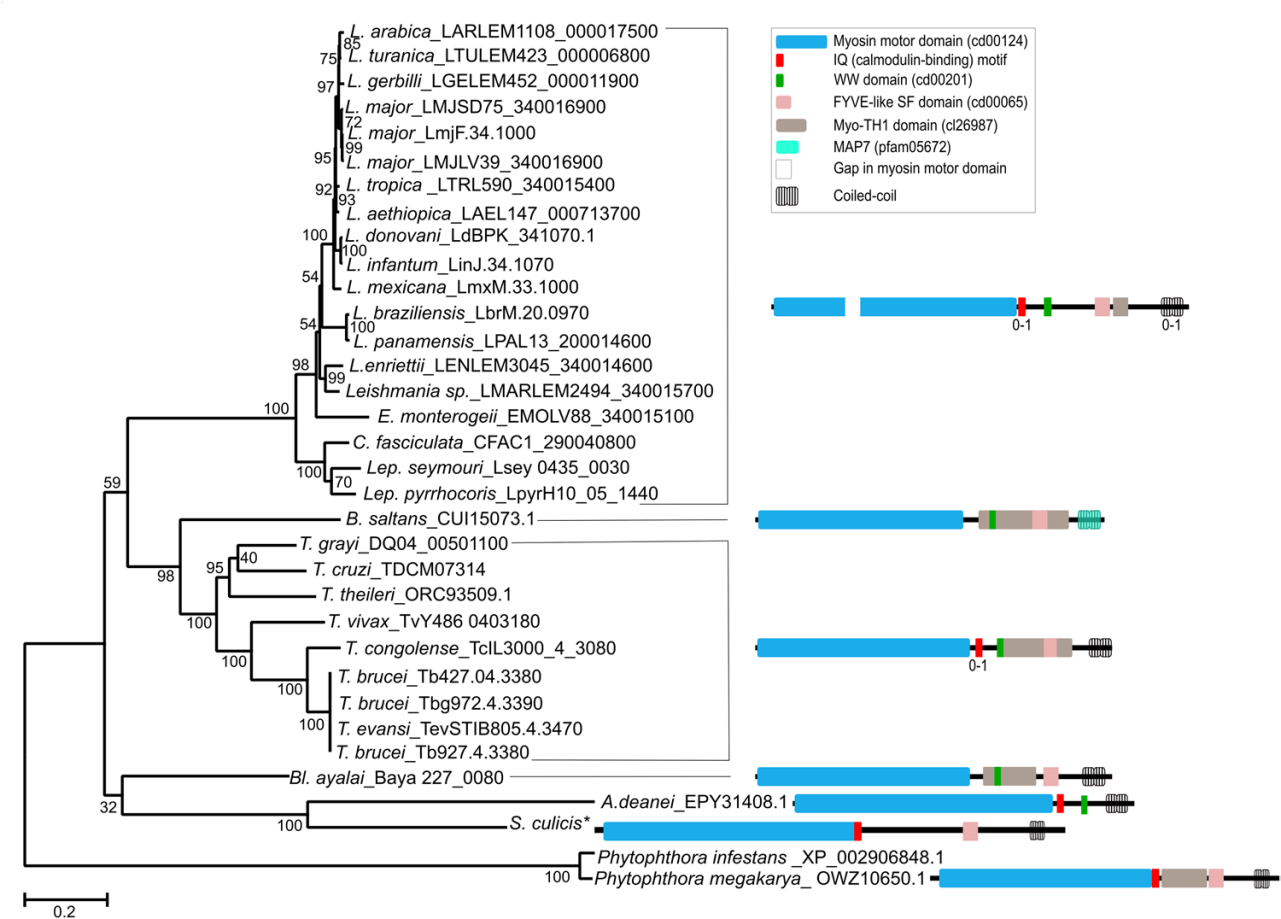
Phylogenetic tree of kinetoplastid Class I myosin proteins. The evolutionary history was inferred by using the maximum likelihood method based on the JTT + G (4 categories) model. Bootstrap values from 1,000 replicates are shown next to the branches. The tree is drawn to scale, with branch lengths measured in the number of substitutions per site. The analysis involved 34 amino acid sequences. There were 1,185 positions in the final dataset. *Phytophthora* genus related myosins were used to root the tree. Sequences are identified by the name of species and the GenBank or TritrypDB accession number. Asterisk represents the *S*. *culicis* Myo1 sequence obtained from the *S*. *culicis* TCC012E genome, contig coordinates AUXH01000328.1:350-4390. *T*. *rangeli* sequence (TRSC58_03135) was not included in the phylogeny because it is very short. Protein domain architectures of main sequences are shown.

Excluding the kinetoplastids from NCBI BlastP, the best hits corresponded to sequences from the *Phytophthora* genus (~87% extension, 41% identity). In the Odronitz and Kollmar (2007) myosin phylogeny, trypanosomatid Myo1 sequences also grouped, with high support, with myosin from *Phytophthora* spp^5^. Considering this close relation, we used two *Phytophthora* sequences to root the Kinetoplastida Myo1 tree (Fig. 2). Myo1 phylogeny presents low support for the basal nodes and some incongruities in relation to the species phylogeny. *B*. *saltans* is positioned closer to the *Trypanosoma* genus instead of being a basal branch. One hypothesis for this incongruity could be ancestral polymorphism followed by independent lineage sorting. However, the conserved synteny suggests the same copy gene was maintained from the ancestor. The clade containing *Bl*. *ayalai*, *S*. *culicis* and *A*. *deanei* is misplaced, but the bootstrap value is very low. The wrong positioning could be explained by long branch attraction, since the long branches of outgroups can frequently “attract” long branches of species to the base of the tree^17^. We can observe by the short branches among species that Myo1 genes from Leishmaniinae subfamily members are highly conserved; nevertheless, there was likely an accelerated evolutionary rate in the ancestor species that is represented by the long branch connecting this group.

In general, we can postulate that the Myo1 gene was present in the ancestor of kinetoplastids as a single copy gene and was conserved in almost all species, possibly due to an essential function, although little is known about the role of this gene in these organisms. In *T*. *brucei* bloodstream forms, Myo1 protein partially colocalizes with elements of the endocytic pathway and compartments containing internalized cargo, indicating its involvement in this pathway, being lethal in knockdown cells. However, in procyclic forms, the knockdown has no obvious effect on growth and morphology^18^. In contrast, in *L*. *donovani*, the protein Myo1 was not found by antibody recognition in either procyclic or amastigote forms, indicating that this gene is not expressed in this parasite^19^. Despite this finding, it is not reasonable, based on our phylogenetic analysis, that this myosin has remained conserved along the evolution without functional activity. It is possible that this gene is expressed in intermediate forms of the parasite’s cycle or expressed in such low amounts that it was not detected by the authors. In addition, we cannot presume that the function of Myo1 genes is the same in all trypanosomatids, mainly because some species demonstrate high divergence in the protein sequence, such as *A*. *deanei* and *S*. *culicis*. Moreover, the function of myosin genes is expected to differ among species with reduced or expanded repertoires of myosin genes. A deeper discussion concerning Myo1 domains can be found in the Supplementary Discussion.

### Myo13 is a widely distributed myosin in kinetoplastids, and *T*. *cruzi* myosins MyoA, MyoB, MyoC, MyoD, MyoE and MyoF can no longer be considered “orphans”

**Figure 3:**
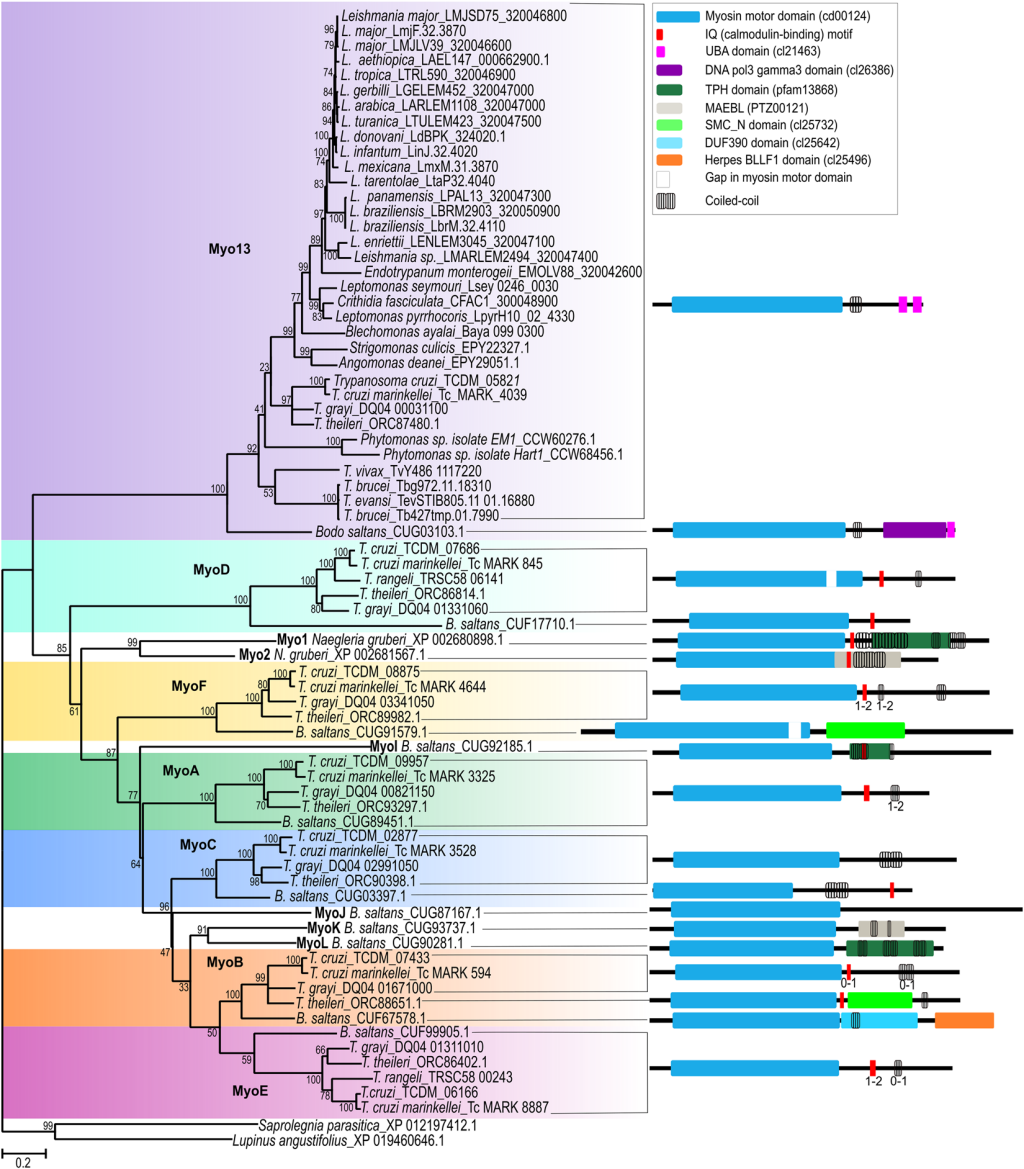
Phylogenetic tree of class XIII myosin proteins. The evolutionary history was inferred using the maximum likelihood method based on the LG + G (4 categories) + I model. Bootstrap values from 1,000 replicates are shown next to the branches. The tree is drawn to scale, with branch lengths measured in the number of substitutions per site. The analysis involved 78 amino acid sequences. There were 621 positions of head domain in the final dataset. *Saprolegnia parasitica* (XP 012197412.1) and *Lupinus angustifolius* (XP 019460646.1) myosins were used to root the tree. Sequences are identified by the name of species and the GenBank or TritrypDB accession number. Protein domain architectures of main sequences are shown.

In the myosin global phylogeny presented by Odronitz and Kollmar (2007), it is clear that the *T*. *cruzi* orphan myosins MyoA, MyoB, MyoC, MyoD, MyoE and MyoF are related to Myo13 since they grouped together with high support^5^. We found orthologous sequences to these genes in some other species (Fig. 3).

The Myo13 gene has orthologues in almost all kinetoplastid species that were analysed and in conserved synteny. Sequences from most species are of similar size and domains composition, containing the myosin motor domain, followed by a coiled-coil region and two tandem UBA superfamily domains. For some species, additional less-significant domains were found. The Myo13 gene is absent in *T*. *congolense*, and no remnants of this gene were found in the syntenic region. It also appears absent in *T*. *rangeli* and the evaluation of the syntenic region for this species was not possible for Myo13 and all other genes, as the genome contains very small contigs.

MyoA gene was found in syntenic conserved regions relative to *T*. *cruzi* only in *T*. *grayi* and *T*. *theileri*. Unexpectedly, MyoA is also present in the distantly related species *B*. *saltans*, also in synteny. Most predicted proteins have a myosin motor domain, an IQ motif and two coiled-coil regions. No signal or remnants of the MyoA gene were found in the syntenic region from the other species analysed.

MyoB was also found in conserved synteny with the *T*. *cruzi* gene in *T*. *grayi*, *T*. *theileri* and *B*. *saltans*. The MyoB proteins contain the myosin head domain and can have a coiled-coil region and an IQ motif; other additional domains were also predicted for some species. *T*. *rangeli* has a truncated MyoB gene that could be due to poor genome assembly.

Interestingly, *B*. *saltans* presented four additional hits (CUG92185.1, CUG87167.1, CUG93737.1, CUG90281.1) in which reciprocal BlastP against *T*. *cruzi* showed MyoB as the best hit. Since these proteins do not cluster with other myosin clades in the phylogeny, they can be considered new myosin genes. The adopted nomenclature for these genes followed the nomenclature of kinetoplastid myosin genes (MyoI, CUG92185.1; MyoJ, CUG87167.1; MyoK, CUG93737.1 and MyoL, CUG90281.1; since MyoH was reserved for another gene that is discussed in a next section). These *B*. *saltans* proteins present the myosin head domain and two or three coiled-coil regions, and the MyoI protein has a predicted IQ motif. Overlapping the coiled-coil regions, some distinct and non-related domains were predicted with less significance.

The MyoC gene also has orthologues only in *T*. *grayi*, *T*. *theileri* and *B*. *saltans* in syntenic conserved regions compared to the *T*. *cruzi* gene. The predicted proteins have similar structures, containing a myosin motor domain and a coiled-coil region. The IQ motif was only predicted in *B*. *saltans* MyoC. No remnants of MyoC gene were found in the other species.

The MyoD gene was also found in *T*. *rangeli*, *T*. *grayi*, *T*. *theileri* and *B*. *saltans*. *T*. *grayi* and *T*. *theileri* MyoD genes are in syntenic conserved locations in relation to the *T*. *cruzi* gene, while the *B*. *saltans* gene location is not conserved. No remnants of MyoD were found in the syntenic regions from other species. *B*. *saltans* MyoD protein is shorter than the sequences from the other species, with a gap in the myosin head domain. IQ motifs and coiled-coil regions are predicted for some sequences.

*T*. *cruzi* MyoE orthologous genes were found in *T*. *rangeli*, *T*. *grayi*, *T*. *theileri* and *B*. *saltans*. Except for *T*. *rangeli*, the gene is in conserved synteny in all species. The predicted proteins have similar structure, containing a myosin motor domain and two IQ motifs. Additional coiled-coil regions are predicted in *T*. *rangeli* and *T*. *theileri*.

The MyoF gene also has orthologues in *T*. *grayi*, *T*. *theileri* and *B*. *saltans* in syntenic conserved regions compared to the *T*. *cruzi* gene. The proteins contain a myosin motor domain, one or two IQ motifs and two or three coiled-coil domains. A C-terminal truncated version of MyoF gene is also found in *T*. *rangeli*, encoding only part of the myosin head domain.

Excluding the kinetoplastids from NCBI BlastP searches and using all the queries (Myo13 and MyoA to MyoF), the two best hits correspond to sequences from the amoeba *Naegleria gruberi (Excavata, Heterolobosea class)* (named by us Myo1: XP_002680898.1 and Myo2: XP_002681567.1). In the myosin phylogeny presented by Odronitz and Kollmar^5^, these two *N*. *gruberi* genes were grouped inside of this kinetoplastid myosin clade. The following hits corresponded to myosin genes from different organisms, with alignments ranging from 40–70% of extension with approximately 35% identity. Due to the conservation of the myosin head domain and the huge number of myosin genes available at NCBI, numerous hits can be found with similar e-values. Some examples of hits in non-kinetoplastid species were cited in the Supplementary BlastP tables. However, except for the two *N*. *gruberi* myosins, the other sequences can be considered outgroup sequences since they do not locate inside the kinetoplastid myosin clade (Supplementary Figure S1).

To have a better view of evolution and relationship among all these genes, we inferred a phylogenetic tree based on the most conserved regions of the myosin head domain (621 positions), which is shown in Fig. 3. Two non-kinetoplastid sequences retrieved from BLAST in the other species were used as outgroup. The phylogeny shows significant support for most of the important clades, and the relationships among myosin groups are congruent to those presented by Odronitz and Kollmar^5^. Myo13 was the first clade to branch, followed by the MyoD clade, *Naegleria* myosins, MyoF clade, *B*. *saltans* MyoI, MyoA clade, MyoC clade, *B*. *saltans* MyoJ, MyoK and MyoL, and finally the MyoB and MyoE clades. This topology reflects the pattern of gene duplication that will be discussed later.

Analysing the relationships inside Myo groups, we observed some incongruities in the Myo13 clade in relation to the species tree (Fig. 1), such as wrong positioning of some *Leishmania* species and *Bl*. *ayalai*, the clustering of *Lep*. *pyrrhocoris* with *C*. *fasciculata*, and in the base of the tree, *Trypanosoma* species were separated into two clades interspersed by *Phytomonas* sp. sequences. Different rates of evolution among sequences or long-branch attraction could explain these incongruities. The relationships found among sequences from MyoA to MyoF clades are in congruence with the species tree (except for the absence of sequences in some species), with *B*. *saltans* sequences at the base of clades, but the positioning of *T*. *theileri* changes in some groups. The positioning of this species in the *Trypanosoma* genus is not clear^20–22^.

Class XIII myosins were described earlier as an exclusive kinetoplastid class comprising only Myo13, being the myosins MyoA to MyoF from *T*. *cruzi* considered “orphans”^5^. We found that MyoA, MyoB, MyoC, MyoD, MyoE and MyoF have orthologous genes in other kinetoplastid species that were not previously analysed, grouping together with Myo13 and, therefore, they were considered as Class XIII myosins (Fig. 3). In addition, we found that *B*. *saltans* has an even greater and unique repertoire of class XIII myosin genes, since the four extra genes (MyoI to MyoL) do not cluster with the other myosin clades and were positioned inside the phylogeny, before the last branching, suggesting that these sequences were also present in the ancestor species. Despite the clear relationship between MyoK and MyoL, their divergence suggests ancient duplication.

The positioning of *B*. *saltans* Myo13, MyoD, MyoF, MyoA, MyoC, Myo B and MyoE in the base of clades grouping with high support indicates that all these genes were present in the common ancestor of trypanosomatids and *B*. *saltans*, being transmitted vertically. Horizontal transfer among species could be discarded due to the synteny conservation. *Naegleria* myosin sequences do not group with any kinetoplastid myosin group, but they are clearly related, branching after MyoD separation. Independent secondary loss events should be postulated to explain the pattern of gene absence. Myo13 was lost in *T*. *congolense* and *T*. *rangeli*; the other class XIII myosin genes present very similar patterns of loss in the ancestor of the *T*. *vivax/T*.*congolense/T*. *brucei/T*. *evansi* lineage and in the ancestor of the *Leishmania* clade. The absence of any remnants of these genes in the syntenic regions supports the idea of long-term loss events. If we consider that the *T*. *rangeli* genome is complete, the MyoA and MyoC genes were also lost in this species.

The primary origin of Class XIII myosin is not clear since it lacks a significant phylogenetic relationship to other myosin genes. It could be a result of a very ancient horizontal transfer event from an unidentified source to an ancient ancestor species. Alternatively, the ancestor Class XIII myosin may have originated by mutations of a redundant duplicated myosin gene. The addition of myosin sequences from new species that sample both Kinetoplastida and Heterolobosea will certainly help to clarify this point.

Clues of class XIII myosin functions come mainly from *L*. *major* Myo13 studies, where it seems to be related to the assembly of flagellum^19,23^. Myo13 protein is expressed both in promastigote and amastigote parasite forms, besides its increased expression in promastigotes, where it localizes preferentially at the proximal region of the flagellum and colocalizes with paraflagellar rod (PFR) proteins^23^. In a later work, Katta *et al*.^19^ showed that *L*. *major* Myo13 associates with actin and is involved in intraflagellar transport (IFT), since knockout cells presented reduced flagellar length and absence of PFR. LmMyo13 gene appears to be essential for parasite survival whereas the double mutant generated Myo13 ploidy^19^. Additionally, the IQ motif predicted in Myo13 genes was analysed in *Leishmania* and proven to be functional, with the binding of calmodulin regulating dimerization, motility and lipid binding of this molecular motor^24,25^. However, in *T*. *brucei* there is no indication of Myo13 function, since its knockdown in bloodstream forms had no effect on vesicle traffic or growth^18^.

### MyoG protein has evolved to a new protein in Leishmaniinae subfamily

MyoG was considered an “orphan” myosin since it was previously found only in *T*. *cruzi*^5^. Using TritrypDB and NCBI BlastP searches, we found orthologues in the closely related species *T*. *grayi* and *T*. *theileri* and in *B*. *saltans*. Analysis of *T*. *grayi* and *T*. *theileri* genomes indicated that the gene is in a syntenic conserved location in relation to the *T*. *cruzi* MyoG. The products of these genes present similar patterns of domains as *T*. *cruzi* MyoG.

**Figure 4:**
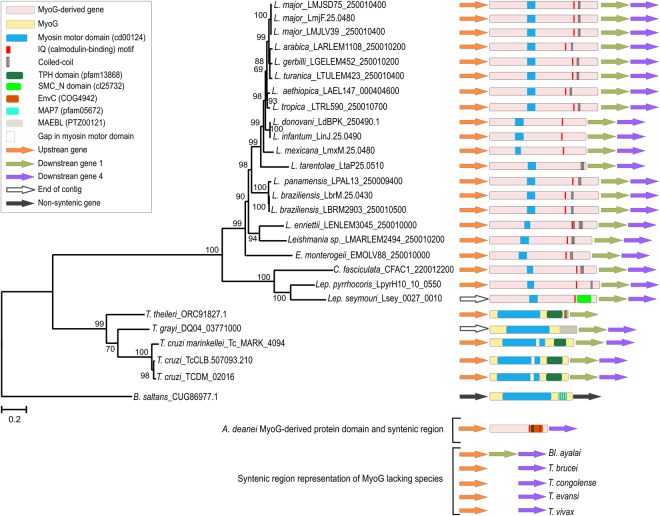
Phylogenetic tree of kinetoplastid MyoG and MyoG-derived proteins. The evolutionary history was inferred by using the maximum likelihood method based on the JTT + G (4 categories) + I model. Bootstrap values from 1,000 replicates are shown next to the branches. The tree is drawn to scale, with branch lengths measured in the number of substitutions per site. The analysis involved 28 amino acid sequences. There were 568 positions in the final dataset. *B*. *saltans* MyoG was used to root the tree. Sequences are identified by the name of species and the GenBank or TritrypDB accession number. Protein domain architectures of sequences are shown together with a representation of their genomic context. Due to the high divergence, the *A*. *deanei* MyoG-derived protein was not included in the phylogeny.

For *Leishmania* and *Leptomonas* species, *C*. *fasciculata*, *E*. *monterogeii* and *A*. *deanei*, the two best BlastP hits represent Myo13 and Myo1 genes. Interestingly, a third hit with a higher e-value was also found in these species. These hits correspond to genes annotated as “conserved hypothetical protein”. We then identify that these genes are in syntenic locations in relation to *T*. *cruzi* MyoG in all these species (Fig. 4). Their protein products have similarity to MyoG at the C-terminal region (last 400 aa) and in a short region (only 130 aa) of the motor domain. For the *A*. *deanei* sequence, however, the similarity is limited to a very short region at the C-terminal portion (approximately 240 aa) and there is no remnants of the myosin motor domain. Clearly, these sequences represent a MyoG-derived gene that had accumulated numerous mutations, almost losing the motor domain. The low conservation of the myosin head domain in MyoG-derived proteins likely prevented the authors from finding it in *L*. *major* in the previous works^3,5,8,9^. Additionally, the MyoG-derived proteins have a predicted coiled-coil region and an IQ motif.

*Bl*. *ayalai*, *T*. *brucei*, *T*. *evansi*, *T*. *vivax*, *S*. *culicis* and *Phytomonas sp*. only presented BlastP hits corresponding to Myo13 and Myo1, and *T*. *congolense* only to Myo1. To evaluate the possible loss event of MyoG in these species, we analysed the syntenic regions where the gene should be located and we found no remnants of MyoG in these species. Moreover, *T*. *brucei*, *T*. *evansi* and *T*. *congolense* also lost the downstream gene. *T*. *vivax* has a large region of missing data in this location; nonetheless, it is plausible that the loss of the genes occurred in the ancestor of these species. *T*. *rangeli* apparently lost the MyoG gene.

To better understand the evolution of MyoG and MyoG-derived genes, their conserved amino acid regions (568 positions) were used to infer a phylogeny (Fig. 4). The *A*. *deanei* MyoG-derived protein was not included due the high divergence that made the alignment non-confident. The MyoG tree shows high supported nodes, and its topology largely reflects the relationships of species (Fig. 1). *B*. *saltans* was the first species to branch, followed by the split of the *Trypanosoma* genus and *Leishmania* clade.

Excluding the kinetoplastids in the NCBI BlastP searches, the best hits were from several distinct organisms, including fungi, insects, fishes and others, presenting e-values around 1E-60 (approximately 30% identity covering 50–70% of query). Reciprocal BlastP showed that other myosins are more similar to these sequences than MyoG. This finding is in accordance with the data of Odronitz and Kollmar^5^ since in the myosin phylogeny presented by these authors the *T*. *cruzi* MyoG does not group with good support to any other myosin. Thus, the kinetoplastid MyoG genes comprise a new class that we named Class XXXVI, a class that was not used in the recent works of myosin classification^5,9^.

The origin of MyoG in Kinetoplastida is unknown and could be a result of a very ancient horizontal transfer event or originated by mutations of a redundant duplicated myosin gene. In this case, we need to suppose that the gene underwent extremely high rates of mutations that make it impossible to track the relationship with its precursor. After the primary origin, it is presumed that the MyoG gene was vertically transmitted throughout trypanosomatids evolution. Although there is a discontinuity in the gene presence among species and absence of a conserved synteny region in *B*. *saltans*, the divergence among sequences does not support a horizontal transfer hypothesis. Nevertheless, considering the picture that we have now, we need to suggest several events of MyoG loss during species evolution: in the ancestor of *T*. *brucei*, *T*. *evansi* and *T*. *congolense*, in *T*. *rangeli*, in *Bl*. *ayalai*, in *Phytomonas sp*. and in *S*. *culicis*. The function of MyoG and MyoG-derived genes remains to be studied.

### *MyoH*-derived: a possible myosin-derived gene

In Myo13 BlastP searches, we also found a less significant hit (e-value > E-10) that caught our attention in *T*. *cruzi*, *T*. *rangeli* and *T*. *grayi*. In *T*. *cruzi*, this hit corresponded to the gene TCDM_02145, annotated as “hypothetical protein”. It has a very short conserved region (only 144 aa) of the myosin domain (MYSc_Myo13, cd14875). In the C-terminal portion of the protein, it contains a predicted TPH domain (pfam13868). As for MyoG, overlapping this domain there is a Neuromodulin_N superfamily (cl26511), a SMC_N superfamily (cl25732) and an ERM superfamily (cl25742) domains. Despite the similarity of domain composition between MyoG and this protein, their alignment on BlastP-2-sequences was not significant (e-value 0.087), suggesting they are not clearly related. However, the similarity between *T*. *cruzi* TCDM_02145 and Myo13 proteins was significant (e-value 3E-10 on BlastP-2-sequences), but restricted to the short region of myosin head domain (25% identity and 46% similarity over 145 aa). The similarity was more evident when we compared the *T*. *rangeli* protein (TRSC58_00453) with the Myo13 protein from *L*. *panamensis* (LPAL13_3200047300), since we observed a good alignment in the first 430 aa (24% identity, 39% similarity, e-value 3E-10 on BlastP-2-sequences).

Due to the presence of a remnant of myosin head domain and the similarity with Myo13, we conclude that this gene had derived from an ancient class XIII myosin gene. However, unlike what occurred to MyoG-derived genes, we could not find any copy with an intact myosin head domain. Following the nomenclature of *T*. *cruzi* myosin genes, we assumed that the mother gene was an ancestral MyoH gene; thus, we called it MyoH-derived gene. We then conducted BlastP searches using the *T*. *cruzi* MyoH-derived protein as the query, and the gene was also found in *T*. *theileri* and *B*. *saltans* and synteny conservation was found for all MyoH-derived carrier species. When we excluded the kinetoplastids from NCBI BlastP searches, only one hit was found with low significance matching a non-myosin protein from *Branchiostoma belcher* (E = 0.89; XP_019640798.1). Since MyoH-derived proteins accumulated higher divergence in relation to Myo13 and the other myosins (that could be considered outgroups), the alignment of sequences were very poor with low confidence; thus, we did not construct a MyoH-derived phylogenetic tree. The conversion of MyoH gene to MyoH-derived gene must have occurred in the ancestor of *B*. *saltans* and trypanosomatids. Interestingly, the MyoH-derived gene has the same pattern of loss as other class XIII myosins.

### Kinetoplastid myosins expansion, neofunctionalization and secondary losses

Several molecular mechanisms are known to be involved in the creation of new gene structures, such as exon shuffling, gene duplication, retroposition, horizontal transfer, domestication of mobile elements, gene fusion/fission and *de novo* origination^26^. The class XIII myosins are clear results of gene duplications and at least 13 very ancient events need to be postulated to explain their diversification, showing that the expansion of this class had an impact on kinetoplastid evolution.

**Figure 5:**
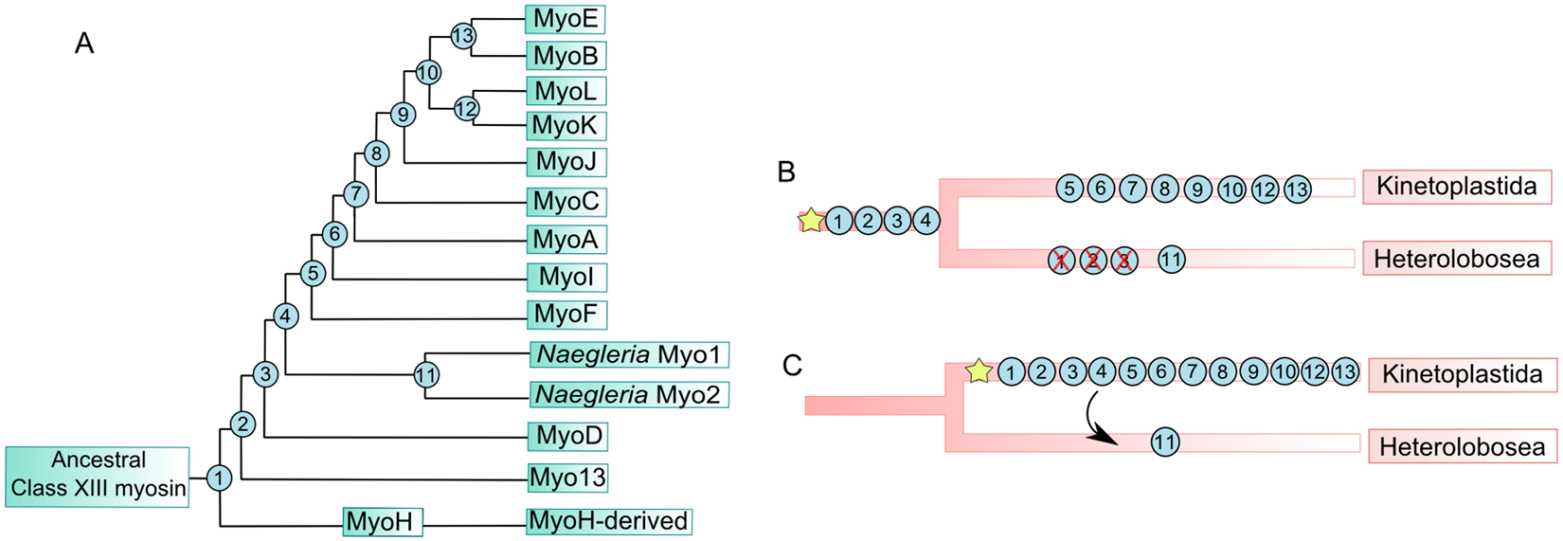
Possible evolutionary scenario of class XIII myosin genes duplications. (**A**) Representation of 13 putative duplication events (numbered circles) from an ancestral class XIII myosin gene with an unknown primary origin. The order of duplications from 2 to 10 is supported by the phylogeny, while the others are not clear. (**B**) Possible scenario of evolution where the origin of the first class XIII myosin (yellow star) and duplications 1, 2, 3 and 4 occurred before separation of Kinetoplastida and Herelobosea classes. In this case, some genes were lost in Herelobosea (circles filled with red “X”). (**C**) Possible scenario of evolution where the origin of the first class XIII myosin (yellow star) and almost all duplication events occurred after separation of the Kinetoplastida and Herelobosea classes. The presence of two copies of this myosin class in *Naegleria* can be explained by an ancient horizontal transfer event (black arrow) from a kinetoplastid to a Heterolobosea member followed by an event of duplication.

A possible scenario of gene duplications is postulated in Fig. 5A. The first duplication events (1 and 2) probably led to the MyoH and Myo13 genes, since they are related and Myo13 is the first clade to branch in the phylogeny. Those are followed by the duplications that generated the genes MyoD (3), the *Naegleria* myosin ancestor gene (4), MyoF (5), MyoI (6), MyoA (7), MyoC (8), MyoJ (9) and finally the duplication generating the ancestor of MyoK and MyoL and the ancestor of MyoE and MyoB (10). The order of duplications 11, 12 and 13 are hard to determine.

The positioning of *Naegleria* sequences in the phylogeny indicates that duplication events until this point must have occurred in the ancestor of Kinetoplastida and *Naegleria* species, as shown in Fig. 5B. In this scenario, Heterolobosea species have lost previously duplicated genes. Alternatively, to explain the phylogeny, all duplication events could have occurred in the ancestor of Kinetoplastida, but after its separation from the ancestor of Heterolobosea (Fig. 5C). Then, an ancient horizontal transfer event of a Class XIII myosin from a kinetoplastid to a *Naegleria* ancestor could explain this relationship.

Duplicated genes could have different fates, such as pseudogenization, conservation of function, subfunctionalization or neofunctionalization^27,28^. As the gene duplication generates functional redundancy, one of the copies is free to change, and the accumulation of mutations may transform the gene in a pseudogene that can be deleted after a long time^28^. We were not able to find any clear case of myosin pseudogene, corroborating the idea that the identified myosin gene deletions occurred long ago. Duplicated genes can otherwise be conserved with the same function, maintained by concert evolution or purifying selection, if there is an advantage in producing extra amounts of proteins or RNA products^27,28^. This seems not be the case for kinetoplastid myosins, since they present significant divergence in amino acid sequence and structure. Except for a few cases, the conservation of function is unlikely to be maintained.

The subfunctionalization occurs when each daughter gene adopts part of the functions of the parental gene^28,29^. This could be the case for class XIII myosins. Due the vast repertoire found in some species, it is possible to suggest that the original function of ancestral myosin was distributed to the derived genes. However, this hypothesis should be accompanied by a reversion of this scenario, since only one class XIII gene was maintained in several species.

Alternatively, it is possible that those myosin gene duplications gave rise to novel functions, a process called neofunctionalization, and in many cases, a related function evolves after gene duplication instead of a completely new function^28^. The conservation of the myosin head domain in most cases suggests that the function was partially conserved, and the diversified C-terminal tails may indicate specializations for different cargos/localizations. In this context, the high divergence among class XIII myosin sequences is expected since the evolutionary rate after duplication is predicted to be accelerated because of positive selection and functional diversification^30^.

On the other hand, there are several examples of entirely new function created by gene duplication^28^. It is reasonable that MyoH-derived genes have a non-myosin function, since the key domain conferring the properties of actin binding and ATP hydrolysis was almost lost. Additionally, although the MyoG is not a direct product of gene duplication, the probable redundancy of functions provided by the other myosin genes allowed the MyoG gene to accumulate mutations and, therefore, MyoG-derived genes also likely play a non-myosin function.

Our data shows that the ancestor of *B*. *saltans* and trypanosomatids had all class XIII myosins along with Myo1, MyoG and the MyoH-derived gene (Fig. 1). Most genes were found in the free-living kinetoplastid *B*. *saltans* and in *T*. *cruzi* and related Stercoraria species indicating that these genes were preserved in these species for more than 500 million years. Additionally, almost all myosin genes were found in conserved synteny, contrary to what is generally found, since the conservation of gene order between *B*. *saltans* and trypanosomatids is low, making up approximately 9% of the genes^31^. This finding raises the question of what is the function of these genes and why they were conserved only in those species.

The pattern of gene loss also seen for the myosin genes during the trypanosomatids evolutions is in agreement to a recent work that shows that trypanosomatids present streamlined genomes consistent with loss of redundancy that could be associated with the alteration from an ancestral free-living state inhabiting diverse environments to an obligate parasite that explores relatively constant host environments^31^. The authors also observed several genes that are unique to one or more parasites, indicating that gene gain also has a significant role in the origin of parasitism, likely via the rapid evolution of multi-copy gene families. Thus, we can predict that other redundant or useless genes, such as MyoG and MyoH, could have evolved new functions, and, rather than simply being lost, they can serve as a raw material for the evolution of new genes.

## Conclusions

In this work, we present the largest and most comprehensive evolutionary analysis of Kinetoplastida myosin genes, identifying interesting aspects of this family evolution such as its expansion by gene duplication events, the potential of myosin family as a source of new genes and the extensive gene loss of several members in a great number of trypanosomatid species. This work is an important beginning for future functional studies to comprehend the diverse roles of myosin proteins in Kinetoplastida species.

## Methods

Myosin amino acid sequences from the *T*. *cruzi* Dm28c strain were used as query on BlastP searches^32^ against genomes available at TritrypDB^33^ up to July 2017. Information about genomes is available in Supplementary Table S2. BlastP searches against non-redundant protein sequences (nr) from GenBank^32,34^ were also conducted to determine group sequences and some kinetoplastid sequences that are not available at TritrypDB. Since the motor domain is a well-conserved domain of a diversified protein family, BlastP searches provide several significant hits (e-value cutoff of E-10). Thus, sequences were considered orthologues when genes were in the same genomic context (conserved syntenic location) and/or if the query sequences appeared as the first hit on a reciprocal BlastP.

Synteny conservation was evaluated using the Genome Browser tool for species available at TritrypDB. For those available at NCBI (Supplementary Table S2), genome sequences were downloaded and submitted to local TBlastN^32^ using as query the myosin amino acid sequences or the amino acid sequences of upstream and downstream genes. The TcruziDm28cPB1 genome assembly (GCA_002219105.1) was also used for some specific gene searches. A Perl programming code was used to retrieve desired sequences. If the myosin genes were not present/annotated, the syntenic regions where the genes were supposed to be present were analysed by BlastX against the annotated proteins (e-value cutoff E-5) searching for non-annotated ORFs or remnants of the genes.

CD-search on NCBI Conserved Domain Database (CDD)^35^, SMART (Single Modular Architecture Research Tool)^36^ and ELM (The Eukaryotic Linear Motif resource)^37^ were used to find protein domains and motifs using default parameters.

Amino acid alignments were obtained by PSI-Coffee^38^. Alignments were visualized and edited using the Genedoc 2.7 package^39^. To avoid loss of phylogenetic signal, phylogenies from the three groups were constructed separately. For Myo1 and MyoG, well-aligned regions from entire proteins were selected based on PSI-Coffee alignment scores. For class XIII myosins, well-aligned regions were also selected but restricted to the myosin head domain. Phylogenies for each protein group were constructed by maximum likelihood (ML) method, using the amino acid substitution model indicated by the Model Selection. Reliability of branches was accessed by bootstrap test with 1,000 replicates. Phylogenetic analyses were implemented in MEGA 7.0 software^40^.

To understand the evolution of myosin genes, we compared their phylogenies to the known trypanosomatid phylogenetic relationships. Thus, Fig. 1 represents a consensus phylogenetic tree of trypanosomatids modified from Ludwig and Krieger^41^ to encompass the species used in this work and is based on well-supported trees from several studies. Positioning of *T*. *theileri* is inconclusive from works available up to now^20–22^ and is represented as a polytomy. For evolutionary inferences, we adopted the most parsimonious assumptions; however, we did not discard alternative hypotheses and/or further updates with studies on additional species.

## Electronic supplementary material

Dataset 1

Supplementary information
