# Supplementary material for: Evolutionary analyses of myosin genes in trypanosomatids show a history of expansion, secondary losses and neofunctionalization
Source: Sci Rep. 2018 Jan 22;8:1376. doi: 10.1038/s41598-017-18865-y (PMC5778035; doi:10.1038/s41598-017-18865-y)
Supplement: Supplementary file 2 — Supplementary information [file 41598_2017_18865_MOESM2_ESM.pdf]

Supplementary information

Evolutionary analyses of myosin genes in trypanosomatids show a history of expansion, secondary losses and neofunctionalization

Denise Andréa Silva de Souza[1,2], Daniela Parada Pavoni[1,2], Marco Aurélio Krieger[1,2,3],* Adriana Ludwig[1,3*]

[1] – Laboratório de Genômica Funcional, Instituto Carlos Chagas- ICC/Fiocruz-PR, Curitiba, 81350-010, Brazil;

[2] – Programa de Pós-graduação em Biociências e Biotecnologia - ICC/Fiocruz-PR, Curitiba, 81350-010, Brazil;

[3] – Instituto de Biologia Molecular do Paraná, Curitiba, 81350-010, Brazil

* Corresponding Author:

adriludwig@gmail.com; adriana.ludwig@pq.cnpq.br; mkrieger@fiocruz.br;

Supplementary Discussion

Class I myosin functions and domains

Trypanosoma cruzi Myo1 gene (TCDM_07314) has 3,498 base pairs (bp) encoding a 1,165-amino acid (aa) protein. The myosin motor domain (class I myosin: cd01378/PF00063) is present in the N-terminal region, and an IQ motif is predicted just after the motor domain. The C-terminal region contains an unconventional myosin tail domain (Myosin TH1 superfamily domain - cl26987) with a WW domain (cd00201) and a FYVE_like_SF (cd00065). This protein also contains a putative C-terminal coiled-coil forming region (Supplementary Table S1).

The size of the Myo1 protein from the *Trypanosoma* species, *Blechomonas ayalai* and *Bodo saltans* varies from 1,153 to 1,174 aa, with the expected domains and motifs conserved, except for the absence of IQ motif prediction to *Bl. ayalai* Myo1. Myo1 from *B. saltans* has also a MAP7 (E-MAP-115) family domain (pfam05672) in the C-terminus of the protein. *T. rangeli* presents a very short sequence that corresponds to Myo1. Since the *T. rangeli* genome is composed of short contigs, we cannot discard the hypothesis that the truncated Myo1 protein could be an artefact of genome sequencing/assembly.

Endotrypanum monterogeii, *Crithidia fasciculata*, *Leptomonas* and *Leishmania* species contain proteins of approximately 1,370 aa. There was an increment of approximately 200 aa, focused mainly in two regions: an insertion of approximately 70 aa in the head domain and a C-terminal 90-aa insertion. The first insertion is a low complexity region (as predicted by SMART) rich in alanine, glycine, serine and aspartic acid short repetitions and leads to a gap into the motor head domain prediction. WW and FYVE_like_SF domains are present in almost all species, and the Myosin_TH1 superfamily domain appears less conserved. IQ motif was not predicted for some *Leishmania* species.

Strigomonas culicis Myo1 searches revealed three hits that corresponded to Myo1 (EPY17539.1, EPY17540.1, EPY17541.1). These sequences are truncated parts of the Myo1 protein and represent assembly problems, since we were able to find a complete sequence in other genome assemblies by Local Blast searches (genome information available in Supplementary Table S2). A complete Myo1 gene was found encoding a large protein with 1,550 aa, indicating a possible assembly problem. This sequence contains several insertions, from approximately 6 to 40 aa, spread throughout the entire protein. These

insertions coincide with low complexity regions identified by SMART. Myosin motor and FYVE_like_SF domains and IQ motifs are conserved (Supplementary Table S1).

The *Angomonas deanei* Myo1 sequence also presents some regions of insertion but lacks the last 200 aa in comparison to *T. cruzi* Myo1, totalling 1,127 aa. A Myo1 protein retrieved by Local Blast of another *A. deanei* genome assembly presents only 950 aa with an even larger C-terminal deletion. This deletion includes the coiled-coil region that is found in almost all other trypanosomatid Myo1; nonetheless, it is not possible to discard the hypothesis that this deletion is an artefact of genome assembly defects. Myosin motor and WW domains and IQ motifs are predicted.

Class I myosins were the first unconventional myosins to be discovered[1]. Class I myosins are single-headed with a wide diversity of tails lengths and specialized domains, showing a variety of functions from cell growth, development and movement in *Dictyostelium discoideum* to membrane trafficking by endocytosis and exocytosis in a variety of organisms, for example, *D. discoideum*, *Saccharomyces cerevisiae, Aspergillus nidulans*[2] and *Entamoeba histolytica*[3]. In *T. brucei*, class I myosin can play a role in the endocytic pathway[4], while in *L. donovani* the gene seems not to be expressed[5]. Despite the putative differences in gene expression, almost all kinetoplastids possess similar domain architecture, but *Leishmania* species present a gap in the predicted myosin head domain that can lead to some different functionality.

Most Kinetoplastida Myo1 proteins share a disruption on the TH1 domain by a FYVE-like SF domain. The kinetoplastid Myo1, together with some myosins from Stramenopiles (*Phytophthora* species and *Hyaloperonospora parasitica*), are the only known myosins that have a FYVE or FYVE-like domain[6]. The classical FYVE domain has eight conserved cysteines that coordinate two Zn^{2+} ions[7] and three additional conserved elements: an N-terminal WxxD, a central basic RR/KHHCR and a C-terminal RVC motif that together allow the binding of phosphatidylinositol 3-phosphate (also termed PI3P)[8]. PI3P is specifically located on membranes of the endocytic pathway and is involved in regulating membrane trafficking and signal transduction[9]. However, two different types of FYVE-like domains are found in Myo1 from the species analysed here (Supplementary Table S1), characterized by the lack of the three canonical elements necessary for the binding of PI3P. Despite the involvement of *T. brucei* Myo1 in the endocytic

pathway[4], a finding in agreement with the localization of FYVE-containing proteins and PI3P, the precise function of the FYVE-like domain and its ligand in myosins still needs to be elucidated.

Kinetoplastid Myo1 also has a WW domain (with exception of *S. culicis*) right before the FYVE-like domain (Figure 2), disrupting the TH1 domain in some cases. WW domains are named after their two conserved tryptophans (W) and mediate protein-protein interactions by recognizing proline-rich peptide motifs and phosphorylated serine/threonine-proline sites[10]. WW domains are functionally similar to SH3 domains (commonly associated with myosins)[6,11] and seem to be involved in a wide variety of cellular functions, from linking cell signalling to cytoskeleton[12] to involvement in transcription[13]. WW domains are also found in myosins of a variety of groups[11], but the function of this domain in myosins still needs to be determined.

Class XXXVI myosin functions and domains

T. cruzi MyoG (TCDM_02016) gene has 3,669 bp encoding a 1,222-aa protein. Only the myosin motor domain is found with high significance. With less significance, two non-related domains are predicted for the same region (TPH, pfam13868 and SMC_prok_B, TIGR02168, Supplementary Table S1). The predicted CL Brener MyoG protein has an additional 92 aa in the N-terminal. Analysing other Dm28c genome assembly (GCA_002219105.1), we confirmed that the correct Dm28c MyoG protein probably has 1,309 aa.

There is still no study that has addressed the function of this gene. Some clues could come from domain composition analyses. MyoG protein from trypanosomatids presents overlapping domains with the best hits (minor E-value) corresponding to members of the ERM superfamily, especially the TPH domain (Trichohyalin-plectin-homology, pfam13868). According to CDD description, this domain in non-myosin proteins seems to be involved in mitochondrial movement through its binding to actin intermediate filaments[14], and its possible presence in myosin proteins was not predicted before. Searching for domain composition of MyoG-derived proteins resulted in non-recognizable domain structure. However, the data

presented here about the domain composition of all evaluated myosins are only predictions made by online search in CDD, among other databases, and need to be validated by biological experiments.

Class XIII myosin functions and domains

The *T. cruzi* Myo13 gene has 3,177 bp encoding a 1,058-aa protein containing the myosin motor domain (MYSc_Myo13, cd14875), followed by a coiled-coil region and two tandem UBA superfamily domains (cl21463) (Supplementary Table S1). Odronitz and Kollmar (2007) also identified, by manual search, an N-terminal SH3-like domain[6]. SH3 domains have conserved domain structure, but their variable sequences make them difficult to identify[15], which could explain why we were not able to find them in our searches. This gene was found in almost all analysed species. *A. deanei* and *S. culicis* presented three hits and two hits, respectively, corresponding to Myo13 proteins. Local Blast searches in other genome assemblies indicate that only one Myo13 gene copy is present in these species.

Myo13 sequences from most species are of similar size and presenting the same domains. For some species, an additional domain is found (with a higher e-value, around E-3) overlapping the coiled-coil region: Synaphin superfamily, cl05420, for *T. cruzi*; DUF812 superfamily, cl25503, for *A. deanei*; Neuromodulin_N superfamily, cl26511, for *L. enriettii*; and Mitofilin superfamily, cl26613, for *Bl. ayalai*. Therefore, the real presence of these domains should be better investigated. *B. saltans* Myo13 has an insertion of 160 aa in the C-terminal region in comparison to *T. cruzi* Myo13 and a deletion of the last 60 aa. This insertion coincides with the region that presents a DNA_pol3_gamma3 superfamily domain (cl26386). The Myo13 gene is absent in *T. congolense,* and no remnants of this gene were found in the syntenic region. Myo13 also appears to be absent in *T. rangeli*; nonetheless, it is not possible to discard the hypothesis that the gene was missed in the assembly.

The UBA domain is responsible for the binding of ubiquitin, a molecule that usually targets proteins for proteasomal degradation. Ubiquitination (the process of tagging the ubiquitin molecule to a protein) has been associated with a number of cellular processes, including DNA repair, vesicle fusion and endocytosis[16]. This wide spectrum of function requires high substrate specificity and diverse downstream effectors that interact with different substrates. The molecular effectors are composed of a modular combination of

ubiquitin-binding motifs, such as the UBA domain[17]. In myosins, the presence of the UBA domain is exclusive to Kinetoplastida and found only in Myo13 genes[18,6,15,11].

T. cruzi Dm28c MyoA gene (TCDM_09957) has 3,012 bp encoding a 1,003-aa protein. The *T. cruzi* CL Brener MyoA protein possess 1,072 aa and indicates that the Dm28c protein has a C-terminal truncation. Analysis of the other Dm28c genome assembly (GCA_002219105.1) confirmed that the correct Dm28c MyoA protein also has 1,072 aa. The myosin motor domain (cd00124) is present in the N-terminal region, and the protein also contains an IQ motif and two coiled-coil regions. By manual search, Odronitz and Kollmar (2007) also identified an N-terminal SH3-like domain[6]. The size and domain/motif composition of MyoA from *B. saltans, T. grayi* and *T. theileri* are similar to *T. cruzi*. Less-confident domains overlapping the coiled-coil region are found for some species such as the SMC_N superfamily (cl25732) for *T. theileri*, Neuromodulin_N superfamily (cl26511) for *T. grayi* and DUF4670 superfamily (cl25548) for *B. saltans.*

T. cruzi Dm28c MyoB gene (TCDM_ 07433) has 3,357 bp encoding a 1,118-aa protein. Only the myosin motor domain (cd00124) is predicted in this sequence. The CL Brener strain MyoB protein possesses 1,228 aa, and the analysis of the Dm28c GCA_002219105.1 assembly suggested a deletion in the C-terminal portion of Dm28c MyoB. Odronitz and Kollmar (2007) also identified, by manual search, an SH3-like domain in the N-terminal portion of MyoB[6]. A coiled-coil region is predicted in the C-terminal regions of MyB from *T. grayi, T. theileri* and *B. saltans,* and an IQ motif is predicted in the sequences from *T. cruzi* CL Brener and marinkellei strains, *T. grayi* and *T. theileri*. B. saltans MyoB also has DUF390 (cl25642) and Herpes_BLLF1 (cl25496) superfamily domains.

T. cruzi MyoC gene (TCDM_02877) has 3,304 bp encoding a 1,167-aa protein. The myosin motor domain (cd00124) is present in the N-terminal region, and a coiled-coil region is present downstream (Supplementary Table S1). The predicted proteins from *T. grayi, T. theileri* and *B. saltans* have similar structure except for the possible presence of additional domains for some species that were predicted with less significant e-values, and the IQ motif was predicted only in *B. saltans* MyoC.

T. cruzi MyoD gene (TCDM_07686) has 3,678 bp encoding a 1,225-aa protein. The myosin motor domain (cd00124) is present in the N-terminal region, and an IQ motif is predicted downstream. The head

domain and IQ motif were also found for *T. rangeli*, *T. grayi*, *T. theileri* and *B. saltans* proteins, and a coiled-coil region was predicted in MyoD from *T. cruzi* marinkellei, *T. grayi* and *T. theileri*.

T. cruzi MyoE gene (TCDM_07686) has 3,288 bp encoding a 1,095-aa protein. The myosin motor domain (cd00124) is present in the N-terminal region, followed by two IQ motifs. *T. rangeli*, *T. grayi*, *T. theileri* and *B. saltans* proteins also have the same structure. Additional coiled-coil region are predicted in *T. rangeli* and *T. theileri* proteins. For *B. saltans* MyoE, a myosin N-terminal SH3-like domain (pfam02736) was also predicted. Some sequences also contain additional domains that are predicted with less confidence.

T. cruzi MyoF gene (TCDM_08875) has 4,446 bp encoding a 1,481-aa protein. Myosin motor domain (cd00124) is present in the N-terminal region. An IQ motif and two coiled-coil domains are also predicted. The predicted proteins for *T. theileri* and *T. grayi* have similar structures to *T. cruzi* MyoF; however, for *T. grayi*, two additional domains are predicted (DUF4670 superfamily, cl25548 and Neuromodulin_N superfamily, cl26511). *B. saltans* MyoF is longer than *T. cruzi* MyoF (1,677 aa), and the domain prediction revealed significant differences in the C-terminal region. For this protein, a myosin motor domain (cd00124) is predicted, followed by a SMC_N superfamily (cl25732) domain and with less support, a Rad50_zn_hook superfamily (cl27157) domain. A C-terminal truncated version of the MyoF gene is also found in *T. rangeli* encoding only part of the myosin head domain.

The exact domain composition of the other members of Class XIII myosins mentioned above is hard to determine, with only the myosin motor domain (cd00124), IQ motif and coiled-coils being predicted with significant e-values (<E-05). Myosins with overlapping domains have the superimposed sequences not represented in Figure 3 of the main text. The SMC family domain that was predicted for some proteins is found in proteins that form coiled-coil structures (two α-helices). In molecular motors, coiled-coils are known to dimerize proteins. However, the prediction of a coiled-coil domain is not a simple task, and some myosins predicted to form coiled-coils actually have an SAH (single α-helical domain) and do not dimerize[19]. In trypanosomatids, the exact composition and function of these structures and domains in myosins still need to be clarified.

Myosin superfamily evolution

Previous works have addressed the evolution of myosin genes since the origin of eukaryotic life. However, some authors disagree about the number and domain composition of myosins from the Last Eukaryotic Common Ancestor (LECA). For example, Richards and Cavalier-Smith (2005) and Foth *et al*. (2006) support the idea of three ancient myosin subfamilies present in the LECA: an orthologue to the current Class I myosins that have a membrane-binding TH1 domain (disrupted by a FYVE-like domain in Kinetoplastida), a myosin-containing dilute domain and coiled-coil (lost by Excavates and Chromalveolates), and a third myosin with a MYTH4/FERM domain that had multiple secondary losses (including Excavata) or gene modification[15,18]. Odronitz and Kollmar (2014) proposed that in the beginning of eukaryotic evolution, the myosin motor domain had developed followed by subsequent domain fusions, including the C-terminal IQ motif as one of the first modifications. Next, this myosin was duplicated, and an N-terminal SH3-like domain was fused to the motor domain. They also suggested that the most ancient myosin motor domain had a sequence similar to Class I myosins, which has the widest taxonomic distribution and was probably the first myosin class to have evolved[6].

Sebé-Pedrós et al. (2014), on the other hand, based in a different rooted unikont-bikont tree proposed that the LECA had at least six myosin types: three similar to the other proposition and three more myosin types, suggesting that the LECA had a complex and diversified actin and tubulin cytoskeleton with complex eukaryotic cellular characteristics even before the diversification of the eukaryotic groups. They also reconstructed the LBikCA (Last Bikont Common Ancestor), the last common ancestor of Excavata, Alveolata, Heterokonta and Viridiplantae, with the same six LECA myosins: 1) an ancestral myosin I (progenitor paralog of the myosins I a/b/c/h/d/g/k); 2) a myosin If; 3) a myosin II; 4) a myosin IV; 5) a myosin V-like; and 6) a myosin VI. Posterior diversification and loses of the myosin classes have occured and the ancestor of Excavata was supost to have lost the myosins IV, V-like and VI. In the last common ancestor of Kinetoplastida and Heterolobosea, the class XIII has appeared and in the Kinetoplastids myosin I a/b/c/h/d/g/k progenitor was lost. According to our data, it must be considered the origin of Class XXXVI in the ancestor of Kinetoplastida.

The diversification of the myosin family is also seen in the concurrent domains (domains that appear together with the myosin head domain), as shown by Sebé-Pedrós et al. (2014), who analysed the concurrent domains within taxa and found that some of the shared protein domains were acquired by convergence. Additionally, they stated that little innovation in protein domain combinations is found within bikonts[11], which is clearly not the case for Class XIII myosins from Kinetoplastida, which we present here with a number of unusual domains being predicted for myosins.

Supplementary figure

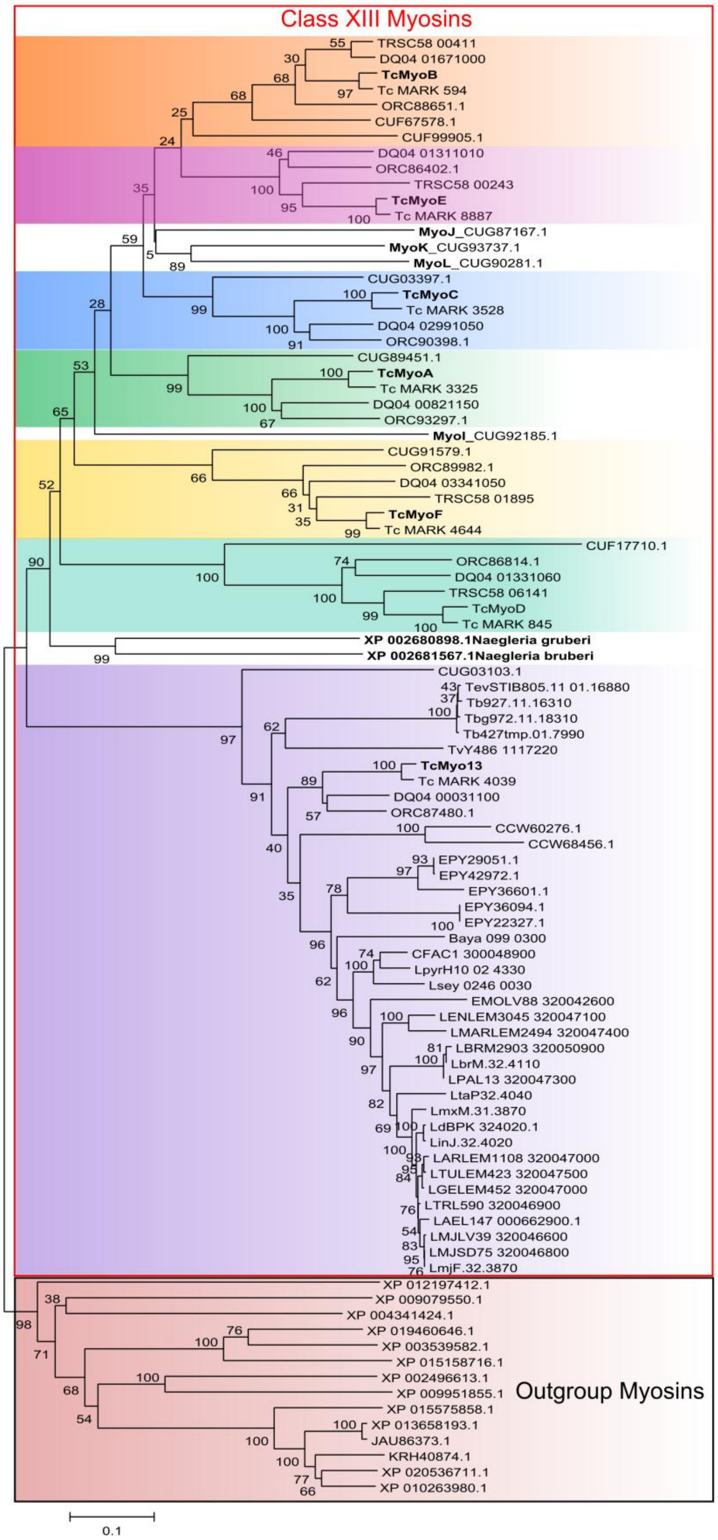

Figure S1. Neighbor-joining tree (Poisson correction method) of kinetoplastid Class XIII myosins and outgroups. This phylogenetic tree includes the kinetoplastid class XIII myosins, the two *Naegleria gruberi* sequences and the next two sequences found for each BlastP searches (from Myo13 and MyoA to MyoF) and was constructed to discard the possibility that some other sequence, in addition to the *Naegleria*, would be placed inside the kinetoplastid clade. Bootstrap values (1,000 replicates) are shown next to the branches. The analysis involved 93 amino acid sequences. All ambiguous positions were removed for each sequence pair. There were 960 positions in the final dataset. Evolutionary analyses were conducted in MEGA7[20].

References

1. Thompson, R. F. & Langford, G. M. Myosin superfamily evolutionary history. *Anat. Rec.* **268,** 276–89 (2002).
2. Mermall, V., Post, P. L. & Mooseker, M. S. Unconventional myosins in cell movement, membrane traffic, and signal transduction. *Science* **279,** 527–33 (1998).
3. Voigt, H. & Guillén, N. New insights into the role of the cytoskeleton in phagocytosis of Entamoeba histolytica. *Cell. Microbiol.* **1,** 195–203 (1999).
4. Spitznagel, D., O'Rourke, J. F., Leddy, N., Hanrahan, O. & Nolan, D. P. Identification and characterization of an unusual class I myosin involved in vesicle traffic in Trypanosoma brucei. *PLoS One* **5,** (2010).
5. Katta, S. S., Sahasrabuddhe, A. A. & Gupta, C. M. Flagellar localization of a novel isoform of myosin, myosin XXI, in Leishmania. *Mol. Biochem. Parasitol.* **164,** 105–110 (2009).
6. Odronitz, F. & Kollmar, M. Drawing the tree of eukaryotic life based on the analysis of 2,269 manually annotated myosins from 328 species. *Genome Biol.* **8,** R196 (2007).
7. Driscoll, P. C. Solving the FYVE domain--PtdIns(3)P puzzle. *Nat. Struct. Mol. Biol. Mol. Biol.* **8,** 287–290 (2001).
8. Kutateladze, T. G. Phosphatidylinositol 3-phosphate recognition and membrane docking by the FYVE domain. *Biochim Biophys Acta* **1761,** 868–877 (2006).
9. Gillooly, D. J., Simonsen, A. & Stenmark, H. Phosphoinositides and phagocytosis. *J. Cell Biol.* **155,** 15–7 (2001).
10. Meng, G. *et al.* Genome-wide analysis of the WW domain-containing protein genes in silkworm and their expansion in eukaryotes. *Mol. Genet. Genomics* **290,** 807–824 (2015).
11. Sebé-Pedrós, A., Grau-Bové, X., Richards, T. A. & Ruiz-Trillo, I. Evolution and classification of myosins, a paneukaryotic whole-genome approach. *Genome Biol. Evol.* **6,** 290–305 (2014).
12. Ilsley, J. L., Sudol, M. & Winder, S. J. The WW domain: linking cell signalling to the membrane cytoskeleton. *Cell. Signal.* **14,** 183–9 (2002).
13. Sudol, M., Sliwa, K. & Russo, T. Functions of WW domains in the nucleus. *FEBS Lett.* **490,** 190–5 (2001).
14. Marchler-Bauer, A. *et al.* CDD/SPARCLE: Functional classification of proteins via subfamily domain architectures. *Nucleic Acids Res.* **45,** D200–D203 (2017).
15. Richards, T. a & Cavalier-Smith, T. Myosin domain evolution and the primary divergence of eukaryotes. *Nature* **436,** 1113–8 (2005).
16. Weissman, a M. Themes and variations on ubiquitylation. *Nat. Rev. Mol. Cell Biol.* **2,** 169–178 (2001).
17. Buchberger, A. From UBA to UBX: New words in the ubiquitin vocabulary. *Trends Cell Biol.* **12,** 216–221 (2002).

18. Foth, B. J., Goedecke, M. C. & Soldati, D. New insights into myosin evolution and classification. *Proc. Natl. Acad. Sci. U. S. A.* **103,** 3681–6 (2006).
19. Peckham, M. Coiled coils and SAH domains in cytoskeletal molecular motors. *Biochem. Soc. Trans.* **39,** 1142–1148 (2011).
20. Kumar, S., Stecher, G. & Tamura, K. MEGA7: Molecular Evolutionary Genetics Analysis Version 7.0 for Bigger Datasets. *Mol. Biol. Evol.* **33,** 1870–1874 (2016).

Supplementary Tables

Table S1: Domain prediction for myosin proteins (provided as xls file)

Table S2: Information of genome sequences used in this work

Table S3: Summary of BlastP results for Myo1 against sequences available at TritrypDB and NCBI

Table S4: Synteny conservation analysis of Myo1 gene

Table S5: Summary of BlastP results for MyoG against sequences available at TritrypDB and NCBI

Table S6: Synteny conservation analysis of MyoG gene

Table S7: Summary of BlastP results for Myo13 against sequences available at TritrypDB and NCBI

Table S8: Synteny conservation analysis of Myo13 gene

Table S9: Summary of BlastP results for MyoA against sequences available at TritrypDB and NCBI

Table S10: Synteny conservation analysis of MyoA gene

Table S11: Summary of BlastP results for MyoB against sequences available at TritrypDB and NCBI

Table S12: Synteny conservation analysis of MyoB gene

Table S13: Summary of BlastP results for MyoC against sequences available at TritrypDB and NCBI

Table S14: Synteny conservation analysis of MyoC gene

Table S15: Summary of BlastP results for MyoD against sequences available at TritrypDB and NCBI

Table S16: Synteny conservation analysis of MyoD gene

Table S17: Summary of BlastP results for MyoE against sequences available at TritrypDB and NCBI

Table S18: Synteny conservation analysis of MyoE gene

Table S19: Summary of BlastP results for MyoF against sequences available at TritrypDB and NCBI

Table S20: Synteny conservation analysis of MyoF gene

Table S21: Summary of BlastP results for MyoH-derived against sequences available at TritrypDB and NCBI

Table S22: Synteny conservation analysis of MyoH-derived gene

Table S2: Information of genome sequences used in this work

Source: TritrypDB			
**Organism**	**Release**	**Summary**	**Contact**
T. cruzi Dm28c	TriTrypDB 9.0 / 14-JAN-15	*Trypanosoma cruzi* Dm28c Sequence and Annotation	Edmundo Carlos Grisard
L. seymouri ATCC 30220	TriTrypDB 25 / 23-JUL-15	*Leptomonas seymouri* ATCC 30220 Sequence and Annotation	Vyacheslav Yurchenko
T. cruzi strain CL Brener	not available	Sequence and annotation of *Trypanosoma cruzi* CL Brener .	GeneDB
L. enriettii strain LEM3045	TriTrypDB 7.0 / 03-FEB-14	*Leishmania enriettii* strain LEM3045 sequence and annotation	Stephen M. Beverley
L. pyrrhocoris H10	TriTrypDB 25 / 23-JUL-15	*Leptomonas pyrrhocoris* H10 Sequence and Annotation	Julius Lukes
T. brucei Lister strain 427	not available	Genome sequence and annotation for *Trypanosoma brucei* strain Lister 427	Pathogen Sequencing Unit
B. ayalai B08-376	TriTrypDB 31 / 09-MAR-17	*Blechomonas ayalai* B08-376 Sequence and Annotation	Vyacheslav Yurchenko
L. braziliensis MHOM/BR/75/M2904	not available	*Leishmania braziliensis* M2904 (MHOM/BR/75M2904) sequence and annotation from GeneDB.	Pathogen Sequencing Unit
L. donovani strain BHU 1220	TriTrypDB 24 / 14-APR-15	Genomic Sequence of *Leishmania donovani* BHU 1220	Neeloo Singh
L. sp. MAR LEM2494	TriTrypDB 6.0 / 25-SEP-13	*Leishmania sp.* MAR LEM2494 Genome sequence and annotation	Stephen M. Beverley
L. aethiopica L147	TriTrypDB 7.0 / 03-FEB-14	Genome Sequence and Annotation of *Leishmania aethiopica* L147	Stephen M. Beverley
T. grayi ANR4	TriTrypDB 8.0 / 08-MAY-14	*Trypanosoma grayi* ANR4 Sequence and Annotation	Mark Field
L. tropica L590	TriTrypDB 7.0 / 03-FEB-14	Genome Sequence and Annotation of *Leishmania tropica* L590	Stephen M. Beverley
T. rangeli SC58	TriTrypDB 9.0 / 14-JAN-15	*Trypanosoma rangeli* SC58 Sequence and Annotation	Edmundo Carlos Grisard
L. donovani BPK282A1	TriTrypDB 5.0 / 31-MAY-13	*Leishmania donovani* BPK282A1 Sequence and Annotation	Matthew Berriman
L. braziliensis MHOM/BR/75/M2903	TriTrypDB 5.0 / 31-MAY-13	*Leishmania braziliensis* M2903 Genome sequence and annotation	Stephen M. Beverley
T. congolense IL3000	not available	*Trypanosoma congolense* IL3000 Sequence and Annotation	Matthew Berriman
T. brucei gambiense DAL972	not available	Genome sequence and annotation for *Trypanosoma brucei* gambiense	Pathogen Sequencing Unit
L. turanica strain LEM423	TriTrypDB 7.0 / 03-FEB-14	Genome Sequence and Annotation for *Leishmania turanica* strain LEM423	Stephen M. Beverley
L. infantum JPCM5	not available	Genome sequence and annotation for *Leishmania infantum* JPCM5 (MCAN/ES/98/LLM-877)	Pathogen Sequencing Unit
L. major strain SD 75.1	not available	Genome sequence and annotation for *Leishmania major* SD 75.1	Stephen M. Beverley
T. cruzi JR cl. 4	not available	Genome Sequence for *Trypanosoma cruzi* JR cl4	Gregory A. Buck
L. tarentolae Parrot-TarII	not available	*Leishmania tarentolae* Parrot-TarII Sequence and Annotation	Jacques Corbeil
T. cruzi Sylvio X10/1	not available	*Trypanosoma cruzi* Sylvio X10/1 Sequence and Annotation	Bjorn Andersson
L. amazonensisMHOM/BR/71973/M2269	TriTrypDB 8.0 / 08-MAY-14	Genome sequence of *Leishmania amazonensis* MHOM/BR/71973/M2269	Diana Bahia
L. panamensis MHOM/COL/81/L13	not available	*Leishmania panamensis* L13 Genome sequence and annotation	Stephen M. Beverley
L. major strain LV39c5	not available	*Leishmania major* LV39c5 Genome Sequence and Annotation	Stephen M. Beverley
T. cruzi Tula cl2	TriTrypDB 7.0 / 03-FEB-14	Genome Sequence of *Trypanosoma cruzi* Tula cl2	Stephen M. Beverley
T. brucei brucei TREU927	not available	*Trypanosoma brucei* TREU927 sequence and annotation	Pathogen Sequencing Unit
T. cruzi strain Esmeraldo	not available	Genome sequence for *Trypanosoma cruzi* Esmeraldo strain cl3	Gregory A. Buck
L. major strain Friedlin	not available	Genome sequence and annotation for *Leishmania major* Friedlin.	Pathogen Sequencing Unit
T. cruzi marinkellei strain B7	TriTrypDB 5.0 / 31-MAY-13	*Trypanosoma cruzi* marinkellei Sequence and Annotation	Oscar Frazen
L. gerbilli strain LEM452	TriTrypDB 7.0 / 03-FEB-14	Genome Sequence and Annotation for *Leishmania gerbilli* strain LEM452	Stephen M. Beverley
L. arabica strain LEM1108	TriTrypDB 7.0 / 03-FEB-14	Genome Sequence and Annotation for *Leishmania arabica* strain LEM1108	Stephen M. Beverley
T. cruzi CL Brener Esmeraldo-like	not available	Genome sequence and annotation for *Trypanosoma cruzi* CL Brener.	Pathogen Sequencing Unit
C. fasciculata strain Cf-Cl	TriTrypDB 5.0 / 31-MAY-13	*Crithidia fasciculata* Cf-C1 sequence and annotation	Stephen M. Beverley
L. mexicana MHOM/GT/2001/U1103	not available	Genome sequence and annotation for *Leishmania mexicana* U1103	Pathogen Sequencing Unit
T. vivax Y486	not available	Genome sequence and annotation for *Trypanosoma vivax* Y486	Pathogen Sequencing Unit
E. monterogeii strain LV88	not available	*Endotrypanum monterogeii* LV88 Genome Sequence and Annotation	Stephen M. Beverley
T. cruzi CL Brener Non-Esmeraldo-like	not available	Genome sequence and annotation for the non-Esmeraldo haplotype of *Trypanosoma cruzi* CL Brener	GeneDB

T. evansi strain STIB 805	TriTrypDB 6.0 / 25-SEP-13	*T. evansi* STIB 805 Sequence and Annotation	Achim Schnaufer
Source: NCBI - Online BLAST Searches			
Organism	**GenBank assembly accession**	**Genome coverage/Assembly level**	**Submitter**
Trypanosoma theileri Edinburgh	GCA_002087225.1	100x/ Scaffold	University of Edinburgh
Angomonas deanei	GCA_000442575.2	23x/Scaffold	LNCC
Strigomonas culicis	GCA_000442495.1	23x/Scaffold	LNCC
Phytomonas sp. isolate Hart1	GCA_000982615.1	not available/Scaffold	Genoscope CEA
Phytomonas sp. isolate EM1	GCA_000582765.1	not available/Scaffold	Genoscope CEA
Bodo saltans Lake Konstanz	GCA_001460835.1	not available/Contig	Wellcome Trust Sanger Institute
Source: NCBI - Local BLAST Searches			
Organism	**GenBank assembly accession**	**Genome coverage/Assembly level**	**Submitter**
Trypanosoma theileri Edinburgh	GCA_002087225.1	100x/ Scaffold	University of Edinburgh
Angomonas deanei ATCC PRA-265	GCA_001659865.1	not available/Scaffold	Heinrich Heine University Duesseldorf
Strigomonas culicis TCC012E	GCA_000482145.1	not available/ Contig	Virginia Commonwealth University
Phytomonas sp. isolate Hart1	GCA_000982615.1	not available/Scaffold	Genoscope CEA
Phytomonas sp. isolate EM1	GCA_000582765.1	not available/Scaffold	Genoscope CEA
Bodo saltans Lake Konstanz	GCA_001460835.1	not available/Contig	Wellcome Trust Sanger Institute
Trypanosoma cruzi Dm28c PB1	GCA_002219105.1	not available/Contig	Instituto Carlos Chagas - ICC/Fiocruz-PR

Table S3: Summary of BlastP results for Myo1 against sequences available at TritrypDB and NCBI

TritrypDB			
**Genomes**	**BlastP e-value**	**ID of Hit**	**Reciprocal Blast/Synteny**
**Green boxes are the hits that correspond to Myo1 genes**			
Blechomonas ayalai B08-376	0.0	Baya_227_0080	Myo1 (TCDM_07314)
	2,00E-85	Baya_099_0300	Myo13 (TCDM_05821)
Crithidia fasciculata strain Cf-Cl	0.0	CFAC1_290040800	Myo1 (TCDM_07314)
	9,00E-77	CFAC1_300048900	Myo13 (TCDM_05821)
Endotrypanum monterogeii strain LV88	0.0	EMOLV88_340015100	Myo1 (TCDM_07314)
	2,00E-76	EMOLV88_320042600	Myo13 (TCDM_05821)
Leishmania aethiopica L147	0.0	LAEL147_000713700	Myo1 (TCDM_07314)
	2,00E-80	LAEL147_000662900.1	Myo13 (TCDM_05821)
Leishmania arabica strain LEM1108	0.0	LARLEM1108_000017500	Myo1 (TCDM_07314)
	1,00E-79	LARLEM1108_320047000	Myo13 (TCDM_05821)
Leishmania braziliensis MHOM/BR/75/M2903	0.0	LBRM2903_200016800	Myo1 (TCDM_07314)
	9,00E-70	LBRM2903_320050900	Myo13 (TCDM_05821)
Leishmania braziliensis MHOM/BR/75/M2904	0.0	LbrM.20.0970	Myo1 (TCDM_07314)
	2,00E-83	LbrM.32.4110	Myo13 (TCDM_05821)
Leishmania donovani BPK282A1	0.0	LdBPK_341070.1	Myo1 (TCDM_07314)
	5,00E-83	LdBPK_324020.1	Myo13 (TCDM_05821)
Leishmania enriettii strain LEM3045	0.0	LENLEM3045_340014600	Myo1 (TCDM_07314)
	5,00E-84	LENLEM3045_320047100	Myo13 (TCDM_05821)
Leishmania gerbilli strain LEM452	0.0	LGELEM452_000011900	Myo1 (TCDM_07314)
	3,00E-80	LGELEM452_320047000	Myo13 (TCDM_05821)
Leishmania infantum JPCM5	0.0	LinJ.34.1070	Myo1 (TCDM_07314)
	2,00E-83	LinJ.32.4020	Myo13 (TCDM_05821)
Leishmania major strain Friedlin	0.0	LmjF.34.1000	Myo1 (TCDM_07314)
	2,00E-80	LmjF.32.3870	Myo13 (TCDM_05821)
Leishmania major strain LV39c5	0.0	LMJLV39_340016900	Myo1 (TCDM_07314)
	1,00E-80	LMJLV39_320046600	Myo13 (TCDM_05821)
Leishmania major strain SD 75.1	0.0	LMJSD75_340016900	Myo1 (TCDM_07314)
	6,00E-81	LMJSD75_320046800	Myo13 (TCDM_05821)
Leishmania mexicana MHOM/GT/2001/U1103	0.0	LmxM.33.1000	Myo1 (TCDM_07314)
	2,00E-81	LmxM.31.3870	Myo13 (TCDM_05821)
Leishmania panamensis MHOM/COL/81/L13	0.0	LPAL13_200014600	Myo1 (TCDM_07314)
	1,00E-84	LPAL13_320047300	Myo13 (TCDM_05821)
Leishmania tarentolae Parrot-TarII	0.0	LtaP34.1150	Myo1 (TCDM_07314)
	4,00E-78	LtaP32.4040	Myo13 (TCDM_05821)
	1,00E-62	LtaP34.1040	Myo1 (TCDM_07314)
Leishmania tropica L590	0.0	LTRL590_340015400	Myo1 (TCDM_07314)
	9,00E-82	LTRL590_320046900	Myo13 (TCDM_05821)
Leishmania turanica strain LEM423	0.0	LTULEM423_000006800	Myo1 (TCDM_07314)
	5,00E-80	LTULEM423_320047500	Myo13 (TCDM_05821)
Leishmania sp. MAR LEM2494	0.0	LMARLEM2494_340015700	Myo1 (TCDM_07314)
	6,00E-81	LMARLEM2494_320047400	Myo13 (TCDM_05821)

Leptomonas pyrrhocoris	0.0	LpyrH10_05_1440	Myo1 (TCDM_07314)
	4,00E-77	LpyrH10_02_4330	Myo13 (TCDM_05821)
Leptomonas seymouri ATCC 30220	0.0	Lsey_0435_0030	Myo1 (TCDM_07314)
	4,00E-80	Lsey_0246_0030	Myo13 (TCDM_05821)
Trypanosoma brucei Lister strain 427	0.0	Tb427.04.3380	Myo1 (TCDM_07314)
	3,00E-78	Tb427tmp.01.7990	Myo13 (TCDM_05821)
Trypanosoma brucei brucei TREU927	0.0	Tb927.4.3380	Myo1 (TCDM_07314)
	1,00E-78	Tb927.11.16310	Myo13 (TCDM_05821)
Trypanosoma brucei gambiense DAL972	0.0	Tbg972.4.3390	Myo1 (TCDM_07314)
	5,00E-78	Tbg972.11.18310	Myo13 (TCDM_05821)
Trypanosoma congolense IL3000	0.0	TcIL3000_4_3080	Myo1 (TCDM_07314)
Trypanosoma cruzi marinkellei strain B7	5,00E-162	Tc_MARK_2655	Myo1 (TCDM_07314)
	2,00E-110	Tc_MARK_2656	Myo1 (TCDM_07314)
	4,00E-108	Tc_MARK_3325	MyoA (TCDM_09957)
	9,00E-98	Tc_MARK_3528	MyoC (TCDM_02877)
	3,00E-95	Tc_MARK_594	MyoB (TCDM_07433)
	6,00E-87	Tc_MARK_845	MyoD (TCDM_07686)
	8,00E-86	Tc_MARK_8887	MyoE (TCDM_06166)
	1,00E-79	Tc_MARK_4039	Myo13 (TCDM_05821)
	5,00E-75	Tc_MARK_4644	MyoF (TCDM_08875)
	3,00E-45	Tc_MARK_4094	MyoG (TCDM_02016)
Trypanosoma evansi strain STIB 805	0.0	TevSTIB805.4.3470	Myo1 (TCDM_07314)
	7,00E-79	TevSTIB805.11_01.16880	Myo13 (TCDM_05821)
Trypanosoma grayi ANR4	0.0	DQ04_00501100	Myo1 (TCDM_07314)
	2,00E-97	DQ04_02991050	MyoC (TCDM_02877)
	3,00E-95	DQ04_00821150	MyoA (TCDM_09957)
	3,00E-94	DQ04_01671000	MyoB (TCDM_07433)
	7,00E-90	DQ04_01311010	MyoE (TCDM_06166)
	7,00E-88	DQ04_03341050	MyoF (TCDM_08875)
	1,00E-88	DQ04_01331060	MyoD (TCDM_07686)
	2,00E-80	DQ04_00031100	Myo13 (TCDM_05821)
	5,00E-47	DQ04_03771000	MyoG (TCDM_02016)
	5,00E-04	DQ04_00271170	MyoH (TCDM_02145)
Trypanosoma rangeli SC58	2,00E-96	TRSC58_06141	MyoD (TCDM_07686)
	1,00E-90	TRSC58_00243	MyoE (TCDM_06166)
	7,00E-48	TRSC58_01895	MyoF (TCDM_08875)
	7,00E-44	TRSC58_03135	Myo1 (TCDM_07314)
	4,00E-09	TRSC58_00411	MyoB (TCDM_07433)
Trypanosoma vivax Y486	0.0	TvY486_0403180	Myo1 (TCDM_07314)
	2,00E-80	TvY486_1117220	Myo13 (TCDM_05821)
**NCBI**			
**Genomes**	**BlastP e-value**	**ID of Hit**	**Reciprocal Blast**
**Green boxes are hits that correspond to Myo1**			
**Kinetoplastids**			
Trypanosoma theileri	0.0	ORC93509.1	Myo1 (TCDM_07314)
	1,00E-102	ORC93297.1	MyoA (TCDM_09957)
	3,00E-102	ORC90398.1	MyoC (TCDM_02877)
	7,00E-98	ORC88651.1	MyoB (TCDM_07433)
	2,00E-91	ORC86402.1	MyoE (TCDM_06166)
	4,00E-88	ORC86814.1	MyoD (TCDM_07686)
	5,00E-87	ORC89982.1	MyoF (TCDM_08875)
	3,00E-82	ORC87480.1	Myo13 (TCDM_05821)
	2,00E-48	ORC91827.1	MyoG (TCDM_02016)

Angomonas deanei	0.0	EPY31408.1	Myo1 (TCDM_07314)
	2,00E-86	EPY29051.1	Myo13 (TCDM_05821)
	4,00E-84	EPY42972.1	Myo13 (TCDM_05821)
Strigomonas culicis	4,00E-96	EPY17540.1	Myo1 (TCDM_07314)
	2,00E-78	EPY36094.1	Myo13 (TCDM_05821)
	1,00E-76	EPY22327.1	Myo13 (TCDM_05821)
	5,00E-53	EPY17541.1	Myo1 (TCDM_07314)
	9,00E-19	EPY17539.1	Myo1 (TCDM_07314)
Phytomonas sp. isolate EM1	2,00E-77	CCW60276.1	Myo13 (TCDM_05821)
Phytomonas sp. isolate Hart1	2,00E-79	CCW68456.1	Myo13 (TCDM_05821)
Bodo saltans	0.0	CUI15073.1	Myo1 (TCDM_07314)
	2,00E-101	CUF67578.1	MyoB (TCDM_07433)
	1,00E-99	CUG89451.1	MyoA (TCDM_09957)
	1,00E-96	CUG93737.1	MyoB (TCDM_07433)
	3,00E-93	CUF99905.1	MyoE (TCDM_06166)
	1,00E-92	CUG90281.1	MyoB (TCDM_07433)
	4,00E-87	CUG87167.1	MyoB (TCDM_07433)
	2,00E-85	CUG91579.1	MyoF (TCDM_08875)
	3,00E-85	CUG03103.1	Myo13 (TCDM_05821)
	1,00E-84	CUG92185.1	MyoB (TCDM_07433)
	3,00E-65	CUF17710.1	MyoD (TCDM_07686)
	6,00E-65	CUG03397.1	MyoC (TCDM_02877)
	5,00E-38	CUG86977.1	MyoG (TCDM_02016)
**Examples of BlastP result against non-kinetoplastid species**			
Phytophthora infestans	0.0	XP_002906848.1	Myo1 (TCDM_07314)
Phytophthora megakarya	0.0	OWZ10650.1	Myo1 (TCDM_07314)
Plasmopara halstedii	0.0	CEG37571.1	Myo1 (TCDM_07314)
Phytophthora parasitica	0.0	ETP29088.1	Myo1 (TCDM_07314)
Phytophthora nicotianae	0.0	KUF85580.1	Myo1 (TCDM_07314)
Saprolegnia diclina VS20	0.0	XP_008615932.1	Myo1 (TCDM_07314)
Lingula anatina	0.0	XP_003286674.1	Myo1 (TCDM_07314)
Dictyostelium purpureum	0.0	XP_003286674.1	Myo1 (TCDM_07314)
Python bivittatus	0.0	XP_007435550.1	Myo1 (TCDM_07314)
Xenopus tropicalis	0.0	NP_001011082.1	Myo1 (TCDM_07314)

Table S4: Synteny conservation analysis of Myo1 gene

Genome	Upstream gene 4	Upstream gene	Myo1 gene	Downstream gene 2	Downstream gene 3
**Green boxes highlight the species that have Myo1 gene in conserved syntenic region in relation to *T. cruzi* Myo1**					
Blechomonas ayalai B08-376	Baya_033_0590	Baya_033_0540	Baya_227_0080	Baya_227_0070	Baya_227_0060
Crithidia fasciculata strain Cf-Cl	CFAC1_290039200	CFAC1_290039600	CFAC1_290040800	Non-syntenic	CFAC1_290040900
Endotrypanum monterogeii strain LV88	EMOLV88_000014100	EMOLV88_000014400	EMOLV88_340015100	Non-syntenic	EMOLV88_340015200
Leishmania aethiopica L147	LAEL147_000713100	Non-syntenic	LAEL147_000713700	Non-syntenic	LAEL147_000713800
Leishmania arabica strain LEM1108	LARLEM1108_000009300	Non-syntenic	LARLEM1108_000017500	Non-syntenic	LARLEM1108_000017400
Leishmania braziliensis MHOM/BR/75/M2903	no homologous found	LBRM2903_200016400	LBRM2903_200016800	Non-syntenic	LBRM2903_200017000
Leishmania braziliensis MHOM/BR/75/M2904	no homologous found	Non-syntenic	LbrM.20.0970	Non-syntenic	LbrM.20.0980
Leishmania donovani BPK282A1	LdBPK_341000.1	Non-syntenic	LdBPK_341070.1	Non-syntenic	LdBPK_341080.1
Leishmania enriettii strain LEM3045	LENLEM3045_340014000	Non-syntenic	LENLEM3045_340014600	Non-syntenic	LENLEM3045_340014700
Leishmania gerbilli strain LEM452	LGELEM452_000005600	Non-syntenic	LGELEM452_000011900	Non-syntenic	LGELEM452_000011800
Leishmania infantum JPCM5	LinJ.34.1000	Non-syntenic	LinJ.34.1070	Non-syntenic	LinJ.34.1080
Leishmania major strain Friedlin	LmjF.34.0950	Non-syntenic	LmjF.34.1000	Non-syntenic	LmjF.34.1010
Leishmania major strain LV39c5	LMJLV39_340016500	Non-syntenic	LMJLV39_340016900	Non-syntenic	LMJLV39_340017000
Leishmania major strain SD 75.1	LMJSD75_340016300	Non-syntenic	LMJSD75_340016900	Non-syntenic	LMJSD75_340017000
Leishmania mexicana MHOM/GT/2001/U1103	LmxM.33.0950	Non-syntenic	LmxM.33.1000	Non-syntenic	LmxM.33.1010
Leishmania panamensis MHOM/COL/81/L13	no homologous found	Non-syntenic	LPAL13_200014600	Non-syntenic	LPAL13_200014700
Leishmania tarentolae Parrot-TarII	LtaP34.1100	Non-syntenic	LtaP34.1040	Non-syntenic	LtaP34.1180
Leishmania tropica L590	LTRL590_340015000	Non-syntenic	LTRL590_340015400	Non-syntenic	LTRL590_340015500
Leishmania turanica strain LEM423	no homologous found	Non-syntenic	LTULEM423_000006800	Non-syntenic	LTULEM423_000006700
Leishmania sp. MAR LEM2494	LMARLEM2494_340015300	Non-syntenic	LMARLEM2494_340015700	Non-syntenic	LMARLEM2494_340015800
Leptomonas pyrrhocoris	LpyrH10_05_1300	LpyrH10_05_1340	LpyrH10_05_1440	Non-syntenic	LpyrH10_05_1450
Leptomonas seymouri ATCC 30220	Lsey_0178_0110	Lsey_0178_0070	Lsey_0435_0030	Non-syntenic	Lsey_0435_0020
Trypanosoma brucei Lister strain 427	Tb427.04.3410	Tb427.04.3390	Tb427.04.3380	Tb427.04.3370	Tb427.04.3360
Trypanosoma brucei brucei TREU927	Tb927.4.3410	Tb927.4.3390	Tb927.4.3380	Tb927.4.3370	Tb927.4.3360
Trypanosoma brucei gambiense DAL972	Tbg972.4.3420	Tbg972.4.3400	Tbg972.4.3390	Tbg972.4.3380	Tbg972.4.3370
Trypanosoma congolense IL3000	TcIL3000_0_57630.1	TcIL3000_4_3100	TcIL3000_4_3080	TcIL3000_4_3070	TcIL3000_4_3060
Trypanosoma cruzi CL Brener Esmeraldo-like	TcCLB.507485.100	TcCLB.507485.120	TcCLB.507739.110	TcCLB.507739.90	TcCLB.507739.80
Trypanosoma cruzi Dm28c	TCDM_07318	TCDM_07315	TCDM_07314	TCDM_07312	TCDM_07310
Trypanosoma cruzi marinkellei strain B7	Tc_MARK_2659	na	Tc_MARK_2655	Tc_MARK_2653	Tc_MARK_2652
Trypanosoma evansi strain STIB 805	TevSTIB805.4.3500	na	TevSTIB805.4.3470	TevSTIB805.4.3460	TevSTIB805.4.3450
Trypanosoma grayi ANR4	DQ04_00501050	DQ04_00501070	DQ04_00501100	DQ04_00501120	DQ04_00501130
*Trypanosoma rangeli SC58**	TRSC58_03374	TRSC58_04224	TRSC58_03135	TRSC58_05557	TRSC58_05188
Trypanosoma vivax Y486	TvY486_0403220	TvY486_0403190	TvY486_0403180	TvY486_0403170	TvY486_0403160
Trypanosoma theileri isolate Edinburgh TM35_Tth_5_V1#	NBCO01000001.1: 727589-729835	NBCO01000001.1: 734256-734828	NBCO01000001.1: 741620-744865	NBCO01000001.1 :746561-747469	NBCO01000001.1: 747754-749169
Bodo saltans#	Non-syntenic	Non-syntenic	CYKH01001760.1: 29875-29875	CYKH01001760.1:42523-43383	no homologous found
Angomonas deanei strain ATCC PRA-265#	KV452465.1:181757-184048	KV452465.1:172734-173354	KV452465.1:161708-165532	KV452465.1:159906-161135	KV452465.1:157422-158432
Strigomonas culicis strain TCC012E#	Non-syntenic	Non-syntenic	AUXH01000328.1:263-4675	AUXH01000328.1:5246-6265	Non-syntenic
Phytomas sp EM1#	no homologous found	no homogous found	no orthologous found	HF955062.1:1060963 -1061943	HF955062.1:1062905-1063984
Phytomas sp Hart1#	no homologous found	no homologous found	no orthologous found	HF955199.1: 1936723-1935848	HF955199.1:1934910-1933831

The IDs correspond to the homologous of *T. cruzi* Dm28c upstream or downstream genes. In some cases, they are not the closest gene in the analyzed species.
* Evaluation of synteny for this species was not possible since the genes were located in very small contigs.
# For these species, the approximate chromosome position is presented. Genomes obtained from NCBI.
no orthologous found - we were not able to analyze the syntenic region and no clear orthologous gene was found throughout the genome.
na- not analyzed.

Table S5: Summary of BlastP results for MyoG against sequences available at TritrypDB and NCBI

TritrypDB			
**Genomes**	**BlastP e-value**	**ID of Hit**	**Reciprocal Blast/Synteny**
**Green boxes are the hits that correspond to MyoG or MyoG-derived genes**			
Blechomonas ayalai B08-376	4,00E-59	Baya_099_0300	Myo13 (TCDM_05821)
	3,00E-51	Baya_227_0080	Myo1 (TCDM_07314)
Crithidia fasciculata strain Cf-Cl	6,00E-53	CFAC1_300048900	Myo13 (TCDM_05821)
	9,00E-22	CFAC1_290040800	Myo1 (TCDM_07314)
	1,00E-06	CFAC1_220012200	MyoG (TCDM_02016)
Endotrypanum monterogeii strain LV88	4,00E-55	EMOLV88_320042600	Myo13 (TCDM_05821)
	3,00E-24	EMOLV88_340015100	Myo1 (TCDM_07314)
	1,00E-10	EMOLV88_250010000	MyoG (TCDM_02016)
Leishmania aethiopica L147	6,00E-54	LAEL147_000662900.1	Myo13 (TCDM_05821)
	3,00E-42	LAEL147_000713700	Myo1 (TCDM_07314)
	7,00E-18	LAEL147_000404600	MyoG (TCDM_02016)
Leishmania arabica strain LEM1108	8,00E-52	LARLEM1108_320047000	Myo13 (TCDM_05821)
	1,00E-37	LARLEM1108_000017500	Myo1 (TCDM_07314)
	1,00E-19	LARLEM1108_250010200	MyoG (TCDM_02016)
Leishmania braziliensis MHOM/BR/75/M2903	1,00E-44	LBRM2903_320050900	Myo13 (TCDM_05821)
	8,00E-26	LBRM2903_200016800	Myo1 (TCDM_07314)
	4,00E-15	LBRM2903_250010500	MyoG (TCDM_02016)
Leishmania braziliensis MHOM/BR/75/M2904	1,00E-53	LbrM.32.4110	Myo13 (TCDM_05821)
	1,00E-25	LbrM.20.0970	Myo1 (TCDM_07314)
	1,00E-15	LbrM.25.0430	MyoG (TCDM_02016)
Leishmania donovani BPK282A1	3,00E-54	LdBPK_324020.1	Myo13 (TCDM_05821)
	2,00E-38	LdBPK_341070.1	Myo1 (TCDM_07314)
	3,00E-20	LdBPK_250490.1	MyoG (TCDM_02016)
Leishmania enriettii strain LEM3045	2,00E-50	LENLEM3045_320047100	Myo13 (TCDM_05821)
	4,00E-22	LENLEM3045_340014600	Myo1 (TCDM_07314)
	8,00E-17	LENLEM3045_250010000	MyoG (TCDM_02016)
Leishmania gerbilli strain LEM452	3,00E-53	LGELEM452_320047000	Myo13 (TCDM_05821)
	7,00E-37	LGELEM452_000011900	Myo1 (TCDM_07314)
	7,00E-17	LGELEM452_250010200	MyoG (TCDM_02016)
Leishmania infantum JPCM5	1,00E-54	LinJ.32.4020	Myo13 (TCDM_05821)
	5,00E-38	LinJ.34.1070	Myo1 (TCDM_07314)
	3,00E-19	LinJ.25.0490	MyoG (TCDM_02016)
Leishmania major strain Friedlin	2,00E-53	LmjF.32.3870	Myo13 (TCDM_05821)
	3,00E-39	LmjF.34.1000	Myo1 (TCDM_07314)
	8,00E-18	LmjF.25.0480	MyoG (TCDM_02016)
Leishmania major strain LV39c5	2,00E-53	LMJLV39_320046600	Myo13 (TCDM_05821)
	5,00E-38	LMJLV39_340016900	Myo1 (TCDM_07314)
	6,00E-18	LMJLV39_250010400	MyoG (TCDM_02016)
Leishmania major strain SD 75.1	8,00E-53	MJSD75_320046800	Myo13 (TCDM_05821)
	5,00E-38	LMJSD75_340016900	Myo1 (TCDM_07314)
	8,00E-18	LMJSD75_250010400	MyoG (TCDM_02016)
Leishmania mexicana MHOM/GT/2001/U1103	5,00E-52	LmxM.31.3870	Myo13 (TCDM_05821)
	1,00E-40	LmxM.33.1000	Myo1 (TCDM_07314)
	6,00E-23	LmxM.25.0480	MyoG (TCDM_02016)
Leishmania panamensis MHOM/COL/81/L13	2,00E-53	LPAL13_320047300	Myo13 (TCDM_05821)
	1,00E-25	LPAL13_200014600	Myo1 (TCDM_07314)

	1,00E-15	LPAL13_250009400	MyoG (TCDM_02016)
Leishmania tarentolae Parrot-TarII	8,00E-55	LtaP32.4040	Myo13 (TCDM_05821)
	9,00E-42	LtaP34.1150	Myo1 (TCDM_07314)
	1,00E-20	LtaP25.0510	MyoG (TCDM_02016)
Leishmania tropica L590	1,00E-54	LTRL590_320046900	Myo13 (TCDM_05821)
	8,00E-40	LTRL590_340015400	Myo1 (TCDM_07314)
	9,00E-19	LTRL590_250010700	MyoG (TCDM_02016)
Leishmania turanica strain LEM423	5,00E-53	LTULEM423_320047500	Myo13 (TCDM_05821)
	1,00E-38	LTULEM423_000006800	Myo1 (TCDM_07314)
	1,00E-17	LTULEM423_250010400	MyoG (TCDM_02016)
Leishmania sp. MAR LEM2494	2,00E-49	LMARLEM2494_320047400	Myo13 (TCDM_05821)
	1,00E-22	LMARLEM2494_340015700	Myo1 (TCDM_07314)
	1,00E-10	LMARLEM2494_250010200	MyoG (TCDM_02016)
Leptomonas pyrrhocoris	5,00E-55	LpyrH10_02_4330	Myo13 (TCDM_05821)
	2,00E-23	LpyrH10_05_1440	Myo1 (TCDM_07314)
	1,00E-14	LpyrH10_10_0550	MyoG (TCDM_02016)
Leptomonas seymouri ATCC 30220	5,00E-54	Lsey_0246_0030	Myo13 (TCDM_05821)
	3,00E-25	Lsey_0435_0030	Myo1 (TCDM_07314)
	2,00E-14	Lsey_0027_0010	MyoG (TCDM_02016)
Trypanosoma brucei Lister strain 427	2,00E-50	Tb427tmp.01.7990	Myo13 (TCDM_05821)
	2,00E-40	Tb427.04.3380	Myo1 (TCDM_07314)
Trypanosoma brucei brucei TREU927	2,00E-51	Tb927.11.16310	Myo13 (TCDM_05821)
	5,00E-41	Tb927.4.3380	Myo1 (TCDM_07314)
Trypanosoma brucei gambiense DAL972	3,00E-50	Tbg972.11.18310	Myo13 (TCDM_05821)
	1,00E-40	Tbg972.4.3390	Myo1 (TCDM_07314)
Trypanosoma congolense IL3000	1,00E-42	TcIL3000_4_3080	Myo1 (TCDM_07314)
Trypanosoma cruzi marinkellei strain B7	0.0	Tc_MARK_4094	MyoG (TCDM_02016)
	1,00E-54	Tc_MARK_3528	MyoC (TCDM_02877)
	2,00E-54	Tc_MARK_594	MyoB (TCDM_07433)
	1,00E-52	Tc_MARK_4039	Myo13 (TCDM_05821)
	3,00E-50	Tc_MARK_3325	MyoA (TCDM_09957)
	7,00E-45	Tc_MARK_8887	MyoE (TCDM_06166)
	6,00E-44	Tc_MARK_845	MyoD (TCDM_07686)
	4,00E-42	Tc_MARK_4644	MyoF (TCDM_08875)
	5,00E-09	Tc_MARK_2656	Myo1 (TCDM_07314)
Trypanosoma evansi strain STIB 805	4,00E-51	TevSTIB805.11_01.16880	Myo13 (TCDM_05821)
	5,00E-41	TevSTIB805.4.3470	Myo1 (TCDM_07314)
Trypanosoma grayi ANR4	0.0	DQ04_03771000	MyoG (TCDM_02016)
	5,00E-55	DQ04_02991050	MyoC (TCDM_02877)
	2,00E-53	DQ04_01671000	MyoB (TCDM_07433)
	5,00E-53	DQ04_00031100	Myo13 (TCDM_05821)
	5,00E-53	DQ04_00821150	MyoA (TCDM_09957)
	2,00E-51	DQ04_03341050	MyoF (TCDM_08875)
	4,00E-47	DQ04_01311010	MyoE (TCDM_06166)
	3,00E-44	DQ04_00501100	Myo1 (TCDM_07314)
	2,00E-41	DQ04_01331060	MyoD (TCDM_07686)
	6,00E-04	DQ04_00271170	MyoH (TCDM_02145)
Trypanosoma rangeli SC58	1,00E-41	TRSC58_06141	MyoD (TCDM_07686)
	4,00E-39	TRSC58_00243	MyoE (TCDM_06166)
	2,00E-23	TRSC58_01895	MyoF (TCDM_08875)

Trypanosoma vivax Y486	2,00E-51	TvY486_1117220	Myo13 (TCDM_05821)
	3,00E-44	TvY486_0403180	Myo1 (TCDM_07314)
NCBI			
Genomes	**BlastP e-value**	**ID of Hit**	**Reciprocal Blast**
Green boxes are hits that correspond to MyoG/MyoG-derived genes			
Kinetoplastids			
Trypanosoma theileri	0.0	ORC91827.1	MyoG (TCDM_02016)
	1,00E-54	ORC88651.1	MyoB (TCDM_07433)
	2,00E-53	ORC93297.1	MyoA (TCDM_09957)
	2,00E-50	ORC87480.1	Myo13 (TCDM_05821)
	4,00E-50	ORC90398.1	MyoC (TCDM_02877)
	2,00E-49	ORC89982.1	MyoF (TCDM_08875)
	3,00E-45	ORC86814.1	MyoD (TCDM_07686)
	4,00E-44	ORC93509.1	Myo1 (TCDM_07314)
	9,00E-44	ORC86402.1	MyoE (TCDM_06166)
Angomonas deanei	2,00E-54	EPY29051.1	Myo13 (TCDM_05821)
	1,00E-53	EPY42972.1	Myo13 (TCDM_05821)
	3,00E-18	EPY31408.1	Myo1 (TCDM_07314)
	8,00E-04	EPY24057.1	MyoG (TCDM_02016)
Strigomonas culicis	2,00E-53	EPY36094.1	Myo13 (TCDM_05821)
	3,00E-53	EPY22327.1	Myo13 (TCDM_05821)
	4,00E-16	EPY17541.1	Myo1 (TCDM_07314)
Phytomonas sp. isolate EM1	2,00E-55	CCW60276.1	Myo13 (TCDM_05821)
Phytomonas sp. isolate Hart1	8,00E-53	CCW68456.1	Myo13 (TCDM_05821)
Bodo saltans	8,00E-134	CUG86977.1	MyoG (TCDM_02016)
	8,00E-59	CUG90281.1	MyoB (TCDM_07433)
	4,00E-54	CUG92185.1	MyoB (TCDM_07433)
	6,00E-54	CUF67578.1	MyoB (TCDM_07433)
	3,00E-53	CUG89451.1	MyoA (TCDM_09957)
	9,00E-53	CUG03103.1	Myo13 (TCDM_05821)
	2,00E-51	CUG91579.1	MyoF (TCDM_08875)
	3,00E-48	CUG87167.1	MyoB (TCDM_07433)
	8,00E-47	CUF99905.1	MyoE (TCDM_06166)
	3,00E-43	CUG93737.1	MyoB (TCDM_07433)
	1,00E-42	CUI15073.1	Myo1 (TCDM_07314)
	3,00E-36	CUG03397.1	MyoC (TCDM_02877)
	2,00E-31	CUF17710.1	MyoD (TCDM_07686)
Examples of BlastP result against non-kinetoplastid species			
Aplysia californica	1,00E-70	XP_012946756.1	MyoC (TCDM_02877)
Sinocyclocheilus rhinocerous	1,00E-64	XP_016407805.1	MyoA (TCDM_09957)
Oncorhynchus mykiss	3,00E-63	CDQ77593.1	MyoA (TCDM_09957)
Rhagoletis zephyria	1,00E-62	XP_017489243.1	Myo1 (TCDM_07314)
Copidosoma floridanum	1,00E-62	XP_014216723.1	Myo1 (TCDM_07314)
Drosophila busckii	2,00E-62	XP_017850026.1	Myo1 (TCDM_07314)
Tyto alba	2,00E-62	KFV50702.1	MyoA (TCDM_09957)
Drosophila virilis	1,00E-61	XP_002058185.2	Myo1 (TCDM_07314)
Oncorhynchus kisutch	1,00E-61	XP_020321390.1	MyoA (TCDM_09957)
Aspergillus oryzae	6,00E-61	OOO03948.1	MyoA (TCDM_09957)

Table S6: Synteny conservation analysis of MyoG gene

Genome	Upstream gene	MyoG/MyoG derived gene	Downstream gene 1	Downstream gene 4
**Green boxes highlight the species that have MyoG/MyoG derived gene in conserved syntenic region in relation to *T. cruzi* MyoG**				
Blechomonas ayalai B08-376	Baya_001_1420	no remnants	Baya_001_1410	Baya_001_1380
Crithidia fasciculata strain Cf-Cl	CFAC1_220012300	CFAC1_220012200	CFAC1_220012100	CFAC1_220011800
Endotrypanum monterogeii strain LV88	EMOLV88_250010100	EMOLV88_250010000	EMOLV88_250009900	EMOLV88_250009600
Leishmania aethiopica L147	LAEL147_000404700	LAEL147_000404600	LAEL147_000404500	LAEL147_000404300
Leishmania arabica strain LEM1108	LARLEM1108_250010300	LARLEM1108_250010200	LARLEM1108_250010100	LARLEM1108_250009900
Leishmania braziliensis MHOM/BR/75/M2903	LBRM2903_250010600	LBRM2903_250010500	LBRM2903_250010400	LBRM2903_250009900
Leishmania braziliensis MHOM/BR/75/M2904	LbrM.25.0440	LbrM.25.0430	LbrM.25.0420	LbrM.25.0400
Leishmania donovani BPK282A1	LdBPK_250500.1	LdBPK_250490.1	LdBPK_250480.1	LdBPK_250460.1
Leishmania enriettii strain LEM3045	LENLEM3045_250010100	LENLEM3045_250010000	LENLEM3045_250009900	LENLEM3045_250009500
Leishmania gerbilli strain LEM452	LGELEM452_250010300	LGELEM452_250010200	LGELEM452_250010100	LGELEM452_250009900
Leishmania infantum JPCM5	LinJ.25.0500	LinJ.25.0490	LinJ.25.0480	LinJ.25.0460
Leishmania major strain Friedlin	LmjF.25.0490	LmjF.25.0480	LmjF.25.0470	LmjF.25.0450
Leishmania major strain LV39c5	LMJLV39_250010500	LMJLV39_250010400	LMJLV39_250010300	LMJLV39_250010100
Leishmania major strain SD 75.1	LMJSD75_250010500	LMJSD75_250010400	LMJSD75_250010300	LMJSD75_250010100
Leishmania mexicana MHOM/GT/2001/U1103	LmxM.25.0490	LmxM.25.0480	LmxM.25.0470	LmxM.25.0450
Leishmania panamensis MHOM/COL/81/L13	LPAL13_250009500	LPAL13_250009400	LPAL13_250009300	LPAL13_250009000
Leishmania tarentolae Parrot-TarII	LtaP25.0520	LtaP25.0510	LtaP25.0500	LtaP25.0480
Leishmania tropica L590	LTRL590_250010800	LTRL590_250010700	LTRL590_250010600	LTRL590_250010400
Leishmania turanica strain LEM423	LTULEM423_250010500	LTULEM423_250010400	LTULEM423_250010300	LTULEM423_250010100
Leishmania sp. MAR LEM2494	LMARLEM2494_250010300	LMARLEM2494_250010200	LMARLEM2494_250010100	LARLEM1108_250009900
Leptomonas pyrrhocoris	LpyrH10_10_0560	LpyrH10_10_0550	LpyrH10_10_0540	LpyrH10_10_0510
Leptomonas seymouri ATCC 30220	End of contig	Lsey_0027_0010	Lsey_0027_0020	Lsey_0027_0050
Trypanosoma brucei Lister strain 427	Tb427tmp.03.0620	no remnants	no homologous found	Tb427tmp.03.0660
Trypanosoma brucei brucei TREU927	Tb927.11.500	no remnants	no homologous found	Tb927.11.480
Trypanosoma brucei gambiense DAL972	Tbg972.11.450	no remnants	no homologous found	Tbg972.11.390
Trypanosoma congolense IL3000	TcIL3000.11.470	no remnants	no homologous found	TcIL3000.11.420
Trypanosoma cruzi CL Brener Esmeraldo-like	TcCLB.507093.220	TcCLB.507093.210	TcCLB.507093.200	TcCLB.507093.184
Trypanosoma cruzi Dm28c	TCDM_02013	TCDM_02016	TCDM_02017	TCDM_02019
Trypanosoma cruzi marinkellei strain B7	Tc_MARK_4095	Tc_MARK_4094	Tc_MARK_4093	Tc_MARK_4091
Trypanosoma evansi strain STIB 805	TevSTIB805.11_01.440	no remnants	no homologous found	TevSTIB805.11_01.420
Trypanosoma grayi ANR4	End of contig	DQ04_03771000	DQ04_03771010	DQ04_03771050
*Trypanosoma rangeli SC58**	TRSC58_07088	no orthologous found	TRSC58_01401	TRSC58_02844
Trypanosoma vivax Y486	TvY486_110035	missing region	missing region	TvY486_1100340
Trypanosoma theileri isolate Edinburgh TM35_Tth_5_V1#	NBCO01000005.1:862846-863241	NBCO01000005.1:867588-871364	NBCO01000005.1:872052-875036	na
Bodo saltans#	CYKH01000248.1:36268-36537	CYKH01001420.1:470-3592	CYKH01002159.1:41638-44229	na
Angomonas deanei strain ATCC PRA-265#	KV452486.1:145652-145924	KV452486.1:147630-150329	KV452486.1:150715-153603	na
Strigomonas culicis strain TCC012E#	AUXH01000083.1:2744-3022	no orthologous found	AUXH01000032.1:52 -1106	na
Phytomas sp EM1#	HF955074.1:266051-266305	no orthologous found	no homologous found	na
Phytomas sp Hart1#	HF955208.1:252906-253175	no orthologous found	no homologous found	na

The IDs correspond to the homologous of *T. cruzi* Dm28c upstream or downstream genes. In some cases, they are not the nearest gene in the analyzed species.
* Evaluation of synteny for this species was not possible since the genes were located in very small contigs.
\# For these species, the approximate chromosome position is presented. Genomes obtained from NCBI.
no orthologous found - we were not able to analyze the syntenic region and no orthologous gene was found throughout the genome
no remnants- we were able to analyze the syntenic region and no consistent signals of the presence of gene was found
na- not analyzed
missing region - genomic region that is filled with N

Table S7: Summary of BlastP results for Myo13 against sequences available at TritrypDB and NCBI

TritrypDB			
**Genomes**	**BlastP e-value**	**ID of Hit**	**Reciprocal Blast/Synteny**
**Green boxes are the hits that correspond to Myo13 genes**			
Blechomonas ayalai B08-376	0.0	Baya_099_0300	Myo13 (TCDM_05821)
	1,00E-79	Baya_227_0080	Myo1 (TCDM_07314)
Crithidia fasciculata strain Cf-Cl	0.0	CFAC1_300048900	Myo13 (TCDM_05821)
	9,00E-69	CFAC1_290040800	Myo1 (TCDM_07314)
Endotrypanum monterogeii strain LV88	0.0	EMOLV88_320042600	Myo13 (TCDM_05821)
	5,00E-37	EMOLV88_340015100	Myo1 (TCDM_07314)
Leishmania aethiopica L147	0.0	LAEL147_000662900.1	Myo13 (TCDM_05821)
	3,00E-35	LAEL147_000713700	Myo1 (TCDM_07314)
Leishmania arabica strain LEM1108	0.0	LARLEM1108_320047000	Myo13 (TCDM_05821)
	4,00E-36	LARLEM1108_000017500	Myo1 (TCDM_07314)
Leishmania braziliensis MHOM/BR/75/M2903	0.0	LBRM2903_320050900	Myo13 (TCDM_05821)
	5,00E-36	LBRM2903_200016800	Myo1 (TCDM_07314)
	3,00E-36	LBRM2903_320050800	Myo13 (TCDM_05821)
Leishmania braziliensis MHOM/BR/75/M2904	0.0	LbrM.32.4110	Myo13 (TCDM_05821)
	3,00E-36	LbrM.20.0970	Myo1 (TCDM_07314)
Leishmania donovani BPK282A1	0.0	LdBPK_324020.1	Myo13 (TCDM_05821)
	3,00E-35	LdBPK_341070.1	Myo1 (TCDM_07314)
Leishmania enriettii strain LEM3045	0.0	LENLEM3045_320047100	Myo13 (TCDM_05821)
	4,00E-35	LENLEM3045_340014600	Myo1 (TCDM_07314)
Leishmania gerbilli strain LEM452	0.0	LGELEM452_320047000	Myo13 (TCDM_05821)
	3,00E-36	LGELEM452_000011900	Myo1 (TCDM_07314)
Leishmania infantum JPCM5	0.0	LinJ.32.4020	Myo13 (TCDM_05821)
	3,00E-35	LinJ.34.1070	Myo1 (TCDM_07314)
Leishmania major strain Friedlin	0.0	LmjF.32.3870	Myo13 (TCDM_05821)
	5,00E-35	LmjF.34.1000	Myo1 (TCDM_07314)
Leishmania major strain LV39c5	0.0	LMJLV39_320046600	Myo13 (TCDM_05821)
	5,00E-35	LMJLV39_340016900	Myo1 (TCDM_07314)
Leishmania major strain SD 75.1	0.0	LMJSD75_320046800	Myo13 (TCDM_05821)
	5,00E-35	LMJSD75_340016900	Myo1 (TCDM_07314)
Leishmania mexicana MHOM/GT/2001/U1103	0.0	LmxM.31.3870	Myo13 (TCDM_05821)
	9,00E-36	LmxM.33.1000	Myo1 (TCDM_07314)
Leishmania panamensis MHOM/COL/81/L13	0.0	LPAL13_320047300	Myo13 (TCDM_05821)
	8,00E-37	LPAL13_200014600	Myo1 (TCDM_07314)
Leishmania tarentolae Parrot-TarII	0.0	LtaP32.4040	Myo13 (TCDM_05821)
	4,00E-35	LtaP34.1150	Myo1 (TCDM_07314)
Leishmania tropica L590	0.0	LTRL590_320046900	Myo13 (TCDM_05821)
	2,00E-35	LTRL590_340015400	Myo1 (TCDM_07314)
Leishmania turanica strain LEM423	0.0	LTULEM423_320047500	Myo13 (TCDM_05821)
	6,00E-69	LTULEM423_000006800	Myo1 (TCDM_07314)
Leishmania sp. MAR LEM2494	0.0	LMARLEM2494_320047400	Myo13 (TCDM_05821)

	1,00E-36	LMARLEM2494_340015700	Myo1 (TCDM_07314)
Leptomonas pyrrhocoris	0.0	LpyrH10_02_4330	Myo13 (TCDM_05821)
	4,00E-68	LpyrH10_05_1440	Myo1 (TCDM_07314)
Leptomonas seymouri ATCC 30220	0.0	Lsey_0246_0030	Myo13 (TCDM_05821)
	8,00E-34	Lsey_0435_0030	Myo1 (TCDM_07314)
Trypanosoma brucei Lister strain 427	0.0	Tb427tmp.01.7990	Myo13 (TCDM_05821)
	9,00E-74	Tb427.04.3380	Myo1 (TCDM_07314)
Trypanosoma brucei brucei TREU927	0.0	Tb927.11.16310	Myo13 (TCDM_05821)
	3,00E-74	Tb927.4.3380	Myo1 (TCDM_07314)
Trypanosoma brucei gambiense DAL972	0.0	Tbg972.11.18310	Myo13 (TCDM_05821)
	1,00E-73	Tbg972.4.3390	Myo1 (TCDM_07314)
Trypanosoma congolense IL3000	9,00E-73	TcIL3000_4_3080	Myo1 (TCDM_07314)
Trypanosoma cruzi marinkellei strain B7	0.0	Tc_MARK_4039	Myo13 (TCDM_05821)
	7,00E-120	Tc_MARK_3325	MyoA (TCDM_09957)
	7,00E-117	Tc_MARK_594	MyoB (TCDM_07433)
	3,00E-116	Tc_MARK_3528	MyoC (TCDM_02877)
	2,00E-113	Tc_MARK_8887	MyoE (TCDM_06166)
	3,00E-95	Tc_MARK_845	MyoD (TCDM_07686)
	2,00E-92	Tc_MARK_4644	MyoF (TCDM_08875)
	3,00E-52	Tc_MARK_4094	MyoG (TCDM_02016)
	6,00E-21	Tc_MARK_2656	Myo1 (TCDM_07314)
	6,00E-07	Tc_MARK_2031	MyoH-derived (TCDM_02145)
Trypanosoma evansi strain STIB 805	0.0	TevSTIB805.11_01.16880	Myo13 (TCDM_05821)
	3,00E-74	TevSTIB805.4.3470	Myo1 (TCDM_07314)
Trypanosoma grayi ANR4	0.0	DQ04_00031100	Myo13 (TCDM_05821)
	3,00E-123	DQ04_02991050	MyoC (TCDM_02877)
	3,00E-123	DQ04_01671000	MyoB (TCDM_07433)
	3,00E-117	DQ04_01311010	MyoE (TCDM_06166)
	1,00E-116	DQ04_00821150	MyoA (TCDM_09957)
	2,00E-100	DQ04_03341050	MyoF (TCDM_08875)
	4,00E-88	DQ04_01331060	MyoD (TCDM_07686)
	4,00E-79	DQ04_00501100	Myo1 (TCDM_07314)
	4,00E-52	DQ04_03771000	MyoG (TCDM_02016)
	0.004	DQ04_00271170	MyoH-derived (TCDM_02145)
Trypanosoma rangeli SC58	5,00E-109	TRSC58_00243	MyoE (TCDM_06166)
	2,00E-95	TRSC58_06141	MyoD (TCDM_07686)
	5,00E-52	TRSC58_01895	MyoF (TCDM_08875)
	6,00E-10	TRSC58_00411	MyoB (TCDM_07433)
	3,00E-06	TRSC58_00453	MyoH-derived (TCDM_02145)
Trypanosoma vivax Y486	0.0	TvY486_1117220	Myo13 (TCDM_05821)
	1,00E-81	TvY486_0403180	Myo1 (TCDM_07314)

**NCBI**			
**Genomes**	**BlastP e-value**	**ID of Hit**	**Reciprocal Blast**
**Green boxes are hits that correspond to Myo13**			
**Kinetoplastids**			
Trypanosoma theileri	0.0	ORC87480.1	Myo13 (TCDM_05821)
	7,00E-123	ORC90398.1	MyoC (TCDM_02877)
	2,00E-114	ORC93297.1	MyoA (TCDM_09957)
	6,00E-114	ORC88651.1	MyoB (TCDM_07433)
	1,00E-111	ORC86402.1	MyoE (TCDM_06166)
	7,00E-99	ORC89982.1	MyoF (TCDM_08875)
	8,00E-97	ORC86814.1	MyoD (TCDM_07686)
	2,00E-76	ORC93509.1	Myo1 (TCDM_07314)

	2,00E-55	ORC91827.1	MyoG (TCDM_02016)
Angomonas deanei	0.0	EPY36601.1	Myo13 (TCDM_05821)
	0.0	EPY29051.1	Myo13 (TCDM_05821)
	0.0	EPY42972.1	Myo13 (TCDM_05821)
	1,00E-68	EPY31408.1	Myo1 (TCDM_07314)
Strigomonas culicis	0.0	EPY22327.1	Myo13 (TCDM_05821)
	0.0	EPY36094.1	Myo13 (TCDM_05821)
	4,00E-36	EPY17541.1	Myo1 (TCDM_07314)
	1,00E-20	EPY17540.1	Myo1 (TCDM_07314)
Phytomonas sp. isolate EM1	0.0	CCW60276.1	Myo13 (TCDM_05821)
Phytomonas sp. isolate Hart1	0.0	CCW68456.1	Myo13 (TCDM_05821)
Bodo saltans	0.0	CUG03103.1	Myo13 (TCDM_05821)
	1,00E-121	CUF67578.1	MyoB (TCDM_07433)
	3,00E-116	CUG89451.1	MyoA (TCDM_09957)
	2,00E-113	CUF99905.1	MyoE (TCDM_06166)
	5,00E-112	CUG90281.1	MyoB (TCDM_07433)
	5,00E-112	CUG87167.1	MyoB (TCDM_07433)
	4,00E-111	CUG93737.1	MyoB (TCDM_07433)
	2,00E-102	CUG92185.1	MyoB (TCDM_07433)
	3,00E-101	CUG91579.1	MyoF (TCDM_08875)
	7,00E-94	CUG03397.1	MyoC (TCDM_02877)
	6,00E-87	CUI15073.1	Myo1 (TCDM_07314)
	1,00E-49	CUG86977.1	MyoG (TCDM_02016)
	4,00E-46	CUF17710.1	MyoD (TCDM_07686)
**Examples of BlastP result against non-kinetoplastid species**			
Naegleria gruberi	9,00E-138	XP_002681567.1	MyoB (TCDM_07433)
Naegleria gruberi	1,00E-126	XP_002680898.1	MyoC (TCDM_02877)
Saprolegnia parasitica	8,00E-124	XP_012197412.1	MyoB (TCDM_07433)
Ricinus communis	6,00E-117	XP_015575858.1	MyoA (TCDM_09957)
Dictyostelium lacteum	2,00E-119	KYQ99898.1	Myo1 (TCDM_07314)
Physcomitrella patens	2,00E-119	ADG63228.1	MyoA (TCDM_09957)
Kazachstania africana	6E-119	XP_003959550.1	MyoA (TCDM_09957)
Nematostella vectensis	3E-117	XP_001637626.1	Myo1 (TCDM_07314)
Thalassiosira pseudonana	4,00E-117	XP_002294196.1	MyoC (TCDM_02877)
Acytostelium subglobosum	7,00E-117	XP_012748661.1	Myo1 (TCDM_07314)

Table S8: Synteny conservation analysis of Myo13 gene

Genome	Upstream gene	Myo13 derived gene	Downstream gene
**Green boxes highlight the species that have Myo13 gene in conserved syntenic region in relation to *T. cruzi* Myo13**			
Blechomonas ayalai B08-376	Baya_099_0290	Baya_099_0300	Baya_099_0310
Crithidia fasciculata strain Cf-Cl	CFAC1_300049000	CFAC1_300048900	CFAC1_300048800
Endotrypanum monterogeii strain LV88	EMOLV88_320042500	EMOLV88_320042600	EMOLV88_320042700
Leishmania aethiopica L147	LAEL147_000662800	LAEL147_000662900	LAEL147_000663000
Leishmania arabica strain LEM1108	LARLEM1108_320046900	LARLEM1108_320047000	LARLEM1108_320047100
Leishmania braziliensis MHOM/BR/75/M2903	LBRM2903_320050700	LBRM2903_320050800	LBRM2903_320051000
Leishmania braziliensis MHOM/BR/75/M2904	LbrM.32.4100	LbrM.32.4110	LbrM.32.4120
Leishmania donovani BPK282A1	LdBPK_324010.1	LdBPK_324020.1	LdBPK_324030.1
Leishmania enriettii strain LEM3045	LENLEM3045_320047000	LENLEM3045_320047100	LENLEM3045_320047200
Leishmania gerbilli strain LEM452	LGELEM452_320046900	LGELEM452_320047000	LGELEM452_320047100
Leishmania infantum JPCM5	LinJ.32.4010	LinJ.32.4020	LinJ.32.4030
Leishmania major strain Friedlin	LmjF.32.3860	LmjF.32.3870	LmjF.32.3880
Leishmania major strain LV39c5	LMJLV39_320046500	LMJLV39_320046600	LMJLV39_320046700
Leishmania major strain SD 75.1	LMJSD75_320046700	LMJSD75_320046800	LMJSD75_320046900
Leishmania mexicana MHOM/GT/2001/U1103	LmxM.31.3860	LmxM.31.3870	LmxM.31.3880
Leishmania panamensis MHOM/COL/81/L13	LPAL13_320047200	LPAL13_320047300	LPAL13_320047400
Leishmania tarentolae Parrot-TarII	LtaP32.4030	LtaP32.4040	LtaP32.4050
Leishmania tropica L590	LTRL590_320046800	LTRL590_320046900	LTRL590_320047000
Leishmania turanica strain LEM423	LTULEM423_320047400	LTULEM423_320047500	LTULEM423_320047600
Leishmania sp. MAR LEM2494	LMARLEM2494_320047300	LMARLEM2494_320047400	LMARLEM2494_320047500
Leptomonas pyrrhocoris	LpyrH10_02_4320	LpyrH10_02_4330	LpyrH10_02_4340
Leptomonas seymouri ATCC 30220	Lsey_0246_0040	Lsey_0246_0030	Lsey_0246_0020
Trypanosoma brucei Lister strain 427	Tb427tmp.01.8010	Tb427tmp.01.7990	Tb427tmp.01.7980
Trypanosoma brucei brucei TREU927	Tb927.11.16330	Tb927.11.16310	Tb927.11.16300
Trypanosoma brucei gambiense DAL972	Tbg972.11.18330	Tbg972.11.18310	Tbg972.11.18300
Trypanosoma congolense IL3000	TcIL3000.11.16230	no remnants	TcIL3000.11.16210
Trypanosoma cruzi CL Brener Esmeraldo-like	TcCLB.511527.82	TcCLB.511527.70	TcCLB.511527.60
Trypanosoma cruzi Dm28c	TCDM_05822	TCDM_05821	TCDM_05818
Trypanosoma cruzi marinkellei strain B7	Tc_MARK_4038	Tc_MARK_4039	Tc_MARK_4040
Trypanosoma evansi strain STIB 805	TevSTIB805.11_01.16900	TevSTIB805.11_01.16880	TevSTIB805.11_01.16870
Trypanosoma grayi ANR4	DQ04_00031120	DQ04_00031100	DQ04_00031090
*Trypanosoma rangeli SC58**	TRSC58_03196*	no orthologous found	TRSC58_06016*
Trypanosoma vivax Y486	TvY486_1117240	TvY486_1117220	TvY486_1117210
Trypanosoma theileri isolate Edinburgh TM35_Tth_5_V1#	NBCO01000022.1:413421-416201	NBCO01000022.1:417782-420943	NBCO01000022.1:422018-422917
Bodo saltans#	CYKH01000505.1:6892-8241	CYKH01000505.1:12443-15655	no homologous found
Angomonas deanei strain ATCC PRA-265#	KV452506.1:160255-162942	KV452506.1:163568-166711	KV452506.1:167512-168375
Strigomonas culicis strain TCC012E#	AUXH01000061.1:18272-21277	AUXH01000061.1:22462-25614	AUXH01000061.1:27318-28208

The IDs correspond to the homologous of *T. cruzi* Dm28c upstream or downstream genes. In some cases, they are not the nearest gene in the analyzed species.
* Evaluation of synteny for this species was not possible since the genes were located in very small contigs.
# For these species, the approximate chromosome position is presented. Genomes obtained from NCBI.
no orthologous found - we were not able to analyze the syntenic region and no orthologous gene was found throughout the genome
no remnants- we were able to analyze the syntenic region and no consistent signals of the presence of gene was found

Table S9: Summary of BlastP results for MyoA against sequences available at TritrypDB and NCBI

TritrypDB			
**Genomes**	**BlastP e-value**	**ID of Hit**	**Reciprocal Blast/Synteny**
**Green boxes are the hits that correspond to MyoA genes**			
Blechomonas ayalai B08-376	2,00E-119	Baya_099_0300	Myo13 (TCDM_05821)
	1,00E-96	Baya_227_0080	Myo1 (TCDM_07314)
Crithidia fasciculata strain Cf-Cl	2,00E-119	CFAC1_300048900	Myo13 (TCDM_05821)
	6,00E-50	CFAC1_290040800	Myo1 (TCDM_07314)
Endotrypanum monterogeii strain LV88	1,00E-120	EMOLV88_320042600	Myo13 (TCDM_05821)
	7,00E-54	EMOLV88_340015100	Myo1 (TCDM_07314)
Leishmania aethiopica L147	5,00E-124	LAEL147_000662900.1	Myo13 (TCDM_05821)
	6,00E-51	LAEL147_000713700	Myo1 (TCDM_07314)
Leishmania arabica strain LEM1108	1,00E-121	LARLEM1108_320047000	Myo13 (TCDM_05821)
	1,00E-50	LARLEM1108_000017500	Myo1 (TCDM_07314)
Leishmania braziliensis MHOM/BR/75/M2903	4,00E-93	LBRM2903_320050900	Myo13 (TCDM_05821)
	5,00E-49	LBRM2903_200016800	Myo1 (TCDM_07314)
Leishmania braziliensis MHOM/BR/75/M2904	2,00E-116	LbrM.32.4110	Myo13 (TCDM_05821)
	2,00E-48	LbrM.20.0970	Myo1 (TCDM_07314)
Leishmania donovani BPK282A1	1,00E-126	LdBPK_324020.1	Myo13 (TCDM_05821)
	8,00E-51	LdBPK_341070.1	Myo1 (TCDM_07314)
Leishmania enriettii strain LEM3045	4,00E-120	LENLEM3045_320047100	Myo13 (TCDM_05821)
	6,00E-50	LENLEM3045_340014600	Myo1 (TCDM_07314)
Leishmania gerbilli strain LEM452	1,00E-123	LGELEM452_320047000	Myo13 (TCDM_05821)
	2,00E-50	LGELEM452_000011900	Myo1 (TCDM_07314)
Leishmania infantum JPCM5	7,00E-127	LinJ.32.4020	Myo13 (TCDM_05821)
	1,00E-50	LinJ.34.1070	Myo1 (TCDM_07314)
Leishmania major strain Friedlin	6,00E-124	LmjF.32.3870	Myo13 (TCDM_05821)
	1,00E-49	LmjF.34.1000	Myo1 (TCDM_07314)
Leishmania major strain LV39c5	3,00E-123	LMJLV39_320046600	Myo13 (TCDM_05821)
	2,00E-49	LMJLV39_340016900	Myo1 (TCDM_07314)
Leishmania major strain SD 75.1	2,00E-124	MJSD75_320046800	Myo13 (TCDM_05821)
	1,00E-49	LMJSD75_340016900	Myo1 (TCDM_07314)
Leishmania mexicana MHOM/GT/2001/U1103	4,00E-126	LmxM.31.3870	Myo13 (TCDM_05821)
	2,00E-51	LmxM.33.1000	Myo1 (TCDM_07314)
Leishmania panamensis MHOM/COL/81/L13	7,00E-116	LPAL13_320047300	Myo13 (TCDM_05821)
	1,00E-47	LPAL13_200014600	Myo1 (TCDM_07314)
Leishmania tarentolae Parrot-TarII	1,00E-124	LtaP32.4040	Myo13 (TCDM_05821)
	2,00E-51	LtaP34.1150	Myo1 (TCDM_07314)
Leishmania tropica L590	2,00E-125	LTRL590_320046900	Myo13 (TCDM_05821)
	5,00E-51	LTRL590_340015400	Myo1 (TCDM_07314)
Leishmania turanica strain LEM423	1,00E-123	LTULEM423_320047500	Myo13 (TCDM_05821)
	1,00E-50	LTULEM423_000006800	Myo1 (TCDM_07314)
Leishmania sp. MAR LEM2494	1,00E-123	LMARLEM2494_320047400	Myo13 (TCDM_05821)
	1,00E-51	LMARLEM2494_340015700	Myo1 (TCDM_07314)

Leptomonas pyrrhocoris	2,00E-124	LpyrH10_02_4330	Myo13 (TCDM_05821)
	3,00E-49	LpyrH10_05_1440	Myo1 (TCDM_07314)
Leptomonas seymouri ATCC 30220	2,00E-122	Lsey_0246_0030	Myo13 (TCDM_05821)
	2,00E-49	Lsey_0435_0030	Myo1 (TCDM_07314)
Trypanosoma brucei Lister strain 427	2,00E-118	Tb427tmp.01.7990	Myo13 (TCDM_05821)
	3,00E-100	Tb427.04.3380	Myo1 (TCDM_07314)
Trypanosoma brucei brucei TREU927	2,00E-119	Tb927.11.16310	Myo13 (TCDM_05821)
	1,00E-99	Tb927.4.3380	Myo1 (TCDM_07314)
Trypanosoma brucei gambiense DAL972	2,00E-117	Tbg972.11.18310	Myo13 (TCDM_05821)
	3,00E-100	Tbg972.4.3390	Myo1 (TCDM_07314)
Trypanosoma congolense IL3000	6,00E-105	TcIL3000_4_3080	Myo1 (TCDM_07314)
Trypanosoma cruzi marinkellei strain B7	0.0	Tc_MARK_3325	MyoA (TCDM_09957)
	0.0	Tc_MARK_3528	MyoC (TCDM_02877)
	0.0	Tc_MARK_594	MyoB (TCDM_07433)
	0.0	Tc_MARK_8887	MyoE (TCDM_06166)
	1,00E-141	Tc_MARK_4644	MyoF (TCDM_08875)
	3,00E-140	Tc_MARK_845	MyoD (TCDM_07686)
	2,00E-121	Tc_MARK_4039	Myo13 (TCDM_05821)
	2,00E-50	Tc_MARK_4094	MyoG (TCDM_02016)
	6,00E-35	Tc_MARK_2656	Myo1 (TCDM_07314)
Trypanosoma evansi strain STIB 805	8,00E-118	TevSTIB805.11_01.16880	Myo13 (TCDM_05821)
	7,00E-101	TevSTIB805.4.3470	Myo1 (TCDM_07314)
Trypanosoma grayi ANR4	0.0	DQ04_00821150	MyoA (TCDM_09957)
	0.0	DQ04_02991050	MyoC (TCDM_02877)
	0.0	DQ04_01671000	MyoB (TCDM_07433)
	0.0	DQ04_01311010	MyoE (TCDM_06166)
	2,00E-161	DQ04_03341050	MyoF (TCDM_08875)
	4,00E-143	DQ04_01331060	MyoD (TCDM_07686)
	2,00E-125	DQ04_00031100	Myo13 (TCDM_05821)
	8,00E-108	DQ04_00501100	Myo1 (TCDM_07314)
	5,00E-56	DQ04_03771000	MyoG (TCDM_02016)
Trypanosoma rangeli SC58	0.0	TRSC58_00243	MyoE (TCDM_06166)
	5,00E-128	TRSC58_06141	MyoD (TCDM_07686)
	6,00E-76	TRSC58_01895	MyoF (TCDM_08875)
	1,00E-25	TRSC58_00411	MyoB (TCDM_07433)
Trypanosoma vivax Y486	1,00E-119	TvY486_1117220	Myo13 (TCDM_05821)
	5,00E-113	TvY486_0403180	Myo1 (TCDM_07314)

**NCBI**			
**Genomes**	**BlastP e-value**	**ID of Hit**	**Reciprocal Blast**
**Green boxes are hits that correspond to MyoA genes**			
**Kinetoplastids**			
Trypanosoma theileri	0.0	ORC93297.1	MyoA (TCDM_09957)
	0.0	ORC88651.1	MyoB (TCDM_07433)
	0.0	ORC90398.1	MyoC (TCDM_02877)
	0.0	ORC86402.1	MyoE (TCDM_06166)
	2,00E-156	ORC89982.1	MyoF (TCDM_08875)
	7,00E-134	ORC86814.1	MyoD (TCDM_07686)
	3,00E-125	ORC87480.1	Myo13 (TCDM_05821)
	5,00E-110	ORC93509.1	Myo1 (TCDM_07314)
	7,00E-52	ORC91827.1	MyoG (TCDM_02016)
Angomonas deanei	3,00E-130	EPY29051.1	Myo13 (TCDM_05821)
	2,00E-129	EPY42972.1	Myo13 (TCDM_05821)
	1,00E-82	EPY31408.1	Myo1 (TCDM_07314)

	6,00E-27	EPY36601.1	Myo13 (TCDM_05821)
Strigomonas culicis	2,00E-119	EPY36094.1	Myo13 (TCDM_05821)
	3,00E-118	EPY22327.1	Myo13 (TCDM_05821)
Phytomonas sp. isolate EM1	9,00E-127	CCW60276.1	Myo13 (TCDM_05821)
Phytomonas sp. isolate Hart1	4,00E-127	CCW68456.1	Myo13 (TCDM_05821)
Bodo saltans	0.0	CUG89451.1	MyoA (TCDM_09957)
	0.0	CUF67578.1	MyoB (TCDM_07433)
	0.0	CUG93737.1	MyoB (TCDM_07433)
	0.0	CUG87167.1	MyoB (TCDM_07433)
	0.0	CUG90281.1	MyoB (TCDM_07433)
	0.0	CUF99905.1	MyoE (TCDM_06166)
	5,00E-169	CUG92185.1	MyoB (TCDM_07433)
	4,00E-162	CUG03397.1	MyoC (TCDM_02877)
	1,00E-161	CUG91579.1	MyoF (TCDM_08875)
	4,00E-124	CUG03103.1	Myo13 (TCDM_05821)
	9,00E-114	CUI15073.1	Myo1 (TCDM_07314)
	4,00E-100	CUF17710.1	MyoD (TCDM_07686)
	1,00E-51	CUG86977.1	MyoG (TCDM_02016)
**Examples of BlastP result against non-kinetoplastid species**			
Naegleria gruberi	8,00E-172	XP_002681567.1	MyoB (TCDM_07433)
Naegleria gruberi	1,00E-164	XP_002680898.1	MyoC (TCDM_02877)
Nelumbo nucifera	1,00E-158	XP_010263980.1	MyoA (TCDM_09957)
Jatropha curcas	1,00E-153	XP_020536711.1	MyoA (TCDM_09957)
Millerozyma farinosa	1,00E-153	CCE73260.1	MyoA (TCDM_09957)
Candida glabrata	2,00E-153	KTA96211.1	MyoA (TCDM_09957)
Arabidopsis thaliana	3,00E-152	NP_199203.1	MyoA (TCDM_09957)
Saccharomyces cerevisiae	1,00E-151	AJO95631.1	MyoA (TCDM_09957)
Scedosporium apiospermum	8,00E-151	XP_016638812.1	MyoA (TCDM_09957)
Asparagus officinalis	2,00E-150	XP_020271411.1	MyoA (TCDM_09957)

Table S10: Synteny conservation analysis of MyoA gene

Genome	Upstream gene	MyoA gene	Downstream gene 1	Downstream gene 2
**Green boxes highlight the species that have MyoA gene in conserved syntenic region in relation to *T. cruzi* MyoA**				
Blechomonas ayalai B08-376	Baya_313_0050	no orthologous found	no homologous found	Baya_313_0020
Crithidia fasciculata strain Cf-Cl	CFAC1_060005900	no remnants	CFAC1_060006200	CFAC1_060006300
Endotrypanum monterogeii strain LV88	no homologous found	no orthologous found	EMOLV88_340020200	EMOLV88_030005200
Leishmania aethiopica L147	LAEL147_000036000	no remnants	no homologous found	LAEL147_000036400
Leishmania arabica strain LEM1108	LARLEM1108_030005100	no remnants	no homologous found	LARLEM1108_030005500
Leishmania braziliensis MHOM/BR/75/M2903	LBRM2903_030005300	no remnants	no homologous found	LBRM2903_030005600
Leishmania braziliensis MHOM/BR/75/M2904	LbrM.03.0040	no remnants	no homologous found	LbrM.03.0070
Leishmania donovani BPK282A1	LdBPK_030020.1	no remnants	no homologous found	LdBPK_030050.1
Leishmania enriettii strain LEM3045	LENLEM3045_030005400	no remnants	no homologous found	LENLEM3045_030005600
Leishmania gerbilli strain LEM452	LGELEM452_030005100	no remnants	no homologous found	LGELEM452_030005500
Leishmania infantum JPCM5	LinJ.03.0020	no remnants	no homologous found	LinJ.03.0050
Leishmania major strain Friedlin	LmjF.03.0020	no remnants	no homologous found	LmjF.03.0060
Leishmania major strain LV39c5	LMJLV39_030005100	no remnants	no homologous found	LMJLV39_030005600
Leishmania major strain SD 75.1	LMJSD75_030005100	no remnants	no homologous found	LMJSD75_030005600
Leishmania mexicana MHOM/GT/2001/U1103	LmxM.03.0020	no remnants	no homologous found	LmxM.03.0060
Leishmania panamensis MHOM/COL/81/L13	LPAL13_030005100	no remnants	no homologous found	LPAL13_030005400
Leishmania tarentolae Parrot-TarII	LtaP03.0020	no remnants	no homologous found	LtaP03.0050
Leishmania tropica L590	LTRL590_030005100	no remnants	no homologous found	LTRL590_030005400
Leishmania turanica strain LEM423	LTULEM423_030005100	no remnants	no homologous found	LTULEM423_030005600
Leishmania sp. MAR LEM2494	LMARLEM2494_030005100	no remnants	no homologous found	LMARLEM2494_030005400
Leptomonas pyrrhocoris	LpyrH10_34_0030	no remnants	LpyrH10_34_0060	LpyrH10_34_0070
Leptomonas seymouri ATCC 30220	Lsey_0496_0010	no orthologous found	Lsey_0496_0040	Lsey_0188_0210
Trypanosoma brucei Lister strain 427	Tb427.10.3070	no remnants	no homologous found	Tb427.10.3080
Trypanosoma brucei brucei TREU927	Tb927.10.3070	no remnants	no homologous found	Tb927.10.3080
Trypanosoma brucei gambiense DAL972	Tbg972.10.3890	no remnants	no homologous found	Tbg972.10.3900
Trypanosoma congolense IL3000	TcIL3000_10_2520	no remnants	no homologous found	TcIL3000_10_2530
Trypanosoma cruzi CL Brener Esmeraldo-like	TcCLB.504867.129	TcCLB.504867.120	TcCLB.504867.114	TcCLB.504867.110
Trypanosoma cruzi CL Brener Non-Esmeraldo-like	TcCLB.510943.198	TcCLB.510943.190	TcCLB.510943.184	TcCLB.510943.180
Trypanosoma cruzi Dm28c	TCDM_09958	TCDM_09957	TCDM_09956	TCDM_09955
Trypanosoma cruzi marinkellei strain B7	Tc_MARK_3326	Tc_MARK_3325	Tc_MARK_3324	Tc_MARK_3323
Trypanosoma evansi strain STIB 805	TevSTIB805.10.3320	no remnants	no homologous found	TevSTIB805.10.3330
Trypanosoma grayi ANR4	DQ04_00821170	DQ04_00821150	DQ04_00821140	DQ04_00821130
*Trypanosoma rangeli SC58**	TRSC58_06135	no orthologous found	no homologous found	TRSC58_01067
Trypanosoma vivax Y486	TvY486_0043860	no orthologous found	no homologous found	TvY486_1003060
Trypanosoma theileri isolate Edinburgh TM35_Tth_5_V1[#]	NBCO01000001.1:195323-196111	NBCO01000001.1:199725-202568	NBCO01000001.1:203463-204452	na
Bodo saltans[#]	CYKH01001740.1:19137-19463	CYKH01001740.1 :26032-28656	CYKH01001740.1:29468-30439	na
Angomonas deanei strain ATCC PRA-265[#]	KV452474.1:6430-6768	no remnants	KV452474.1:10437-11390	na
Strigomonas culicis strain TCC012E[#]	AUXH01001153.1:1194-1640	no orthologous found	AUXH01000381.1:16237-17190	na
Phytomas sp EM1[#]	HF955080.1:150694-151404	no orthologous found	no homologous found	na
Phytomas sp Hart1[#]	HF955205.1:140282-141010	no orthologous found	no homologous found	na

The IDs correspond to the homologous of *T. cruzi* Dm28c upstream or downstream genes. In some cases, they are not the nearest gene in the analyzed species.
* Evaluation of synteny for this species was not possible since the genes were located in very small contigs.
\# For these species, the approximate chromosome position is presented. Genomes obtained from NCBI.
no orthologous found - we were not able to analyze the syntenic region and no orthologous gene was found throughout the genome
no remnants- we were able to analyze the syntenic region and no consistent signals of the presence of gene was found
na- not analyzed

Table S11: Summary of BlastP results for MyoB against sequences available at TritrypDB and NCBI

TritrypDB			
**Genomes**	**BlastP e-value**	**ID of Hit**	**Reciprocal Blast/Synteny**
**Green boxes are the hits that correspond to MyoB genes**			
Blechomonas ayalai B08-376	2,00E-118	Baya_099_0300	Myo13 (TCDM_05821)
	1,00E-94	Baya_227_0080	Myo1 (TCDM_07314)
Crithidia fasciculata strain Cf-Cl	4,00E-116	CFAC1_300048900	Myo13 (TCDM_05821)
	2,00E-39	CFAC1_290040800	Myo1 (TCDM_07314)
Endotrypanum monterogeii strain LV88	2,00E-120	EMOLV88_320042600	Myo13 (TCDM_05821)
	1,00E-40	EMOLV88_340015100	Myo1 (TCDM_07314)
Leishmania aethiopica L147	1,00E-120	LAEL147_000662900.1	Myo13 (TCDM_05821)
	6,00E-39	LAEL147_000713700	Myo1 (TCDM_07314)
Leishmania arabica strain LEM1108	1,00E-117	LARLEM1108_320047000	Myo13 (TCDM_05821)
	4,00E-39	LARLEM1108_000017500	Myo1 (TCDM_07314)
Leishmania braziliensis MHOM/BR/75/M2903	6,00E-98	LBRM2903_320050900	Myo13 (TCDM_05821)
	6,00E-43	LBRM2903_200016800	Myo1 (TCDM_07314)
Leishmania braziliensis MHOM/BR/75/M2904	1,00E-118	LbrM.32.4110	Myo13 (TCDM_05821)
	2,00E-42	LbrM.20.0970	Myo1 (TCDM_07314)
Leishmania donovani BPK282A1	1,00E-119	LdBPK_324020.1	Myo13 (TCDM_05821)
	3,00E-39	LdBPK_341070.1	Myo1 (TCDM_07314)
Leishmania enriettii strain LEM3045	7,00E-122	LENLEM3045_320047100	Myo13 (TCDM_05821)
	2,00E-39	LENLEM3045_340014600	Myo1 (TCDM_07314)
Leishmania gerbilli strain LEM452	3,00E-118	LGELEM452_320047000	Myo13 (TCDM_05821)
	3,00E-39	LGELEM452_000011900	Myo1 (TCDM_07314)
Leishmania infantum JPCM5	2,00E-119	LinJ.32.4020	Myo13 (TCDM_05821)
	4,00E-39	LinJ.34.1070	Myo1 (TCDM_07314)
Leishmania major strain Friedlin	2,00E-120	LmjF.32.3870	Myo13 (TCDM_05821)
	4,00E-38	LmjF.34.1000	Myo1 (TCDM_07314)
Leishmania major strain LV39c5	4,00E-120	LMJLV39_320046600	Myo13 (TCDM_05821)
	3,00E-38	LMJLV39_340016900	Myo1 (TCDM_07314)
Leishmania major strain SD 75.1	1,00E-119	LMJSD75_320046800	Myo13 (TCDM_05821)
	1,00E-38	LMJSD75_340016900	Myo1 (TCDM_07314)
Leishmania mexicana MHOM/GT/2001/U1103	1,00E-117	LmxM.31.3870	Myo13 (TCDM_05821)
	2,00E-38	LmxM.33.1000	Myo1 (TCDM_07314)
Leishmania panamensis MHOM/COL/81/L13	9,00E-119	LPAL13_320047300	Myo13 (TCDM_05821)
	4,00E-42	LPAL13_200014600	Myo1 (TCDM_07314)
Leishmania tarentolae Parrot-TarII	2,00E-126	LtaP32.4040	Myo13 (TCDM_05821)
	5,00E-40	LtaP34.1150	Myo1 (TCDM_07314)
Leishmania tropica L590	2,00E-118	LTRL590_320046900	Myo13 (TCDM_05821)
	9,00E-39	LTRL590_340015400	Myo1 (TCDM_07314)
Leishmania turanica strain LEM423	2,00E-117	LTULEM423_320047500	Myo13 (TCDM_05821)
	5,00E-39	LTULEM423_000006800	Myo1 (TCDM_07314)
Leishmania sp. MAR LEM2494	1,00E-116	LMARLEM2494_320047400	Myo13 (TCDM_05821)

	5,00E-39	LMARLEM2494_340015700	Myo1 (TCDM_07314)
Leptomonas pyrrhocoris	2,00E-116	LpyrH10_02_4330	Myo13 (TCDM_05821)
	3,00E-38	LpyrH10_05_1440	Myo1 (TCDM_07314)
Leptomonas seymouri ATCC 30220	4,00E-116	Lsey_0246_0030	Myo13 (TCDM_05821)
	5,00E-38	Lsey_0435_0030	Myo1 (TCDM_07314)
Trypanosoma brucei Lister strain 427	8,00E-116	Tb427tmp.01.7990	Myo13 (TCDM_05821)
	7,00E-94	Tb427.04.3380	Myo1 (TCDM_07314)
Trypanosoma brucei brucei TREU927	6,00E-116	Tb927.11.16310	Myo13 (TCDM_05821)
	9,00E-94	Tb927.4.3380	Myo1 (TCDM_07314)
Trypanosoma brucei gambiense DAL972	1,00E-115	Tbg972.11.18310	Myo13 (TCDM_05821)
	9,00E-94	Tbg972.4.3390	Myo1 (TCDM_07314)
Trypanosoma congolense IL3000	6,00E-94	TcIL3000_4_3080	Myo1 (TCDM_07314)
Trypanosoma cruzi marinkellei strain B7	0.0	Tc_MARK_594	MyoB (TCDM_07433)
	0.0	Tc_MARK_3325	MyoA (TCDM_09957)
	0.0	Tc_MARK_3528	MyoC (TCDM_02877)
	0.0	Tc_MARK_8887	MyoE (TCDM_06166)
	6,00E-143	Tc_MARK_4644	MyoF (TCDM_08875)
	1,00E-126	Tc_MARK_845	MyoD (TCDM_07686)
	2,00E-116	Tc_MARK_4039	Myo13 (TCDM_05821)
	5,00E-57	Tc_MARK_4094	MyoG (TCDM_02016)
	1,00E-32	Tc_MARK_2656	Myo1 (TCDM_07314)
Trypanosoma evansi strain STIB 805	3,00E-116	TevSTIB805.11_01.16880	Myo13 (TCDM_05821)
	3,00E-94	TevSTIB805.4.3470	Myo1 (TCDM_07314)
Trypanosoma grayi ANR4	0.0	DQ04_01671000	MyoB (TCDM_07433)
	0.0	DQ04_02991050	MyoC (TCDM_02877)
	0.0	DQ04_00821150	MyoA (TCDM_09957)
	0.0	DQ04_01311010	MyoE (TCDM_06166)
	1,00E-172	DQ04_03341050	MyoF (TCDM_08875)
	9,00E-121	DQ04_01331060	MyoD (TCDM_07686)
	7,00E-115	DQ04_00031100	Myo13 (TCDM_05821)
	5,00E-96	DQ04_00501100	Myo1 (TCDM_07314)
	2,00E-53	DQ04_03771000	MyoG (TCDM_02016)
Trypanosoma rangeli SC58	0.0	TRSC58_00243	MyoE (TCDM_06166)
	2,00E-167	TRSC58_00411	MyoB (TCDM_07433)
	4,00E-121	TRSC58_06141	MyoD (TCDM_07686)
	3,00E-73	TRSC58_01895	MyoF (TCDM_08875)
Trypanosoma vivax Y486	7,00E-122	TvY486_1117220	Myo13 (TCDM_05821)
	3,00E-98	TvY486_0403180	Myo1 (TCDM_07314)
NCBI			
Genomes	**BlastP e-value**	**ID of Hit**	**Reciprocal Blast**
Green boxes are hits that correspond to MyoB genes			
Kinetoplastids			
Trypanosoma theileri	0.0	ORC88651.1	MyoB (TCDM_07433)
	0.0	ORC93297.1	MyoA (TCDM_09957)
	0.0	ORC90398.1	MyoC (TCDM_02877)
	0.0	ORC86402.1	MyoE (TCDM_06166)
	2,00E-171	ORC89982.1	MyoF (TCDM_08875)
	6,00E-122	ORC86814.1	MyoD (TCDM_07686)
	2,00E-120	ORC87480.1	Myo13 (TCDM_05821)
	4,00E-95	ORC93509.1	Myo1 (TCDM_07314)
	1,00E-47	ORC91827.1	MyoG (TCDM_02016)
Angomonas deanei	5,00E-112	EPY29051.1	Myo13 (TCDM_05821)

	8,00E-108	EPY42972.1	Myo13 (TCDM_05821)
	1,00E-72	EPY31408.1	Myo1 (TCDM_07314)
	2,00E-26	EPY36601.1	Myo13 (TCDM_05821)
Strigomonas culicis	5,00E-116	EPY36094.1	Myo13 (TCDM_05821)
	2,00E-114	EPY22327.1	Myo13 (TCDM_05821)
	1,00E-28	EPY17541.1	Myo1 (TCDM_07314)
	2,00E-27	EPY17540.1	Myo1 (TCDM_07314)
Phytomonas sp. isolate EM1	1,00E-113	CCW60276.1	Myo13 (TCDM_05821)
Phytomonas sp. isolate Hart1	3,00E-118	CCW68456.1	Myo13 (TCDM_05821)
Bodo saltans	0.0	CUF67578.1	MyoB (TCDM_07433)
	0.0	CUG90281.1	MyoB (TCDM_07433)
	0.0	CUG92185.1	MyoB (TCDM_07433)
	0.0	CUG87167.1	MyoB (TCDM_07433)
	0.0	CUG93737.1	MyoB (TCDM_07433)
	0.0	CUG89451.1	MyoA (TCDM_09957)
	0.0	CUF99905.1	MyoE (TCDM_06166)
	1,00E-177	CUG03397.1	MyoC (TCDM_02877)
	2,00E-169	CUG91579.1	MyoF (TCDM_08875)
	3,00E-130	CUG03103.1	Myo13 (TCDM_05821)
	6,00E-108	CUI15073.1	Myo1 (TCDM_07314)
	3,00E-88	CUF17710.1	MyoD (TCDM_07686)
	2,00E-62	CUG86977.1	MyoG (TCDM_02016)
**Examples of BlastP result against non-kinetoplastid species**			
Naegleria gruberi	0.0	XP_002681567.1	MyoB (TCDM_07433)
Naegleria gruberi	4,00E-157	XP_002680898.1	MyoC (TCDM_02877)
Acanthisitta chloris	3,00E-135	XP_009079550.1	MyoA (TCDM_09957)
Nicotiana tabacum	2,00E-134	XP_016453995.1	MyoA (TCDM_09957)
Lupinus angustifolius	9,00E-134	XP_019460646.1	MyoA (TCDM_09957)
Ceratitis capitata	2,00E-133	JAB98664.1	MyoA (TCDM_09957)
Glycine max	3,00E-133	XP_020713148.1	Myo1 (TCDM_07314)
Nelumbo nucifera	7,00E-133	XP_010263980.1	MyoA (TCDM_09957)
Eutrema salsugineum	2,00E-131	XP_006403166.1	MyoA (TCDM_09957)
Pundamilia nyererei	2,00E-131	XP_005723787.1	MyoB (TCDM_07433)

Table S12: Synteny conservation analysis of MyoB gene

Genome	Upstream gene from *T. cruzi marinkellei*	Upstream gene	MyoB gene	Downstream gene
Green boxes highlight the species that have MyoB gene in conserved syntenic region in relation to *T. cruzi* MyoB				
Blechomonas ayalai B08-376	Non-syntenic		no orthologous found	Baya_011_0540
Crithidia fasciculata strain Cf-Cl	Non-syntenic		no orthologous found	CFAC1_300008200
Endotrypanum monterogeii strain LV88	Non-syntenic		no orthologous found	EMOLV88_000042800
Leishmania aethiopica L147	Non-syntenic		no orthologous found	LAEL147_000483900
Leishmania arabica strain LEM1108	Non-syntenic		no orthologous found	LARLEM1108_280007800
Leishmania braziliensis MHOM/BR/75/M2903	Non-syntenic		no orthologous found	LBRM2903_280008000
Leishmania braziliensis MHOM/BR/75/M2904	Non-syntenic		no orthologous found	LbrM.28.0250
Leishmania donovani BPK282A1	Non-syntenic		no orthologous found	LdBPK_280240.1
Leishmania enriettii strain LEM3045	Non-syntenic		no orthologous found	LENLEM3045_280007600
Leishmania gerbilli strain LEM452	Non-syntenic		no orthologous found	LGELEM452_280007900
Leishmania infantum JPCM5	Non-syntenic		no orthologous found	LinJ.28.0240
Leishmania major strain Friedlin	Non-syntenic		no orthologous found	LmjF.28.0240
Leishmania major strain LV39c5	Non-syntenic		no orthologous found	LMJLV39_280007600
Leishmania major strain SD 75.1	Non-syntenic		no orthologous found	LMJSD75_280007500
Leishmania mexicana MHOM/GT/2001/U1103	Non-syntenic		no orthologous found	LmxM.28.0240
Leishmania panamensis MHOM/COL/81/L13	Non-syntenic		no orthologous found	LPAL13_280007700
Leishmania tarentolae Parrot-TarII	Non-syntenic		no orthologous found	LtaP28.0250
Leishmania tropica L590	Non-syntenic		no orthologous found	LTRL590_280007400
Leishmania turanica strain LEM423	Non-syntenic		no orthologous found	LTULEM423_280007700
Leishmania sp. MAR LEM2494	Non-syntenic		no orthologous found	LMARLEM2494_280008300
Leptomonas pyrrhocoris	Non-syntenic		no orthologous found	LpyrH10_02_7920
Leptomonas seymouri ATCC 30220	Non-syntenic		no orthologous found	Lsey_0055_0250
Trypanosoma brucei Lister strain 427	Tb427tmp.02.5270		no remnants	Tb427tmp.02.5280
Trypanosoma brucei brucei TREU927	Tb927.11.7370		no remnants	Tb927.11.7380
Trypanosoma brucei gambiense DAL972	Tbg972.11.8420		no remnants	Tbg972.11.8440
Trypanosoma congolense IL3000	TcIL3000.11.7950		no remnants	TcIL3000.11.7960
Trypanosoma cruzi CL Brener Esmeraldo-like	TcCLB.506779.170	No VIPER	TcCLB.506779.190	TcCLB.506779.200
Trypanosoma cruzi CL Brener Non-Esmeraldo-like	TcCLB.511153.50	No VIPER	TcCLB.511151.100	TcCLB.511151.90
Trypanosoma cruzi Dm28c	TCDM_08611	TCDM_07431 (VIPER)**	TCDM_07433	TCDM_07435
Trypanosoma cruzi marinkellei strain B7	Tc_MARK_591	missing data	Tc_MARK_594	Tc_MARK_595
Trypanosoma evansi strain STIB 805	TevSTIB805.11_01.7670		no remnants	TevSTIB805.11_01.7680
Trypanosoma grayi ANR4	DQ04_09301010		DQ04_01671000	DQ04_01671010
Trypanosoma rangeli SC58*	TRSC58_00142*		TRSC58_00411*	TRSC58_03201*
Trypanosoma vivax Y486	TvY486_1107910		no remnants	TvY486_1107920
Trypanosoma theileri isolate Edinburgh TM35_Tth_5_V1#	na		NBCO01000016.1:480713-483802	NBCO01000016.1:485117-486970
Bodo saltans#	na		CYKH01000380.1:2298-5309	CYKH01000380.1:6507 -8396
Angomonas deanei strain ATCC PRA-265#	KV452521.1:296221-297261		no orthologous found	KV452521.1:55105-56922
Strigomonas culicis strain TCC012E#	AUXH01000214.1:14337-15359		no orthologous found	AUXH01000035.1:30296-32119
Phytomas sp EM1#	HF955068.1:301422-302504		no orthologous found	HF955068.1: 520780-522594
Phytomas sp Hart1#	HF955202.1:1276759 -1277829		no orthologous found	HF955202.1:1494195 -1496150

The IDs correspond to the homologous of *T. cruzi* Dm28c or *T. cruzi marinkellei* upstream or downstream genes. In some cases, they are not the nearest gene in the analyzed species.
* Evaluation of synteny for this species was not possible since the genes were located in very small contigs.
# For these species, the approximate chromosome position is presented. Genomes obtained from NCBI.
no orthologous found - we were not able to analyze the syntenic region and no orthologous gene was found throughout the genome.
no remnants- we were able to analyze the syntenic region and no consistent signals of the presence of gene was found.
na- not analyzed.
missing region - genomic region that is filled with N.
There is an insertion of VIPER retrotransposon upstream of *T. cruzi* Dm28c MyoB gene. This insertion is not found in the other *T. cruzi* strains.
** end of contig.

Table S13: Summary of BlastP results for MyoC against sequences available at TritrypDB and NCBI

TritrypDB			
**Genomes**	**BlastP e-value**	**ID of Hit**	**Reciprocal Blast/Synteny**
**Green boxes are the hits that correspond to MyoC genes**			
Blechomonas ayalai B08-376	4,00E-118	Baya_099_0300	Myo13 (TCDM_05821)
	1,00E-86	Baya_227_0080	Myo1 (TCDM_07314)
Crithidia fasciculata strain Cf-Cl	8,00E-125	CFAC1_300048900	Myo13 (TCDM_05821)
	5,00E-36	CFAC1_290040800	Myo1 (TCDM_07314)
Endotrypanum monterogeii strain LV88	2,00E-121	EMOLV88_320042600	Myo13 (TCDM_05821)
	1,00E-37	EMOLV88_340015100	Myo1 (TCDM_07314)
Leishmania aethiopica L147	4,00E-123	LAEL147_000662900.1	Myo13 (TCDM_05821)
	2,00E-37	LAEL147_000713700	Myo1 (TCDM_07314)
Leishmania arabica strain LEM1108	5,00E-125	LARLEM1108_320047000	Myo13 (TCDM_05821)
	2,00E-36	LARLEM1108_000017500	Myo1 (TCDM_07314)
Leishmania braziliensis MHOM/BR/75/M2903	9,00E-100	LBRM2903_320050900	Myo13 (TCDM_05821)
	1,00E-38	LBRM2903_200016800	Myo1 (TCDM_07314)
Leishmania braziliensis MHOM/BR/75/M2904	3,00E-125	LbrM.32.4110	Myo13 (TCDM_05821)
	1,00E-37	LbrM.20.0970	Myo1 (TCDM_07314)
Leishmania donovani BPK282A1	2,00E-125	LdBPK_324020.1	Myo13 (TCDM_05821)
	1,00E-36	LdBPK_341070.1	Myo1 (TCDM_07314)
Leishmania enriettii strain LEM3045	2,00E-126	LENLEM3045_320047100	Myo13 (TCDM_05821)
	2,00E-37	LENLEM3045_340014600	Myo1 (TCDM_07314)
Leishmania gerbilli strain LEM452	1,00E-124	LGELEM452_320047000	Myo13 (TCDM_05821)
	6,00E-37	LGELEM452_000011900	Myo1 (TCDM_07314)
Leishmania infantum JPCM5	4,00E-125	LinJ.32.4020	Myo13 (TCDM_05821)
	2,00E-36	LinJ.34.1070	Myo1 (TCDM_07314)
Leishmania major strain Friedlin	5,00E-125	LmjF.32.3870	Myo13 (TCDM_05821)
	9,00E-37	LmjF.34.1000	Myo1 (TCDM_07314)
Leishmania major strain LV39c5	3,00E-124	LMJLV39_320046600	Myo13 (TCDM_05821)
	1,00E-36	LMJLV39_340016900	Myo1 (TCDM_07314)
Leishmania major strain SD 75.1	7,00E-125	LMJSD75_320046800	Myo13 (TCDM_05821)
	9,00E-37	LMJSD75_340016900	Myo1 (TCDM_07314)
Leishmania mexicana MHOM/GT/2001/U1103	7,00E-126	LmxM.31.3870	Myo13 (TCDM_05821)
	2,00E-37	LmxM.33.1000	Myo1 (TCDM_07314)
Leishmania panamensis MHOM/COL/81/L13	3,00E-125	LPAL13_320047300	Myo13 (TCDM_05821)
	2,00E-38	LPAL13_200014600	Myo1 (TCDM_07314)
Leishmania tarentolae Parrot-TarII	2,00E-126	LtaP32.4040	Myo13 (TCDM_05821)
	5,00E-38	LtaP34.1150	Myo1 (TCDM_07314)
Leishmania tropica L590	9,00E-125	LTRL590_320046900	Myo13 (TCDM_05821)
	3,00E-37	LTRL590_340015400	Myo1 (TCDM_07314)
Leishmania turanica strain LEM423	2,00E-124	LTULEM423_320047500	Myo13 (TCDM_05821)
	6,00E-37	LTULEM423_000006800	Myo1 (TCDM_07314)
Leishmania sp. MAR LEM2494	2,00E-121	LMARLEM2494_320047400	Myo13 (TCDM_05821)
	3,00E-37	LMARLEM2494_340015700	Myo1 (TCDM_07314)

Leptomonas pyrrhocoris	7,00E-125	LpyrH10_02_4330	Myo13 (TCDM_05821)
	1,00E-36	LpyrH10_05_1440	Myo1 (TCDM_07314)
Leptomonas seymouri ATCC 30220	5,00E-119	Lsey_0246_0030	Myo13 (TCDM_05821)
	1,00E-36	Lsey_0435_0030	Myo1 (TCDM_07314)
Trypanosoma brucei Lister strain 427	3,00E-108	Tb427tmp.01.7990	Myo13 (TCDM_05821)
	8,00E-88	Tb427.04.3380	Myo1 (TCDM_07314)
Trypanosoma brucei brucei TREU927	1,00E-109	Tb927.11.16310	Myo13 (TCDM_05821)
	1,00E-87	Tb927.4.3380	Myo1 (TCDM_07314)
Trypanosoma brucei gambiense DAL972	1,00E-107	Tbg972.11.18310	Myo13 (TCDM_05821)
	1,00E-87	Tbg972.4.3390	Myo1 (TCDM_07314)
Trypanosoma congolense IL3000	2,00E-87	TcIL3000_4_3080	Myo1 (TCDM_07314)
Trypanosoma cruzi marinkellei strain B7	0.0	Tc_MARK_3528	MyoC (TCDM_02877)
	0.0	Tc_MARK_3325	MyoA (TCDM_09957)
	0.0	Tc_MARK_594	MyoB (TCDM_07433)
	0.0	Tc_MARK_8887	MyoE (TCDM_06166)
	2,00E-149	Tc_MARK_4644	MyoF (TCDM_08875)
	9,00E-141	Tc_MARK_845	MyoD (TCDM_07686)
	1,00E-116	Tc_MARK_4039	Myo13 (TCDM_05821)
	6,00E-56	Tc_MARK_4094	MyoG (TCDM_02016)
	1,00E-34	Tc_MARK_2656	Myo1 (TCDM_07314)
Trypanosoma evansi strain STIB 805	2,00E-108	TevSTIB805.11_01.16880	Myo13 (TCDM_05821)
	3,00E-88	TevSTIB805.4.3470	Myo1 (TCDM_07314)
Trypanosoma grayi ANR4	0.0	DQ04_02991050	MyoC (TCDM_02877)
	0.0	DQ04_01671000	MyoB (TCDM_07433)
	0.0	DQ04_00821150	MyoA (TCDM_09957)
	0.0	DQ04_03341050	MyoF (TCDM_08875)
	0.0	DQ04_01311010	MyoE (TCDM_06166)
	5,00E-131	DQ04_01331060	MyoD (TCDM_07686)
	6,00E-114	DQ04_00031100	Myo13 (TCDM_05821)
	1,00E-92	DQ04_00501100	Myo1 (TCDM_07314)
	2,00E-53	DQ04_03771000	MyoG (TCDM_02016)
Trypanosoma rangeli SC58	0.0	TRSC58_00243	MyoE (TCDM_06166)
	2,00E-137	TRSC58_06141	MyoD (TCDM_07686)
	4,00E-82	TRSC58_01895	MyoF (TCDM_08875)
	6,00E-30	TRSC58_00411	MyoB (TCDM_07433)
Trypanosoma vivax Y486	5,00E-118	TvY486_1117220	Myo13 (TCDM_05821)
	2,00E-95	TvY486_0403180	Myo1 (TCDM_07314)

NCBI			
**Genomes**	**BlastP e-value**	**ID of Hit**	**Reciprocal Blast**
**Green boxes are hits that correspond to MyoC genes**			
**Kinetoplastids**			
Trypanosoma theileri	0.0	ORC90398.1	MyoC (TCDM_02877)
	0.0	ORC89982.1	MyoF (TCDM_08875)
	0.0	ORC88651.1	MyoB (TCDM_07433)
	0.0	ORC86402.1	MyoE (TCDM_06166)
	0.0	ORC93297.1	MyoA (TCDM_09957)
	5,00E-130	ORC86814.1	MyoD (TCDM_07686)
	8,00E-127	ORC87480.1	Myo13 (TCDM_05821)
	4,00E-88	ORC93509.1	Myo1 (TCDM_07314)
	4,00E-52	ORC91827.1	MyoG (TCDM_02016)
Angomonas deanei	4,00E-118	EPY29051.1	Myo13 (TCDM_05821)
	4,00E-115	EPY42972.1	Myo13 (TCDM_05821)
	2,00E-72	EPY31408.1	Myo1 (TCDM_07314)

	1,00E-25	EPY36601.1	Myo13 (TCDM_05821)
Strigomonas culicis	1,00E-117	EPY36094.1	Myo13 (TCDM_05821)
	1,00E-115	EPY22327.1	Myo13 (TCDM_05821)
	1,00E-30	EPY17540.1	Myo1 (TCDM_07314)
	5,00E-27	EPY17541.1	Myo1 (TCDM_07314)
Phytomonas sp. isolate EM1	9,00E-114	CCW60276.1	Myo13 (TCDM_05821)
Phytomonas sp. isolate Hart1	7,00E-116	CCW68456.1	Myo13 (TCDM_05821)
Bodo saltans	0.0	CUG03397.1	MyoC (TCDM_02877)
	0.0	CUG93737.1	MyoB (TCDM_07433)
	0.0	CUG87167.1	MyoB (TCDM_07433)
	0.0	CUG89451.1	MyoA (TCDM_09957)
	0.0	CUF67578.1	MyoB (TCDM_07433)
	0.0	CUG90281.1	MyoB (TCDM_07433)
	0.0	CUF99905.1	MyoE (TCDM_06166)
	7,00E-173	CUG91579.1	MyoF (TCDM_08875)
	2,00E-165	CUG92185.1	MyoB (TCDM_07433)
	7,00E-123	CUG03103.1	Myo13 (TCDM_05821)
	9,00E-106	CUI15073.1	Myo1 (TCDM_07314)
	3,00E-96	CUF17710.1	MyoD (TCDM_07686)
	4,00E-55	CUG86977.1	MyoG (TCDM_02016)
**Examples of BlastP result against non-kinetoplastid species**			
Naegleria gruberi	1,00E-172	XP_002681567.1	MyoB (TCDM_07433)
Naegleria gruberi	4,00E-167	XP_002680898.1	MyoC (TCDM_02877)
Brassica napus	3,00E-151	XP_013658193.1	MyoA (TCDM_09957)
Raphanus sativus	1,00E-147	XP_018483436.1	MyoC (TCDM_02877)
Nelumbo nucifera	2,00E-144	XP_010263980.1	MyoA (TCDM_09957)
Arabidopsis thaliana	4,00E-144	NP_001190465.1	MyoA (TCDM_09957)
Setaria italica	2,00E-143	NP_191375.1	MyoC (TCDM_02877)
Prunus mume	2,00E-143	XP_008231403.1	MyoC (TCDM_02877)
Macleaya cordata	8,00E-143	OVA16401.1	MyoC (TCDM_02877)
Zea mays	1,00E-141	AQK62955.1	MyoA (TCDM_09957)

Table S14: Synteny conservation analysis of MyoC gene

Genome	Upstream gene 2	Upstream gene	MyoC gene	Downstream gene
**Green boxes highlight the species that have MyoC gene in conserved syntenic region in relation to *T. cruzi* MyoC**				
Blechomonas ayalai B08-376	Baya_196_0100	no homologous found	no remnants	Baya_196_0110
Crithidia fasciculata strain Cf-Cl	CFAC1_280051500	no homologous found	no remnants	CFAC1_280051600
Endotrypanum monterogeii strain LV88	EMOLV88_360077100	no homologous found	no remnants	EMOLV88_360077200
Leishmania aethiopica L147	LAEL147_000859700	no homologous found	no remnants	LAEL147_000859800
Leishmania arabica strain LEM1108	LARLEM1108_360056300	no homologous found	no remnants	LARLEM1108_360056400
Leishmania braziliensis MHOM/BR/75/M2903	LBRM2903_350059300	no homologous found	no remnants	LBRM2903_350059500
Leishmania braziliensis MHOM/BR/75/M2904	LbrM.35.4860	no homologous found	no remnants	LbrM.35.4870
Leishmania donovani BPK282A1	LdBPK_364840.1	no homologous found	no remnants	LdBPK_364850.1
Leishmania enriettii strain LEM3045	LENLEM3045_360056400	no homologous found	no remnants	LENLEM3045_360056500
Leishmania gerbilli strain LEM452	LGELEM452_360056300	no homologous found	no remnants	LGELEM452_360056400
Leishmania infantum JPCM5	LinJ.36.4840	no homologous found	no remnants	LinJ.36.4850
Leishmania major strain Friedlin	LmjF.36.4610	no homologous found	no remnants	LmjF.36.4620
Leishmania major strain LV39c5	LMJLV39_360057600	no homologous found	no remnants	LMJLV39_360057700
Leishmania major strain SD 75.1	LMJSD75_360057400	no homologous found	no remnants	LMJSD75_360057500
Leishmania mexicana MHOM/GT/2001/U1103	LmxM.36.4610	no homologous found	no remnants	LmxM.36.4620
Leishmania panamensis MHOM/COL/81/L13	LPAL13_350055500	no homologous found	no remnants	LPAL13_350055600
Leishmania tarentolae Parrot-TarII	LtaP36.4730	no homologous found	no remnants	LtaP36.4740
Leishmania tropica L590	LTRL590_360057400	no homologous found	no remnants	LTRL590_360057500
Leishmania turanica strain LEM423	LTULEM423_360056700	no homologous found	no remnants	LTULEM423_360056800
Leishmania sp. MAR LEM2494	LMARLEM2494_360055900	no homologous found	no remnants	LMARLEM2494_360056000
Leptomonas pyrrhocoris	LpyrH10_06_2390	no homologous found	no remnants	LpyrH10_06_2400
Leptomonas seymouri ATCC 30220	Lsey_0034_0270	no homologous found	no remnants	Lsey_0034_0260
Trypanosoma brucei Lister strain 427	Tb427.10.9950	Tb427.10.9960	no remnants	Tb427.10.9970
Trypanosoma brucei brucei TREU927	Tb927.10.9950	Tb927.10.9960	no remnants	Tb927.10.9970
Trypanosoma brucei gambiense DAL972	Tbg972.10.12150	Tbg972.10.12160	no remnants	Tbg972.10.12170
Trypanosoma congolense IL3000	TcIL3000_10_8410	TcIL3000_10_8400	no remnants	TcIL3000_10_8390
Trypanosoma cruzi CL Brener Esmeraldo-like	TcCLB.504103.50	TcCLB.504103.40	TcCLB.504103.30	TcCLB.504103.20
Trypanosoma cruzi CL Brener Non-Esmeraldo-like	TcCLB.503847.40	TcCLB.503847.30	TcCLB.503847.20	TcCLB.503847.10
Trypanosoma cruzi Dm28c	TCDM_02879	TCDM_02878	TCDM_02877	TCDM_02875
Trypanosoma cruzi marinkellei strain B7	Tc_MARK_3526	Tc_MARK_3527	Tc_MARK_3528	Tc_MARK_3529
Trypanosoma evansi strain STIB 805	TevSTIB805.10.10540	TevSTIB805.10.10550	no remnants	TevSTIB805.10.10560
Trypanosoma grayi ANR4	DQ04_02991030	DQ04_02991040	DQ04_02991050	DQ04_02991060
Trypanosoma rangeli SC58*	TRSC58_00445*	no homologous found	no orthologous found	TRSC58_05565*
Trypanosoma vivax Y486	TvY486_1009830	TvY486_1009840	no remnants	TvY486_1009850
Trypanosoma theileri isolate Edinburgh TM35_Tth_5_V1#	NBCO01000008.1:246852-247925	na	NBCO01000008.1:250212-252797	NBCO01000008.1:254595-257489
Bodo saltans#	CYKH01001951.1:26743-27465	na	CYKH01000509.1:3-2114**	CYKH01000509.1:4824-6419
Angomonas deanei strain ATCC PRA-265#	KV452479.1:139849-140907	na	no remnants	KV452479.1 :136406-138376
Strigomonas culicis strain TCC012E#	AUXH01000513.1:7084-8286	na	no remnants	AUXH01000513.1:11943-12656
Phytomas sp EM1#	no homologous found	no homologous found	no orthologous found	HF955078.1: 140293-141828
Phytomas sp Hart1#	no homologous found	no homologous found	no orthologous found	HF955205.1:486725-487900

The IDs correspond to the homologous of *T. cruzi* Dm28c upstream or downstream genes. In some cases, they are not the nearest gene in the analyzed species.
* Evaluation of synteny for this species was not possible since the genes were located in very small contigs.
# For these species, the approximate chromosome position is presented. Genomes obtained from NCBI.
no orthologous found - we were not able to analyze the syntenic region and no orthologous gene was found throughout the genome.
no remnants- we were able to analyze the syntenic region and no consistent signals of the presence of gene was found.
na- not analyzed.
** end of contig.

Table S15: Summary of BlastP results for MyoD against sequences available at TritrypDB and NCBI

TritrypDB			
**Genomes**	**BlastP e-value**	**ID of Hit**	**Reciprocal Blast/Synteny**
**Green boxes are the hits that correspond to MyoD genes**			
Blechomonas ayalai B08-376	1,00E-101	Baya_099_0300	Myo13 (TCDM_05821)
	6,00E-83	Baya_227_0080	Myo1 (TCDM_07314)
Crithidia fasciculata strain Cf-Cl	3,00E-92	CFAC1_300048900	Myo13 (TCDM_05821)
	5,00E-36	CFAC1_290040800	Myo1 (TCDM_07314)
Endotrypanum monterogeii strain LV88	2,00E-102	EMOLV88_320042600	Myo13 (TCDM_05821)
	1,00E-38	EMOLV88_340015100	Myo1 (TCDM_07314)
Leishmania aethiopica L147	2,00E-95	LAEL147_000662900.1	Myo13 (TCDM_05821)
	1,00E-36	LAEL147_000713700	Myo1 (TCDM_07314)
Leishmania arabica strain LEM1108	5,00E-94	LARLEM1108_320047000	Myo13 (TCDM_05821)
	3,00E-37	LARLEM1108_000017500	Myo1 (TCDM_07314)
Leishmania braziliensis MHOM/BR/75/M2903	3,00E-67	LBRM2903_320050900	Myo13 (TCDM_05821)
	5,00E-39	LBRM2903_200016800	Myo1 (TCDM_07314)
Leishmania braziliensis MHOM/BR/75/M2904	6,00E-96	LbrM.32.4110	Myo13 (TCDM_05821)
	1,00E-38	LbrM.20.0970	Myo1 (TCDM_07314)
Leishmania donovani BPK282A1	3,00E-95	LdBPK_324020.1	Myo13 (TCDM_05821)
	6,00E-37	LdBPK_341070.1	Myo1 (TCDM_07314)
Leishmania enriettii strain LEM3045	2,00E-99	LENLEM3045_320047100	Myo13 (TCDM_05821)
	8,00E-37	LENLEM3045_340014600	Myo1 (TCDM_07314)
Leishmania gerbilli strain LEM452	9,00E-95	LGELEM452_320047000	Myo13 (TCDM_05821)
	8,00E-37	LGELEM452_000011900	Myo1 (TCDM_07314)
Leishmania infantum JPCM5	8,00E-96	LinJ.32.4020	Myo13 (TCDM_05821)
	1,00E-36	LinJ.34.1070	Myo1 (TCDM_07314)
Leishmania major strain Friedlin	4,00E-94	LmjF.32.3870	Myo13 (TCDM_05821)
	2,00E-36	LmjF.34.1000	Myo1 (TCDM_07314)
Leishmania major strain LV39c5	3,00E-94	LMJLV39_320046600	Myo13 (TCDM_05821)
	2,00E-36	LMJLV39_340016900	Myo1 (TCDM_07314)
Leishmania major strain SD 75.1	1,00E-93	MJSD75_320046800	Myo13 (TCDM_05821)
	2,00E-36	LMJSD75_340016900	Myo1 (TCDM_07314)
Leishmania mexicana MHOM/GT/2001/U1103	4,00E-94	LmxM.31.3870	Myo13 (TCDM_05821)
	1,00E-40	LmxM.33.1000	Myo1 (TCDM_07314)
Leishmania panamensis MHOM/COL/81/L13	1,00E-95	LPAL13_320047300	Myo13 (TCDM_05821)
	6,00E-39	LPAL13_200014600	Myo1 (TCDM_07314)
Leishmania tarentolae Parrot-TarII	1,00E-94	LtaP32.4040	Myo13 (TCDM_05821)
	3,00E-35	LtaP34.1150	Myo1 (TCDM_07314)
Leishmania tropica L590	1,00E-93	LTRL590_320046900	Myo13 (TCDM_05821)
	1,00E-36	LTRL590_340015400	Myo1 (TCDM_07314)
Leishmania turanica strain LEM423	4,00E-95	LTULEM423_320047500	Myo13 (TCDM_05821)
	3,00E-37	LTULEM423_000006800	Myo1 (TCDM_07314)
Leishmania sp. MAR LEM2494	6,00E-95	LMARLEM2494_320047400	Myo13 (TCDM_05821)
	4,00E-37	LMARLEM2494_340015700	Myo1 (TCDM_07314)

Leptomonas pyrrhocoris	4,00E-95	LpyrH10_02_4330	Myo13 (TCDM_05821)
	2,00E-35	LpyrH10_05_1440	Myo1 (TCDM_07314)
Leptomonas seymouri ATCC 30220	3,00E-93	Lsey_0435_0030	Myo1 (TCDM_07314)
	2,00E-35	Lsey_0246_0030	Myo13 (TCDM_05821)
Trypanosoma brucei Lister strain 427	4,00E-96	Tb427tmp.01.7990	Myo13 (TCDM_05821)
	5,00E-84	Tb427.04.3380	Myo1 (TCDM_07314)
Trypanosoma brucei brucei TREU927	7,00E-96	Tb927.11.16310	Myo13 (TCDM_05821)
	1,00E-83	Tb927.4.3380	Myo1 (TCDM_07314)
Trypanosoma brucei gambiense DAL972	5,00E-95	Tbg972.11.18310	Myo13 (TCDM_05821)
	6,00E-84	Tbg972.4.3390	Myo1 (TCDM_07314)
Trypanosoma congolense IL3000	5,00E-83	TcIL3000_4_3080	Myo1 (TCDM_07314)
Trypanosoma cruzi marinkellei strain B7	0.0	Tc_MARK_845	MyoD (TCDM_07686)
	3,00E-130	Tc_MARK_3325	MyoA (TCDM_09957)
	1,00E-128	Tc_MARK_3528	MyoC (TCDM_02877)
	2,00E-114	Tc_MARK_594	MyoB (TCDM_07433)
	3,00E-113	Tc_MARK_8887	MyoE (TCDM_06166)
	1,00E-103	Tc_MARK_4644	MyoF (TCDM_08875)
	2,00E-92	Tc_MARK_4039	Myo13 (TCDM_05821)
	1,00E-39	Tc_MARK_4094	MyoG (TCDM_02016)
	6,00E-31	Tc_MARK_2656	Myo1 (TCDM_07314)
Trypanosoma evansi strain STIB 805	1,00E-94	TevSTIB805.11_01.16880	Myo13 (TCDM_05821)
	4,00E-84	TevSTIB805.4.3470	Myo1 (TCDM_07314)
Trypanosoma grayi ANR4	0.0	DQ04_01331060	MyoD (TCDM_07686)
	5,00E-137	DQ04_00821150	MyoA (TCDM_09957)
	4,00E-128	DQ04_02991050	MyoC (TCDM_02877)
	5,00E-123	DQ04_01671000	MyoB (TCDM_07433)
	4,00E-114	DQ04_00031100	Myo13 (TCDM_05821)
	2,00E-109	DQ04_03341050	MyoF (TCDM_08875)
	7,00E-95	DQ04_01311010	MyoE (TCDM_06166)
	9,00E-88	DQ04_00501100	Myo1 (TCDM_07314)
	2,00E-43	DQ04_03771000	MyoG (TCDM_02016)
Trypanosoma rangeli SC58	0.0	TRSC58_06141	MyoD (TCDM_07686)
	1,00E-112	TRSC58_00243	MyoE (TCDM_06166)
	5,00E-65	TRSC58_01895	MyoF (TCDM_08875)
	8,00E-12	TRSC58_00411	MyoB (TCDM_07433)
Trypanosoma vivax Y486	2,00E-100	TvY486_1117220	Myo13 (TCDM_05821)
	1,00E-85	TvY486_0403180	Myo1 (TCDM_07314)
**NCBI**			
**Genomes**	**BlastP e-value**	**ID of Hit**	**Reciprocal Blast**
**Green boxes are hits that correspond to MyoD genes**			
**Kinetoplastids**			
Trypanosoma theileri	0.0	ORC86814.1	MyoD (TCDM_07686)
	8,00E-135	ORC93297.1	MyoA (TCDM_09957)
	7,00E-126	ORC90398.1	MyoC (TCDM_02877)
	3,00E-124	ORC88651.1	MyoB (TCDM_07433)
	2,00E-120	ORC86402.1	MyoE (TCDM_06166)
	5,00E-114	ORC89982.1	MyoF (TCDM_08875)
	2,00E-96	ORC87480.1	Myo13 (TCDM_05821)
	1,00E-76	ORC93509.1	Myo1 (TCDM_07314)
	7,00E-44	ORC91827.1	MyoG (TCDM_02016)
Angomonas deanei	3,00E-97	EPY29051.1	Myo13 (TCDM_05821)
	3,00E-97	EPY42972.1	Myo13 (TCDM_05821)
	2,00E-56	EPY31408.1	Myo1 (TCDM_07314)

	7,00E-10	EPY24057.1	MyoG (TCDM_02016)
Strigomonas culicis	5,00E-96	EPY36094.1	Myo13 (TCDM_05821)
	6,00E-94	EPY22327.1	Myo13 (TCDM_05821)
	2,00E-24	EPY17541.1	Myo1 (TCDM_07314)
	5,00E-24	EPY17540.1	Myo1 (TCDM_07314)
Phytomonas sp. isolate EM1	7,00E-102	CCW60276.1	Myo13 (TCDM_05821)
Phytomonas sp. isolate Hart1	6,00E-91	CCW68456.1	Myo13 (TCDM_05821)
Bodo saltans	0.0	CUF17710.1	MyoD (TCDM_07686)
	2,00E-139	CUG89451.1	MyoA (TCDM_09957)
	2,00E-124	CUG93737.1	MyoB (TCDM_07433)
	5,00E-124	CUF99905.1	MyoE (TCDM_06166)
	1,00E-119	CUG90281.1	MyoB (TCDM_07433)
	2,00E-117	CUF67578.1	MyoB (TCDM_07433)
	5,00E-117	CUG91579.1	MyoF (TCDM_08875)
	4,00E-111	CUG87167.1	MyoB (TCDM_07433)
	1,00E-102	CUG03103.1	Myo13 (TCDM_05821)
	3,00E-102	CUG03397.1	MyoC (TCDM_02877)
	2,00E-99	CUG92185.1	MyoB (TCDM_07433)
	4,00E-93	CUI15073.1	Myo1 (TCDM_07314)
	2,00E-45	CUG86977.1	MyoG (TCDM_02016)
**Examples of BlastP result against non-kinetoplastid species**			
Naegleria gruberi	6,00E-135	XP_002680898.1	MyoC (TCDM_02877)
Naegleria gruberi	4,00E-128	XP_002681567.1	MyoB (TCDM_07433)
Glycine max	6,00E-118	XP_003539582.1	Myo1 (TCDM_07314)
Arachis ipaensis	1,00E-116	XP_016171514.1	Myo1 (TCDM_07314)
Solanum tuberosum	4,00E-115	XP_015158716.1	Myo1 (TCDM_07314)
Ipomoea nil	3,00E-113	XP_019196973.1	Myo1 (TCDM_07314)
Lupinus angustifolius	3,00E-112	XP_019443991.1	Myo1 (TCDM_07314)
Noccaea caerulescens	1,00E-111	JAU90744.1	Myo1 (TCDM_07314)
Cajanus cajan	1,00E-110	XP_020223619.1	Myo1 (TCDM_07314)
Brassica napus	8,00E-110	CDY52239.1	Myo1 (TCDM_07314)

Table S16: Synteny conservation analysis of MyoD gene

Genome	Upstream gene 2	Upstream gene 1	MyoD	Downstream gene
**Green boxes highlight the species that have MyoD gene in syntenic region in relation to *T. cruzi* MyoD**				
Blechomonas ayalai B08-376	Baya_242_0030	Baya_242_0060	no orthologous found	Baya_030_0470
Crithidia fasciculata strain Cf-Cl	CFAC1_040005700	CFAC1_040006500	no remnants	CFAC1_040006800
Endotrypanum monterogeii strain LV88	no homologous found	no homologous found	no orthologous found	EMOLV88_000045500
Leishmania aethiopica L147	LAEL147_000131100	no homologous found	no remnants	LAEL147_000131600
Leishmania arabica strain LEM1108	LARLEM1108_100005500	no homologous found	no remnants	LARLEM1108_100006000
Leishmania braziliensis MHOM/BR/75/M2903	LBRM2903_100005500	no homologous found	no remnants	LBRM2903_100006000
Leishmania braziliensis MHOM/BR/75/M2904	LbrM.10.0060	no homologous found	no remnants	LbrM.10.0110
Leishmania donovani BPK282A1	LdBPK_100040.1	no homologous found	no remnants	LdBPK_100090.1
Leishmania enriettii strain LEM3045	LENLEM3045_100005500	no homologous found	no remnants	LENLEM3045_100006100
Leishmania gerbilli strain LEM452	LGELEM452_100005600	no homologous found	no remnants	LGELEM452_100006200
Leishmania infantum JPCM5	LinJ.10.0040	no homologous found	no remnants	LinJ.10.0090
Leishmania major strain Friedlin	LmjF.10.0060	no homologous found	no remnants	LmjF.10.0110
Leishmania major strain LV39c5	LMJLV39_100005400	no homologous found	no remnants	LMJLV39_100005900
Leishmania major strain SD 75.1	LMJSD75_100005500	no homologous found	no remnants	LMJSD75_100006000
Leishmania mexicana MHOM/GT/2001/U1103	LmxM.10.0060	no homologous found	no remnants	LmxM.10.0110
Leishmania panamensis MHOM/COL/81/L13	LPAL13_100005500	no homologous found	no remnants	LPAL13_100006000
Leishmania tarentolae Parrot-TarII	LtaP10.0030	no homologous found	no remnants	LtaP10.0080
Leishmania tropica L590	LTRL590_000021100	no homologous found	no remnants	LTRL590_000021500
Leishmania turanica strain LEM423	LTULEM423_100005500	no homologous found	no remnants	LTULEM423_100006000
Leishmania sp. MAR LEM2494	LMARLEM2494_10000550 0	no homologous found	no remnants	LMARLEM2494_10000610 0
Leptomonas pyrrhocoris	LpyrH10_22_0090	LpyrH10_22_0210	no remnants	LpyrH10_22_0240
Leptomonas seymouri ATCC 30220*	Lsey_0407_0030	end of contig*	no orthologous found	Lsey_0061_0260
Trypanosoma brucei Lister strain 427	Tb427.08.3910	Tb427.08.3880	no remnants	Tb427.08.3860
Trypanosoma brucei brucei TREU927	Tb927.8.3910	Tb927.8.3880	no remnants	Tb927.8.3860
Trypanosoma brucei gambiense DAL972	Tbg972.8.3620	Tbg972.8.3600	no remnants	Tbg972.8.3580
Trypanosoma congolense IL3000	TcIL3000_8_3770	TcIL3000_8_3760	no remnants	TcIL3000_8_3740
Trypanosoma cruzi CL Brener Non-Esmeraldo-like	TcCLB.509663.30	TcCLB.509663.20	TcCLB.509663.10	TcCLB.507705.20
Trypanosoma cruzi Dm28c	TCDM_07688	TCDM_07687	TCDM_07686	TCDM_07685
Trypanosoma cruzi marinkellei strain B7	Tc_MARK_844	no homologous found	Tc_MARK_845	Tc_MARK_846
Trypanosoma evansi strain STIB 805	TevSTIB805.8.4020	TevSTIB805.8.4000	no remnants	TevSTIB805.8.3970
Trypanosoma grayi ANR4	DQ04_01331020	DQ04_01331050	DQ04_01331060	DQ04_01331100
Trypanosoma rangeli SC58*	TRSC58_05665*	TRSC58_04640*	TRSC58_06141*	TRSC58_01661*
Trypanosoma vivax Y486*	TvY486_0003360	TvY486_0803310*	no orthologous found	TvY486_0030270*
Trypanosoma theileri isolate Edinburgh TM35_Tth_5_V1[#]	NBCO01000025.1:17726-18763	NBCO01000025.1:22145-22714	NBCO01000025.1:23001-26609	NBCO01000025.1:28998-30218
Bodo saltans[#]	CYKH01001743.1:35550-34504	CYKH01002148.1:66566-67090	CYKH01000256.1:1535-3661	CYKH01001579.1:10640-11677
Angomonas deanei strain ATCC PRA-265[#]	na	KV452474.1:239577-240119	no remnants	KV452474.1:236688-237647
Strigomonas culicis strain TCC012E[#]	na	AUXH01001331.1:1798-2370	no orthologous found	AUXH01000944.1:1182-2174
Phytomas sp EM1[#]	HF955102.1:114485-115501	no homologous found	no remnants	HF955102.1:107736-108782
Phytomas sp Hart1[#]	HF955210.1:507858-508874	no homologous found	no remnants	HF955210.1:500894-501901

The IDs correspond to the homologous of *T. cruzi* Dm28c upstream or downstream genes. In some cases, they are not the nearest gene in the analyzed species.
* Evaluation of synteny for this species was not possible since the genes were located in very small contigs.
\# For these species, the approximate chromosome position is presented. Genomes obtained from NCBI.
no orthologous found - we were not able to analyze the syntenic region and no orthologous gene was found throughout the genome.
no remnants- we were able to analyze the syntenic region and no consistent signals of the presence of gene was found.
na- not analyzed.

Table S17: Summary of BlastP results for MyoE against sequences available at TritrypDB and NCBI

TritrypDB			
**Genomes**	**BlastP e-value**	**ID of Hit**	**Reciprocal Blast/Synteny**
**Green boxes are the hits that correspond to MyoE genes**			
Blechomonas ayalai B08-376	2,00E-110	Baya_099_0300	Myo13 (TCDM_05821)
	3,00E-87	Baya_227_0080	Myo1 (TCDM_07314)
Crithidia fasciculata strain Cf-Cl	1,00E-117	CFAC1_300048900	Myo13 (TCDM_05821)
	1,00E-38	CFAC1_290040800	Myo1 (TCDM_07314)
Endotrypanum monterogeii strain LV88	1,00E-105	EMOLV88_320042600	Myo13 (TCDM_05821)
	7,00E-41	EMOLV88_340015100	Myo1 (TCDM_07314)
Leishmania aethiopica L147	1,00E-112	LAEL147_000662900.1	Myo13 (TCDM_05821)
	1,00E-41	LAEL147_000713700	Myo1 (TCDM_07314)
Leishmania arabica strain LEM1108	5,00E-112	LARLEM1108_320047000	Myo13 (TCDM_05821)
	1,00E-40	LARLEM1108_000017500	Myo1 (TCDM_07314)
Leishmania braziliensis MHOM/BR/75/M2903	4,00E-94	LBRM2903_320050900	Myo13 (TCDM_05821)
	3,00E-43	LBRM2903_200016800	Myo1 (TCDM_07314)
Leishmania braziliensis MHOM/BR/75/M2904	4,00E-113	LbrM.32.4110	Myo13 (TCDM_05821)
	2,00E-42	LbrM.20.0970	Myo1 (TCDM_07314)
Leishmania donovani BPK282A1	2,00E-112	LdBPK_324020.1	Myo13 (TCDM_05821)
	1,00E-39	LdBPK_341070.1	Myo1 (TCDM_07314)
Leishmania enriettii strain LEM3045	3,00E-115	LENLEM3045_320047100	Myo13 (TCDM_05821)
	1,00E-39	LENLEM3045_340014600	Myo1 (TCDM_07314)
Leishmania gerbilli strain LEM452	2,00E-111	LGELEM452_320047000	Myo13 (TCDM_05821)
	1,00E-40	LGELEM452_000011900	Myo1 (TCDM_07314)
Leishmania infantum JPCM5	6,00E-113	LinJ.32.4020	Myo13 (TCDM_05821)
	1,00E-39	LinJ.34.1070	Myo1 (TCDM_07314)
Leishmania major strain Friedlin	5,00E-113	LmjF.32.3870	Myo13 (TCDM_05821)
	2,00E-40	LmjF.34.1000	Myo1 (TCDM_07314)
Leishmania major strain LV39c5	4,00E-113	LMJLV39_320046600	Myo13 (TCDM_05821)
	1,00E-40	LMJLV39_340016900	Myo1 (TCDM_07314)
Leishmania major strain SD 75.1	4,00E-113	LMJSD75_320046800	Myo13 (TCDM_05821)
	5,00E-41	LMJSD75_340016900	Myo1 (TCDM_07314)
Leishmania mexicana MHOM/GT/2001/U1103	3,00E-112	LmxM.31.3870	Myo13 (TCDM_05821)
	6,00E-41	LmxM.33.1000	Myo1 (TCDM_07314)
Leishmania panamensis MHOM/COL/81/L13	4,00E-113	LPAL13_320047300	Myo13 (TCDM_05821)
	5,00E-42	LPAL13_200014600	Myo1 (TCDM_07314)
Leishmania tarentolae Parrot-TarII	3,00E-118	LtaP32.4040	Myo13 (TCDM_05821)
	1,00E-41	LtaP34.1150	Myo1 (TCDM_07314)
Leishmania tropica L590	5,00E-113	LTRL590_320046900	Myo13 (TCDM_05821)
	5,00E-41	LTRL590_340015400	Myo1 (TCDM_07314)
Leishmania turanica strain LEM423	9,00E-113	LTULEM423_320047500	Myo13 (TCDM_05821)
	2,00E-40	LTULEM423_000006800	Myo1 (TCDM_07314)
Leishmania sp. MAR LEM2494	2,00E-110	LMARLEM2494_320047400	Myo13 (TCDM_05821)
	1,00E-78	LMARLEM2494_340015700	Myo1 (TCDM_07314)

Leptomonas pyrrhocoris	9,00E-115	LpyrH10_02_4330	Myo13 (TCDM_05821)
	2,00E-73	LpyrH10_05_1440	Myo1 (TCDM_07314)
Leptomonas seymouri ATCC 30220	1,00E-114	Lsey_0246_0030	Myo13 (TCDM_05821)
	2,00E-39	Lsey_0435_0030	Myo1 (TCDM_07314)
Trypanosoma brucei Lister strain 427	3,00E-105	Tb427tmp.01.7990	Myo13 (TCDM_05821)
	6,00E-89	Tb427.04.3380	Myo1 (TCDM_07314)
Trypanosoma brucei brucei TREU927	1,00E-106	Tb927.11.16310	Myo13 (TCDM_05821)
	6,00E-89	Tb927.4.3380	Myo1 (TCDM_07314)
Trypanosoma brucei gambiense DAL972	1,00E-104	Tbg972.11.18310	Myo13 (TCDM_05821)
	1,00E-88	Tbg972.4.3390	Myo1 (TCDM_07314)
Trypanosoma congolense IL3000	5,00E-87	TcIL3000_4_3080	Myo1 (TCDM_07314)
Trypanosoma cruzi marinkellei strain B7	0.0	Tc_MARK_8887	MyoE (TCDM_06166)
	0.0	Tc_MARK_3528	MyoC (TCDM_02877)
	0.0	Tc_MARK_594	MyoB (TCDM_07433)
	6,00E-179	Tc_MARK_3325	MyoA (TCDM_09957)
	2,00E-138	Tc_MARK_4644	MyoF (TCDM_08875)
	9,00E-112	Tc_MARK_845	MyoD (TCDM_07686)
	8,00E-107	Tc_MARK_4039	Myo13 (TCDM_05821)
	6,00E-42	Tc_MARK_4094	MyoG (TCDM_02016)
	7,00E-32	Tc_MARK_2656	Myo1 (TCDM_07314)
Trypanosoma evansi strain STIB 805	3,00E-106	TevSTIB805.11_01.16880	Myo13 (TCDM_05821)
	5,00E-89	TevSTIB805.4.3470	Myo1 (TCDM_07314)
Trypanosoma grayi ANR4	0.0	DQ04_01311010	MyoE (TCDM_06166)
	0.0	DQ04_02991050	MyoC (TCDM_02877)
	0.0	DQ04_01671000	MyoB (TCDM_07433)
	0.0	DQ04_00821150	MyoA (TCDM_09957)
	7,00E-151	DQ04_03341050	MyoF (TCDM_08875)
	6,00E-108	DQ04_00031100	Myo13 (TCDM_05821)
	6,00E-107	DQ04_01331060	MyoD (TCDM_07686)
	7,00E-84	DQ04_00501100	Myo1 (TCDM_07314)
	1,00E-40	DQ04_03771000	MyoG (TCDM_02016)
Trypanosoma rangeli SC58	0.0	TRSC58_00243	MyoE (TCDM_06166)
	1,00E-111	TRSC58_06141	MyoD (TCDM_07686)
	9,00E-68	TRSC58_01895	MyoF (TCDM_08875)
	1,00E-32	TRSC58_00411	MyoB (TCDM_07433)
Trypanosoma vivax Y486	5,00E-110	TvY486_1117220	Myo13 (TCDM_05821)
	3,00E-88	TvY486_0403180	Myo1 (TCDM_07314)

**NCBI**			
**Genomes**	**BlastP e-value**	**ID of Hit**	**Reciprocal Blast**
Green boxes are hits that correspond to MyoE genes			
**Kinetoplastids**			
Trypanosoma theileri	0.0	ORC86402.1	MyoE (TCDM_06166)
	0.0	ORC93297.1	MyoA (TCDM_09957)
	0.0	ORC88651.1	MyoB (TCDM_07433)
	0.0	ORC90398.1	MyoC (TCDM_02877)
	2,00E-150	ORC89982.1	MyoF (TCDM_08875)
	1,00E-114	ORC87480.1	Myo13 (TCDM_05821)
	2,00E-109	ORC86814.1	MyoD (TCDM_07686)
	2,00E-87	ORC93509.1	Myo1 (TCDM_07314)
	7,00E-51	ORC91827.1	MyoG (TCDM_02016)
Angomonas deanei	2,00E-111	EPY29051.1	Myo13 (TCDM_05821)
	2,00E-105	EPY42972.1	Myo13 (TCDM_05821)
	5,00E-69	EPY31408.1	Myo1 (TCDM_07314)

	5,00E-31	EPY36601.1	Myo13 (TCDM_05821)
Strigomonas culicis	2,00E-106	EPY36094.1	Myo13 (TCDM_05821)
	3,00E-105	EPY22327.1	Myo13 (TCDM_05821)
	1,00E-29	EPY17541.1	Myo1 (TCDM_07314)
	2,00E-23	EPY17540.1	Myo1 (TCDM_07314)
Phytomonas sp. isolate EM1	1,00E-108	CCW60276.1	Myo13 (TCDM_05821)
Phytomonas sp. isolate Hart1	2,00E-107	CCW68456.1	Myo13 (TCDM_05821)
Bodo saltans	0.0	CUF99905.1	MyoE (TCDM_06166)
	0.0	CUF67578.1	MyoB (TCDM_07433)
	0.0	CUG93737.1	MyoB (TCDM_07433)
	0.0	CUG87167.1	MyoB (TCDM_07433)
	0.0	CUG90281.1	MyoB (TCDM_07433)
	0.0	CUG89451.1	MyoA (TCDM_09957)
	0.0	CUG92185.1	MyoB (TCDM_07433)
	2,00E-156	CUG03397.1	MyoC (TCDM_02877)
	1,00E-144	CUG91579.1	MyoF (TCDM_08875)
	5,00E-114	CUG03103.1	Myo13 (TCDM_05821)
	3,00E-84	CUI15073.1	Myo1 (TCDM_07314)
	3,00E-82	CUF17710.1	MyoD (TCDM_07686)
	8,00E-46	CUG86977.1	MyoG (TCDM_02016)
**Examples of BlastP result against non-kinetoplastid species**			
Naegleria gruberi	5,00E-155	XP_002680898.1	MyoC (TCDM_02877)
Naegleria gruberi	9,00E-146	XP_002681567.1	MyoB (TCDM_07433)
Acanthamoeba castellanii	4,00E-131	XP_004341424.1	Myo1 (TCDM_07314)
Glycine max	4,00E-122	KRH40874.1	MyoA (TCDM_09957)
Lupinus angustifolius	4,00E-122	XP_019462253.1	MyoA (TCDM_09957)
Camelus ferus	7,00E-121	XP_014408951.1	MyoA (TCDM_09957)
Camelina sativa	5,00E-120	XP_010438394.1	MyoC (TCDM_02877)
Ursus maritimus	6,00E-120	XP_008684247.1	Myo1 (TCDM_07314)
Gossypium raimondii	9,00E-120	XP_012486844.1	MyoA (TCDM_09957)
Capsella rubella	1,00E-118	XP_006285522.1	MyoA (TCDM_09957)

Table S18: Synteny conservation analysis of MyoE gene

Genome	Upstream gene 2	Upstream gene	MyoE gene	Downstream gene
**Green boxes highlight the species that have MyoE gene in syntenic conserved region in relation to *T. cruzi* MyoE**				
Blechomonas ayalai B08-376	Non-syntenic	Non-syntenic	no orthologous found	Baya_051_0390
Crithidia fasciculata strain Cf-Cl	Non-syntenic	Non-syntenic	no orthologous found	CFAC1_230052500
Endotrypanum monterogeii strain LV88	Non-syntenic	Non-syntenic	no orthologous found	EMOLV88_270005200
Leishmania aethiopica L147	Non-syntenic	Non-syntenic	no orthologous found	LAEL147_000454600
Leishmania arabica strain LEM1108	Non-syntenic	Non-syntenic	no orthologous found	LARLEM1108_270005300
Leishmania braziliensis MHOM/BR/75/M2903	Non-syntenic	Non-syntenic	no orthologous found	LBRM2903_270005500
Leishmania braziliensis MHOM/BR/75/M2904	Non-syntenic	Non-syntenic	no orthologous found	LbrM.27.0040
Leishmania donovani BPK282A1	Non-syntenic	Non-syntenic	no orthologous found	LdBPK_270030.1
Leishmania enriettii strain LEM3045	Non-syntenic	Non-syntenic	no orthologous found	LENLEM3045_270005800
Leishmania gerbilli strain LEM452	Non-syntenic	Non-syntenic	no orthologous found	LGELEM452_270005500
Leishmania infantum JPCM5	Non-syntenic	Non-syntenic	no orthologous found	LinJ.27.0030
Leishmania major strain Friedlin	Non-syntenic	Non-syntenic	no orthologous found	LmjF.27.0030
Leishmania major strain LV39c5	Non-syntenic	Non-syntenic	no orthologous found	LMJLV39_270005400
Leishmania major strain SD 75.1	Non-syntenic	Non-syntenic	no orthologous found	LMJSD75_270005300
Leishmania mexicana MHOM/GT/2001/U1103	Non-syntenic	Non-syntenic	no orthologous found	LmxM.27.0030
Leishmania panamensis MHOM/COL/81/L13	Non-syntenic	Non-syntenic	no orthologous found	LPAL13_270005600
Leishmania tarentolae Parrot-TarII	Non-syntenic	Non-syntenic	no orthologous found	LtaP27.0030
Leishmania tropica L590	Non-syntenic	Non-syntenic	no orthologous found	LTRL590_270005400
Leishmania turanica strain LEM423	Non-syntenic	Non-syntenic	no orthologous found	LTULEM423_270005300
Leishmania sp. MAR LEM2494	Non-syntenic	Non-syntenic	no orthologous found	LMARLEM2494_270005200
Leptomonas pyrrhocoris	Non-syntenic	Non-syntenic	no orthologous found	LpyrH10_35_0690
Leptomonas seymouri ATCC 30220	Non-syntenic	Non-syntenic	no orthologous found	Lsey_0203_0030
Trypanosoma brucei Lister strain 427	Non-syntenic	Tb427.05.3510	no orthologous found	Tb427.05.3520
Trypanosoma brucei brucei TREU927	Non-syntenic	Tb927.5.3510	no orthologous found	Tb927.5.3520
Trypanosoma brucei gambiense DAL972	Non-syntenic	Tbg972.5.4900	no orthologous found	Tbg972.5.4910
Trypanosoma congolense IL3000	Non-syntenic	TcIL3000_0_18230	no remnants	TcIL3000_0_18240
Trypanosoma cruzi CL Brener Esmeraldo-like	TcCLB.503531.30	TcCLB.503531.49	TcCLB.507811.120	TcCLB.507811.114
Trypanosoma cruzi CL Brener Non-Esmeraldo-like	TcCLB.511649.60	TcCLB.511649.70	TcCLB.511649.80	TcCLB.511649.90
Trypanosoma cruzi Dm28c	TCDM_06169	TCDM_06168	TCDM_06166	TCDM_06165
Trypanosoma cruzi marinkellei strain B7	Non-syntenic	Tc_MARK_8886	Tc_MARK_8887	Tc_MARK_8888
Trypanosoma evansi strain STIB 805	Non-syntenic	TevSTIB805.5.4010	no remnants	TevSTIB805.5.4030
Trypanosoma grayi ANR4	Non-syntenic	DQ04_01311020	DQ04_01311010	DQ04_01311000
Trypanosoma rangeli SC58*	TRSC58_03613*	TRSC58_04549*	TRSC58_00243*	TRSC58_06252*
Trypanosoma vivax Y486	Non-syntenic	TvY486_0502915	no remnants	TvY486_0502920
Trypanosoma theileri isolate Edinburgh TM35_Tth_5_V1#	na	NBCO01000028.1:40649-43624	NBCO01000028.1:44879-48238	NBCO01000028.1:48706-49713
Bodo saltans#	na	CYKH01000484.1:-42822 45299	CYKH01000484.1:38814-40898	CYKH01000484.1:36491-37480
Angomonas deanei strain ATCC PRA-265#	na	KV452486.1:448448-451423	no orthologous found	KV452486.1:271754-272674
Strigomonas culicis strain TCC012E#	na	AUXH01000015.1:57420-60395	no remnants	AUXH01000015.1:55134-56096
Phytomas sp EM1#	na	HF955062.1:54216-57182	no remnants	HF955062.1:58164-59147
Phytomas sp Hart1#	na	HF955202.1:926251-929217	no remnants	HF955202.1:924341-925324

The IDs correspond to the homologous of *T. cruzi* Dm28c upstream or downstream genes. In some cases, they are not the nearest gene in the analyzed species.
* Evaluation of synteny for this species was not possible since the genes were located in very small contigs.
# For these species, the approximate chromosome position is presented. Genomes obtained from NCBI.
no orthologous found - we were not able to analyze the syntenic region and no orthologous gene was found throughout the genome.
no remnants- we were able to analyze the syntenic region and no consistent signals of the presence of gene was found.
na- not analyzed.

Table S19: Summary of BlastP results for MyoF against sequences available at TritrypDB and NCBI

TritrypDB			
Genomes	BlastP e-value	ID of Hit	Reciprocal Blast/Synteny
Green boxes are the hits that correspond to MyoF genes			
Blechomonas ayalai B08-376	1,00E-102	Baya_099_0300	Myo13 (TCDM_05821)
	2,00E-83	Baya_227_0080	Myo1 (TCDM_07314)
Crithidia fasciculata strain Cf-Cl	2,00E-107	CFAC1_300048900	Myo13 (TCDM_05821)
	6,00E-42	CFAC1_290040800	Myo1 (TCDM_07314)
Endotrypanum monterogeii strain LV88	2,00E-100	EMOLV88_320042600	Myo13 (TCDM_05821)
	2,00E-39	EMOLV88_340015100	Myo1 (TCDM_07314)
Leishmania aethiopica L147	1,00E-110	LAEL147_000662900.1	Myo13 (TCDM_05821)
	3,00E-40	LAEL147_000713700	Myo1 (TCDM_07314)
Leishmania arabica strain LEM1108	3,00E-110	LARLEM1108_320047000	Myo13 (TCDM_05821)
	6,00E-40	LARLEM1108_000017500	Myo1 (TCDM_07314)
Leishmania braziliensis MHOM/BR/75/M2903	6,00E-88	LBRM2903_320050900	Myo13 (TCDM_05821)
	3,00E-43	LBRM2903_200016800	Myo1 (TCDM_07314)
Leishmania braziliensis MHOM/BR/75/M2904	7,00E-111	LbrM.32.4110	Myo13 (TCDM_05821)
	1,00E-42	LbrM.20.0970	Myo1 (TCDM_07314)
Leishmania donovani BPK282A1	3,00E-111	LdBPK_324020.1	Myo13 (TCDM_05821)
	6,00E-39	LdBPK_341070.1	Myo1 (TCDM_07314)
Leishmania enriettii strain LEM3045	7,00E-111	LENLEM3045_320047100	Myo13 (TCDM_05821)
	2,00E-40	LENLEM3045_340014600	Myo1 (TCDM_07314)
Leishmania gerbilli strain LEM452	2,00E-109	LGELEM452_320047000	Myo13 (TCDM_05821)
	1,00E-38	LGELEM452_000011900	Myo1 (TCDM_07314)
Leishmania infantum JPCM5	2,00E-111	LinJ.32.4020	Myo13 (TCDM_05821)
	6,00E-39	LinJ.34.1070	Myo1 (TCDM_07314)
Leishmania major strain Friedlin	2,00E-111	LmjF.32.3870	Myo13 (TCDM_05821)
	2,00E-39	LmjF.34.1000	Myo1 (TCDM_07314)
Leishmania major strain LV39c5	3,00E-111	LMJLV39_320046600	Myo13 (TCDM_05821)
	2,00E-39	LMJLV39_340016900	Myo1 (TCDM_07314)
Leishmania major strain SD 75.1	2,00E-111	LMJSD75_320046800	Myo13 (TCDM_05821)
	9,00E-40	LMJSD75_340016900	Myo1 (TCDM_07314)
Leishmania mexicana MHOM/GT/2001/U1103	6,00E-108	LmxM.31.3870	Myo13 (TCDM_05821)
	3,00E-41	LmxM.33.1000	Myo1 (TCDM_07314)
Leishmania panamensis MHOM/COL/81/L13	3,00E-111	LPAL13_320047300	Myo13 (TCDM_05821)
	1,00E-42	LPAL13_200014600	Myo1 (TCDM_07314)
Leishmania tarentolae Parrot-TarII	5,00E-108	LtaP32.4040	Myo13 (TCDM_05821)
	3,00E-41	LtaP34.1150	Myo1 (TCDM_07314)
Leishmania tropica L590	2,00E-110	LTRL590_320046900	Myo13 (TCDM_05821)
	1,00E-40	LTRL590_340015400	Myo1 (TCDM_07314)
Leishmania turanica strain LEM423	3,00E-111	LTULEM423_320047500	Myo13 (TCDM_05821)
	7,00E-40	LTULEM423_000006800	Myo1 (TCDM_07314)
Leishmania sp. MAR LEM2494	2,00E-109	LMARLEM2494_320047400	Myo13 (TCDM_05821)
	2,00E-42	LMARLEM2494_340015700	Myo1 (TCDM_07314)

Leptomonas pyrrhocoris	2,00E-109	LpyrH10_02_4330	Myo13 (TCDM_05821)
	1,00E-72	LpyrH10_05_1440	Myo1 (TCDM_07314)
Leptomonas seymouri ATCC 30220	2,00E-106	Lsey_0246_0030	Myo13 (TCDM_05821)
	4,00E-72	Lsey_0435_0030	Myo1 (TCDM_07314)
Trypanosoma brucei Lister strain 427	2,00E-106	Tb427tmp.01.7990	Myo13 (TCDM_05821)
	9,00E-85	Tb427.04.3380	Myo1 (TCDM_07314)
Trypanosoma brucei brucei TREU927	2,00E-107	Tb927.11.16310	Myo13 (TCDM_05821)
	6,00E-85	Tb927.4.3380	Myo1 (TCDM_07314)
Trypanosoma brucei gambiense DAL972	5,00E-105	Tbg972.11.18310	Myo13 (TCDM_05821)
	7,00E-85	Tbg972.4.3390	Myo1 (TCDM_07314)
Trypanosoma congolense IL3000	5,00E-86	TcIL3000_4_3080	Myo1 (TCDM_07314)
Trypanosoma cruzi marinkellei strain B7	0.0	Tc_MARK_4644	MyoF (TCDM_08875)
	3,00E-175	Tc_MARK_3528	MyoC (TCDM_02877)
	1,00E-165	Tc_MARK_594	MyoB (TCDM_07433)
	3,00E-163	Tc_MARK_8887	MyoE (TCDM_06166)
	6,00E-162	Tc_MARK_3325	MyoA (TCDM_09957)
	4,00E-118	Tc_MARK_845	MyoD (TCDM_07686)
	6,00E-103	Tc_MARK_4039	Myo13 (TCDM_05821)
	9,00E-53	Tc_MARK_4094	MyoG (TCDM_02016)
	4,00E-30	Tc_MARK_2656	Myo1 (TCDM_07314)
Trypanosoma evansi strain STIB 805	1,00E-104	TevSTIB805.11_01.16880	Myo13 (TCDM_05821)
	2,00E-85	TevSTIB805.4.3470	Myo1 (TCDM_07314)
Trypanosoma grayi ANR4	0.0	DQ04_03341050	MyoF (TCDM_08875)
	7,00E-176	DQ04_02991050	MyoC (TCDM_02877)
	2,00E-170	DQ04_01671000	MyoB (TCDM_07433)
	1,00E-169	DQ04_01311010	MyoE (TCDM_06166)
	1,00E-166	DQ04_00821150	MyoA (TCDM_09957)
	3,00E-118	DQ04_01331060	MyoD (TCDM_07686)
	1,00E-101	DQ04_00031100	Myo13 (TCDM_05821)
	2,00E-90	DQ04_00501100	Myo1 (TCDM_07314)
	1,00E-50	DQ04_03771000	MyoG (TCDM_02016)
Trypanosoma rangeli SC58	0.0	TRSC58_01895	MyoF (TCDM_08875)
	2,00E-159	TRSC58_00243	MyoE (TCDM_06166)
	2,00E-116	TRSC58_06141	MyoD (TCDM_07686)
	4,00E-17	TRSC58_00411	MyoB (TCDM_07433)
Trypanosoma vivax Y486	8,00E-114	TvY486_1117220	Myo13 (TCDM_05821)
	3,00E-90	TvY486_0403180	Myo1 (TCDM_07314)

**NCBI**			
**Genomes**	**BlastP e-value**	**ID of Hit**	**Reciprocal Blast**
**Green boxes are hits that correspond to MyoF genes**			
**Kinetoplastids**			
Trypanosoma theileri	0.0	ORC89982.1	MyoF (TCDM_08875)
	8,00E-175	ORC90398.1	MyoC (TCDM_02877)
	3,00E-174	ORC88651.1	MyoB (TCDM_07433)
	4,00E-165	ORC86402.1	MyoE (TCDM_06166)
	2,00E-162	ORC93297.1	MyoA (TCDM_09957)
	1,00E-120	ORC86814.1	MyoD (TCDM_07686)
	2,00E-104	ORC87480.1	Myo13 (TCDM_05821)
	4,00E-82	ORC93509.1	Myo1 (TCDM_07314)
	3,00E-53	ORC91827.1	MyoG (TCDM_02016)
Angomonas deanei	2,00E-113	EPY29051.1	Myo13 (TCDM_05821)
	3,00E-110	EPY42972.1	Myo13 (TCDM_05821)
	6,00E-68	EPY31408.1	Myo1 (TCDM_07314)

	4,00E-28	EPY36601.1	Myo13 (TCDM_05821)
Strigomonas culicis	4,00E-105	EPY36094.1	Myo13 (TCDM_05821)
	1,00E-103	EPY22327.1	Myo13 (TCDM_05821)
	4,00E-28	EPY17541.1	Myo1 (TCDM_07314)
	1,00E-24	EPY17540.1	Myo1 (TCDM_07314)
Phytomonas sp. isolate EM1	2,00E-102	CCW60276.1	Myo13 (TCDM_05821)
Phytomonas sp. isolate Hart1	3,00E-102	CCW68456.1	Myo13 (TCDM_05821)
Bodo saltans	0.0	CUG91579.1	MyoF (TCDM_08875)
	3,00E-174	CUG93737.1	MyoB (TCDM_07433)
	4,00E-170	CUG87167.1	MyoB (TCDM_07433)
	3,00E-167	CUG89451.1	MyoA (TCDM_09957)
	3,00E-163	CUF67578.1	MyoB (TCDM_07433)
	6,00E-161	CUG90281.1	MyoB (TCDM_07433)
	6,00E-160	CUF99905.1	MyoE (TCDM_06166)
	4,00E-131	CUG92185.1	MyoB (TCDM_07433)
	2,00E-128	CUG03397.1	MyoC (TCDM_02877)
	1,00E-109	CUG03103.1	Myo13 (TCDM_05821)
	1,00E-94	CUF17710.1	MyoD (TCDM_07686)
	5,00E-86	CUI15073.1	Myo1 (TCDM_07314)
	8,00E-52	CUG86977.1	MyoG (TCDM_02016)
**Examples of BlastP result against non-kinetoplastid species**			
Naegleria gruberi	1,00E-151	XP_002680898.1	MyoC (TCDM_02877)
Naegleria gruberi	2,00E-134	XP_002681567.1	MyoB (TCDM_07433)
Leptosomus discolor	7,00E-123	XP_009951855.1	MyoA (TCDM_09957)
Brassica rapa	3,00E-122	XP_009101754.2	MyoC (TCDM_02877)
Pygoscelis adeliae	6,00E-122	KFW66422.1	MyoA (TCDM_09957)
Pelecanus crispus	9,00E-122	KFQ66526.1	MyoA (TCDM_09957)
Saccharomyces cerevisiae	4,00E-121	CAY86606.1	MyoA (TCDM_09957)
Zygosaccharomyces rouxii	2,00E-120	XP_002496613.1	MyoA (TCDM_09957)
Haliaeetus albicilla	1,00E-118	KFQ10165.1	MyoA (TCDM_09957)
Ricinus communis	2,00E-118	XP_015575858.1	MyoA (TCDM_09957)

Table S20: Synteny conservation analysis of MyoF gene

Genome	Upstream gene	MyoF gene	Downstream gene from *T. grayi*	Downstream gene from *T. cruzi*
**Green boxes highlight the species that have MyoF gene in syntenic conserved region in relation to *T. cruzi* MyoF**				
Blechomonas ayalai B08-376	Baya_270_0010	no orthologous found	end of contig	Non-syntenic
Crithidia fasciculata strain Cf-Cl	CFAC1_120006600	no remnants	CFAC1_120006500	Non-syntenic
Endotrypanum monterogeii strain LV88	EMOLV88_160005000	no orthologous found	end of contig	Non-syntenic
Leishmania aethiopica L147	LAEL147_000224400	no remnants	LAEL147_000224300	Non-syntenic
Leishmania arabica strain LEM1108	LARLEM1108_160005100	no remnants	LARLEM1108_160005000	Non-syntenic
Leishmania braziliensis MHOM/BR/75/M2903	LBRM2903_160005300	no remnants	LBRM2903_160005200	Non-syntenic
Leishmania braziliensis MHOM/BR/75/M2904	LbrM.16.0020	no remnants	LbrM.16.0010	Non-syntenic
Leishmania donovani BPK282A1	LdBPK_160020.1	no remnants	LdBPK_160010.1	Non-syntenic
Leishmania enriettii strain LEM3045	LENLEM3045_160005300	no remnants	LENLEM3045_160005200	Non-syntenic
Leishmania gerbilli strain LEM452	LGELEM452_160005100	no remnants	LGELEM452_160005000	Non-syntenic
Leishmania infantum JPCM5	LinJ.16.0020	no remnants	LinJ.16.0010	Non-syntenic
Leishmania major strain Friedlin	LmjF.16.0010	no remnants	LmjF.16.0005	Non-syntenic
Leishmania major strain LV39c5	LMJLV39_160005100	no remnants	LMJLV39_160005000	Non-syntenic
Leishmania major strain SD 75.1	LMJSD75_160005100	no remnants	MJSD75_160005000	Non-syntenic
Leishmania mexicana MHOM/GT/2001/U1103	LmxM.16.0010	no remnants	LmxM.16.0005	Non-syntenic
Leishmania panamensis MHOM/COL/81/L13	LPAL13_160005100	no remnants	LPAL13_160005000	Non-syntenic
Leishmania tarentolae Parrot-TarII	LtaP16.0020	no remnants	LtaP16.0010	Non-syntenic
Leishmania tropica L590	LTRL590_160005100	no remnants	LTRL590_160005000	Non-syntenic
Leishmania turanica strain LEM423	LTULEM423_160005100	no remnants	LTULEM423_160005000	Non-syntenic
Leishmania sp. MAR LEM2494	LMARLEM2494_160005100	no remnants	LMARLEM2494_160005000	Non-syntenic
Leptomonas pyrrhocoris	LpyrH10_15_0040	no remnants	LpyrH10_15_0030	Non-syntenic
Leptomonas seymouri ATCC 30220	Lsey_0026_0470	no remnants	Lsey_0026_0480	Non-syntenic
Trypanosoma brucei Lister strain 427	Tb427.05.3980	no remnants	Tb427.08.5890	Non-syntenic
Trypanosoma brucei brucei TREU927	Tb927.5.3980	no remnants	Tb927.8.5890	Non-syntenic
Trypanosoma brucei gambiense DAL972	Tbg972.5.5490	no remnants	Tbg972.8.5890	Non-syntenic
Trypanosoma congolense IL3000	TcIL3000_5_4530	no remnants	TcIL3000_1_170	Non-syntenic
Trypanosoma cruzi CL Brener Esmeraldo-like	TcCLB.506947.100	TcCLB.506947.110:pseudogenic transcript	TcCLB.503713.10	Non-syntenic
Trypanosoma cruzi CL Brener Non-Esmeraldo-like	TcCLB.507057.10	TcCLB.507057.4	TcCLB.503945.20	Non-syntenic
Trypanosoma cruzi Dm28c	TCDM_08876	TCDM_08875	TCDM_13915	TCDM_08874
Trypanosoma cruzi marinkellei strain B7	Tc_MARK_4645	Tc_MARK_4644	Tc_MARK_25	Non-syntenic
Trypanosoma evansi strain STIB 805	TevSTIB805.5.4540	no remnants	TevSTIB805.8.6150	Non-syntenic
Trypanosoma grayi ANR4	DQ04_03341040	DQ04_03341050	DQ04_03341060	Non-syntenic
Trypanosoma rangeli SC58*	TRSC58_07164*	no orthologous found	TRSC58_05632*	Non-syntenic
Trypanosoma vivax Y486	TvY486_0503430	no orthologous found	TvY486_0805410	Non-syntenic
Trypanosoma theileri isolate Edinburgh TM35_Tth_5_V1[#]	NBCO01000010.1:458129-459250	NBCO01000010.1:459685-464112	NBCO01000008.1:900154-900618	na
Bodo saltans[#]	CYKH01001941.1:35800-36921	CYKH01001941.1:37141-41010	CYKH01001941.1:45078-45482	na
Angomonas deanei strain ATCC PRA-265[#]	KV452495.1:22895-24001	no remnants	KV452495.1:22098-22481	na
Strigomonas culicis strain TCC012E[#]	AUXH01000150.1:1275-2396	no remnants	AUXH01000150.1:286-729	na
Phytomas sp EM1[#]	HF955095.1:2132-3253	no remnants	HF955095.1:1372-1698	na
Phytomas sp Hart1[#]	HF955223.1:46081-47202	no remnants	HF955223.1:45351-45659	na

The IDs correspond to the homologous of *T. cruzi* Dm28c or *T. grayi* upstream or downstream genes. In some cases, they are not the nearest gene in the analyzed species.
* Evaluation of synteny for this species was not possible since the genes were located in very small contigs.
# For these species, the approximate chromosome position is presented. Genomes obtained from NCBI.
no orthologous found - we were not able to analyze the syntenic region and no orthologous gene was found throughout the genome.
no remnants- we were able to analyze the syntenic region and no consistent signals of the presence of gene was found.
na- not analyzed.

Table S21: Summary of BlastP results for MyoH-derived against sequences available at TritrypDB and NCBI

TritrypDB			
**Genomes**	**BlastP e-value**	**ID of Hit**	**Reciprocal Blast/Synteny**
**Green boxes are the hits that correspond to MyoH-derived genes**			
Blechomonas ayalai B08-376	7,00E-04	Baya_099_0300	Myo13 (TCDM_05821)
Crithidia fasciculata strain Cf-Cl	4,00E-07	CFAC1_300048900	Myo13 (TCDM_05821)
Endotrypanum monterogeii strain LV88	1,00E-09	EMOLV88_320042600	Myo13 (TCDM_05821)
Leishmania aethiopica L147	1,00E-06	LAEL147_000662900.1	Myo13 (TCDM_05821)
Leishmania arabica strain LEM1108	5,00E-06	LARLEM1108_320047000	Myo13 (TCDM_05821)
Leishmania braziliensis MHOM/BR/75/M2903	5,00E-08	LBRM2903_320050900	Myo13 (TCDM_05821)
Leishmania braziliensis MHOM/BR/75/M2904	1,00E-08	LbrM.32.4110	Myo13 (TCDM_05821)
Leishmania donovani BPK282A1	5,00E-07	LdBPK_324020.1	Myo13 (TCDM_05821)
Leishmania enriettii strain LEM3045	1,00E-07	LENLEM3045_320047100	Myo13 (TCDM_05821)
Leishmania gerbilli strain LEM452	7,00E-07	LGELEM452_320047000	Myo13 (TCDM_05821)
Leishmania infantum JPCM5	1,00E-06	LinJ.32.4020	Myo13 (TCDM_05821)
Leishmania major strain Friedlin	3,00E-06	LmjF.32.3870	Myo13 (TCDM_05821)
Leishmania major strain LV39c5	4,00E-06	LMJLV39_320046600	Myo13 (TCDM_05821)
Leishmania major strain SD 75.1	3,00E-06	LMJSD75_320046800	Myo13 (TCDM_05821)
Leishmania mexicana MHOM/GT/2001/U1103	2,00E-07	LmxM.31.3870	Myo13 (TCDM_05821)
Leishmania panamensis MHOM/COL/81/L13	4,00E-09	LPAL13_320047300	Myo13 (TCDM_05821)
Leishmania tarentolae Parrot-TarII	5,00E-06	LtaP32.4040	Myo13 (TCDM_05821)
Leishmania tropica L590	5,00E-07	LTRL590_320046900	Myo13 (TCDM_05821)
Leishmania turanica strain LEM423	7,00E-07	LTULEM423_320047500	Myo13 (TCDM_05821)
Leishmania sp. MAR LEM2494	1,00E-05	LMARLEM2494_320047400	Myo13 (TCDM_05821)
Leptomonas pyrrhocoris	1,00E-05	LpyrH10_02_4330	Myo13 (TCDM_05821)
Leptomonas seymouri ATCC 30220	7,00E-05	Lsey_0246_0030	Myo13 (TCDM_05821)
Trypanosoma brucei Lister strain 427	4,00E-07	Tb427tmp.01.7990	Myo13 (TCDM_05821)
Trypanosoma brucei brucei TREU927	7,00E-07	Tb927.11.16310	Myo13 (TCDM_05821)
Trypanosoma brucei gambiense DAL972	2,00E-06	Tbg972.11.18310	Myo13 (TCDM_05821)
Trypanosoma congolense IL3000	No hits found		
Trypanosoma cruzi marinkellei strain B7	0.0	Tc_MARK_2031	MyoH-derived (TCDM_02145)
	3,00E-07	Tc_MARK_4039	Myo13 (TCDM_05821)
Trypanosoma evansi strain STIB 805	1,00E-07	TevSTIB805.11_01.16880	Myo13 (TCDM_05821)
Trypanosoma grayi ANR4	0.0	DQ04_00271170	MyoH-derived (TCDM_02145)
	0.001	DQ04_00031100	Myo13 (TCDM_05821)

Trypanosoma rangeli SC58	0.0	TRSC58_00453	MyoH-derived (TCDM_02145)
Trypanosoma vivax Y486	3,00E-07	TvY486_1117220	Myo13 (TCDM_05821)
**NCBI**			
**Genomes**	**BlastP e-value**	**ID of Hit**	**Reciprocal Blast**
**Green boxes are hits that correspond to MyoH-derived genes**			
**Kinetoplastids**			
Trypanosoma theileri	0.0	ORC90782.1	MyoH-derived (TCDM_02145)
Angomonas deanei	No hits found		
Strigomonas culicis	No hits found		
Phytomonas sp. isolate EM1	No hits found		
Phytomonas sp. isolate Hart1	No hits found		
Bodo saltans	3,00E-167	CUF84730.1	MyoH-derived (TCDM_02145)
**Unique Hit of BlastP result from other species – non significant**			
Branchiostoma belcheri	0.89	XP_019640798.1	TCDM_10054 hypothetical protein (non-myosin)

Table S22:Synteny conservation analysis of MyoH-derived gene

Organism	Upstream gene 2	Upstream gene 1	MyoH-derived gene	Downstream gene 1 from CL Brener
**Green boxes highlight the species that have MyoH-derived gene in syntenic conserved region in relation to *T. cruzi* MyoH-derived gene**				
Blechomonas ayalai B08-376	Baya_060_0380	Baya_060_0390	no remnants	Baya_060_0400
Crithidia fasciculata strain Cf-Cl	CFAC1_260053100	CFAC1_260053000	no remnants	CFAC1_260052900
Endotrypanum monterogeii strain LV88	EMOLV88_300034200	EMOLV88_300034100	no remnants	EMOLV88_300034000
Leishmania aethiopica L147	LAEL147_000576900	no homologous found	no remnants	LAEL147_000576700
Leishmania arabica strain LEM1108	LARLEM1108_300035000	no homologous found	no remnants	LARLEM1108_300034800
Leishmania braziliensis MHOM/BR/75/M2903	LBRM2903_300035100	LBRM2903_300035000	no remnants	LBRM2903_300034800
Leishmania braziliensis MHOM/BR/75/M2904	LbrM.30.2840	no homologous found	no remnants	LbrM.30.2830
Leishmania donovani BPK282A1	LdBPK_302880.1	no homologous found	no remnants	LdBPK_302870.1
Leishmania enriettii strain LEM3045	LENLEM3045_300034800	LENLEM3045_300034700	no remnants	LENLEM3045_300034600
Leishmania gerbilli strain LEM452	LGELEM452_300035600	no homologous found	no remnants	Non-syntenic
Leishmania infantum JPCM5	LinJ.30.2880	no homologous found	no remnants	LinJ.30.2870
Leishmania major strain Friedlin	LmjF.30.2860	no homologous found	no remnants	LmjF.30.2850
Leishmania major strain LV39c5	LMJLV39_300036400	no homologous found	no remnants	LMJLV39_300036200
Leishmania major strain SD 75.1	LMJSD75_300035700	no homologous found	no remnants	LMJSD75_300035600
Leishmania mexicana MHOM/GT/2001/U1103	no homologous found	no homologous found	no remnants	LmxM.29.2850
Leishmania panamensis MHOM/COL/81/L13	LPAL13_300032800	LPAL13_300032700	no remnants	LPAL13_300032600
Leishmania tarentolae Parrot-TarII	LtaP30.2860	no homologous found	no remnants	LtaP30.2850
Leishmania tropica L590	LTRL590_300041800	no homologous found	no remnants	LTRL590_300034900
Leishmania turanica strain LEM423	LTULEM423_300035000	no homologous found	no remnants	LTULEM423_300034900
Leishmania sp. MAR LEM2494	LMARLEM2494_300034800	LMARLEM2494_300034700	no remnants	LMARLEM2494_300034500.1
Leptomonas pyrrhocoris	LpyrH10_04_5680	LpyrH10_04_5670	no remnants	LpyrH10_04_5660
Leptomonas seymouri ATCC 30220	Lsey_0209_0120	Lsey_0209_0130	no remnants	Lsey_0209_0140
Trypanosoma brucei Lister strain 427	Tb427.06.4160	Tb427.06.4150	no remnants	Tb427.06.4140
Trypanosoma brucei brucei TREU927	Tb927.6.4160	Tb927.6.4150	no remnants	Tb927.6.4140
Trypanosoma brucei gambiense DAL972	Tbg972.6.3950	Tbg972.6.3940	no remnants	Tbg972.6.3930
Trypanosoma congolense IL3000	TcIL3000_6_3570	TcIL3000_6_3560.1	no remnants	TcIL3000_6_3540
Trypanosoma cruzi CL Brener Esmeraldo-like	TcCLB.511733.60	TcCLB.511733.70	TcCLB.511733.80	TcCLB.511733.90
Trypanosoma cruzi CL Brener Non-Esmeraldo-like	TcCLB.511523.50	TcCLB.511523.40	TcCLB.511523.30	TcCLB.511523.20
Trypanosoma cruzi Dm28c	TCDM_02143	TCDM_02144	TCDM_02145	TCDM_013970
Trypanosoma cruzi marinkellei strain B7	Tc_MARK_2032	no homologous found	Tc_MARK_2031	no homologous found
Trypanosoma evansi strain STIB 805	TevSTIB805.6.4290	TevSTIB805.6.4280	no remnants	TevSTIB805.6.4270
Trypanosoma grayi ANR4	DQ04_00271190	DQ04_00271180	DQ04_00271170	DQ04_00271160
Trypanosoma rangeli SC58*	TRSC58_05120	TRSC58_07348	TRSC58_00453	TRSC58_00701
Trypanosoma vivax Y486	Non-syntenic	no homologous found	no orthologous found	TvY486_1010980
Trypanosoma theileri isolate Edinburgh TM35_Tth_5_V1#	NBCO01000007.1:206684 - 207733	NBCO01000007.1:208184-208771	NBCO01000007.1:209342-212836	NBCO01000007.1 :213516-213860
Bodo saltans#	CYKH01000420.1:87720-88256	no homologous found	CYKH01000420.1:88543-92124	CYKH01000420.1:92450-92791

The IDs correspond to the homologous of *T. cruzi* Dm28c or CL Brener upstream or downstream genes. In some cases, they are not the nearest gene in the analyzed species.
* Evaluation of synteny for this species was not possible since the genes were located in very small contigs.
\# For these species, the approximate chromosome position is presented. Genomes obtained from NCBI.
no orthologous found - we were not able to analyze the syntenic region and no orthologous gene was found throughout the genome.
no remnants- we were able to analyze the syntenic region and no consistent signals of the presence of gene was found.
